# Supplementary material for: Associations of PFAS and OH-PCBs with risk of multiple sclerosis onset and disability worsening
Source: Nat Commun. 2025 Feb 27;16:2014. doi: 10.1038/s41467-025-57172-3 (PMC11868641; doi:10.1038/s41467-025-57172-3)
Supplement: Supplementary file 1 — Supplementary information [file 41467_2025_57172_MOESM1_ESM.pdf]

Supplementary Information

- **Supplementary Table S1.** Per- and polyfluorinated substance (PFAS) and hydroxyl polychlorinated biphenyls (OH-PCBs) information.
- **Supplementary Table S2.** Per- and polyfluorinated substances (PFAS) and hydroxyl polychlorinated biphenyls (OH-PCBs) concentration differences in males (n = 458) and females (n = 1356), as well as between individuals with and without biological children stratified by sex.
- **Supplementary Table S3.** Paired comparison of per- and polyfluorinated substances (PFAS) and hydroxyl polychlorinated biphenyls (OH-PCBs) in multiple sclerosis (MS) patients and matched control (HC) subjects.
- **Supplementary Table S4.** Coefficients for mixed-linear regression model used for the paired comparison of per- and polyfluorinated substances (PFAS) and hydroxyl polychlorinated biphenyls (OH-PCBs) in multiple sclerosis (MS) patients (n = 907) and matched control (HC) subjects (n = 907).
- **Supplementary Table S5.** ANOVA F values and degrees of freedom for mixed-linear regression model used for the paired comparison of per- and polyfluorinated substances (PFAS) and hydroxyl polychlorinated biphenyls (OH-PCBs) in multiple sclerosis (MS) patients (n = 907) and matched control (HC) subjects (n = 907).
- **Supplementary Table S6.** Paired comparison of per- and polyfluorinated substances (PFAS) and hydroxyl polychlorinated biphenyls (OH-PCBs) in multiple sclerosis (MS) patients and matched control (HC) subjects divided in males and females.
- **Supplementary Table S7.** Coefficients for mixed-linear regression model used in the sensitivity analysis of the paired comparison of per- and polyfluorinated substances (PFAS) and hydroxyl polychlorinated biphenyls (OH-PCBs) in multiple sclerosis (MS) patients (n = 907) and matched control (HC) subjects (n = 907), considering whether they were born in Swedish or not.
- **Supplementary Table S8.** ANOVA F values and degrees of freedom for mixed-linear regression model used in the sensitivity analysis of the paired comparison of per- and polyfluorinated substances (PFAS) and hydroxyl polychlorinated biphenyls (OH-PCBs) in multiple sclerosis (MS) patients (n = 907) and matched control (HC) subjects (n = 907), considering whether they were born in Swedish or not.
- **Supplementary Table S9.** Paired comparison of per- and polyfluorinated substances (PFAS) and hydroxyl polychlorinated biphenyls (OH-PCBs) in multiple sclerosis (MS) patients and matched control (HC) subjects, considering whether they were born in Swedish or not.
- **Supplementary Table S10.** Paired comparison of per- and polyfluorinated substances (PFAS) and hydroxyl polychlorinated biphenyls (OH-PCBs) in multiple sclerosis (MS) patients and matched control (HC) subjects divided in males and females, considering whether they were born in Swedish or not.
- **Supplementary Table S11.** Coefficients for mixed-linear regression model used in the sensitivity analysis of the paired comparison of per- and polyfluorinated substances (PFAS) and hydroxyl polychlorinated biphenyls (OH-PCBs) in multiple sclerosis (MS) patients (n =907) and matched control (HC) subjects (n = 907), considering whether they were born in a Nordic country or not.
- **Supplementary Table S12.** ANOVA F values and degrees of freedom for mixed-linear regression model used in the sensitivity analysis of the paired comparison of per- and polyfluorinated substances (PFAS) and hydroxyl polychlorinated biphenyls (OH-PCBs) in multiple sclerosis (MS) patients (n = 907) and matched control (HC) subjects (n = 907), considering whether they were born in a Nordic country or not.
- **Supplementary Table S13.** Paired comparison of per- and polyfluorinated substances (PFAS) and hydroxyl polychlorinated biphenyls (OH-PCBs) in multiple sclerosis (MS) patients and matched control (HC) subjects, considering whether they were born in a Nordic country or not.
- **Supplementary Table S14.** Paired comparison of per- and polyfluorinated substances (PFAS) and hydroxyl polychlorinated biphenyls (OH-PCBs) in multiple sclerosis (MS) patients and matched control (HC) subjects divided in males and females, considering whether they were born in a Nordic country or not.
- **Supplementary Table S15.** Coefficients for mixed-linear regression model used in the sensitivity analysis of the paired comparison of per- and polyfluorinated substances (PFAS) and hydroxyl polychlorinated biphenyls (OH-PCBs) in multiple sclerosis (MS) patients (n = 907) and matched control (HC) subjects (n = 907), with the residential area criteria removed.
- **Supplementary Table S16.** ANOVA F values and degrees of freedom for mixed-linear regression model used in the sensitivity analysis of the paired comparison of per- and polyfluorinated substances (PFAS) and hydroxyl polychlorinated biphenyls (OH-PCBs) in multiple sclerosis (MS) patients (n = 907) and matched control (HC) subjects (n = 907), with the residential area criteria removed.
- **Supplementary Table S17.** Paired comparison of per- and polyfluorinated substances (PFAS) and hydroxyl polychlorinated biphenyls (OH-PCBs) in multiple sclerosis (MS) patients and matched control (HC) subjects, with the residential area criteria removed.
- **Supplementary Table S18.** Paired comparison of per- and polyfluorinated substances (PFAS) and hydroxyl polychlorinated biphenyls (OH-PCBs) in multiple sclerosis (MS) patients and matched control (HC) subjects divided in males and females, with the residential area criteria removed.

- **Supplementary Table S19.** Associations between single serum per- and polyfluorinated substances (PFAS) and hydroxyl polychlorinated biphenyls (OH-PCBs), and the odds ratio of multiple sclerosis (MS) based on the Base model (n = 1,814).
- **Supplementary Table S20.** Coefficients for the logistic regression base model used for evaluating per- and polyfluorinated substances (PFAS) and hydroxyl polychlorinated biphenyls (OH-PCBs) association with odds of multiple sclerosis (MS), males and females combined (n = 1,814).
- **Supplementary Table S21.** Coefficients for the logistic regression Base model used for evaluating per- and polyfluorinated substances (PFAS) and hydroxyl polychlorinated biphenyls (OH-PCBs) association with odds of multiple sclerosis (MS), in males (n = 458).
- **Supplementary Table S22.** Coefficients for the logistic regression Base model used for evaluating per- and polyfluorinated substances (PFAS) and hydroxyl polychlorinated biphenyls (OH-PCBs) association with odds of multiple sclerosis (MS), in females (n = 1,356).
- **Supplementary Table S23.** Coefficients for the logistic regression for the fully adjusted model used for evaluating per- and polyfluorinated substances (PFAS) and hydroxyl polychlorinated biphenyls (OH-PCBs) association with odds of multiple sclerosis (MS), males and females (n = 1,814).
- **Supplementary Table S24.** Coefficients for the logistic regression for the fully adjusted model used for evaluating per- and polyfluorinated substances (PFAS) and hydroxyl polychlorinated biphenyls (OH-PCBs) association with odds of multiple sclerosis (MS), in males (n = 458).
- **Supplementary Table S25.** Coefficients for the logistic regression for the fully adjusted model used for evaluating per- and polyfluorinated substances (PFAS) and hydroxyl polychlorinated biphenyls (OH-PCBs) association with odds of multiple sclerosis (MS), in females (n = 1,356).
- **Supplementary Table S26.** Coefficients for the logistic regression for the fully adjusted model including Nordic born status used for evaluating per- and polyfluorinated substances (PFAS) and hydroxyl polychlorinated biphenyls (OH-PCBs) association with odds of multiple sclerosis (MS), males and females (n = 1,814).
- **Supplementary Table S27.** Coefficients for the logistic regression for the fully adjusted model including Nordic born status used for evaluating per- and polyfluorinated substances (PFAS) and hydroxyl polychlorinated biphenyls (OH-PCBs) association with odds of multiple sclerosis (MS), in males (n = 458).
- **Supplementary Table S28.** Coefficients for the logistic regression for the fully adjusted model including Nordic born used for evaluating per- and polyfluorinated substances (PFAS) and hydroxyl polychlorinated biphenyls (OH-PCBs) association with odds of multiple sclerosis (MS), in females (n = 1,356).
- **Supplementary Table S29.** Associations between single serum per- and polyfluorinated substances (PFAS) and hydroxyl polychlorinated biphenyls (OH-PCBs), and the odds ratio of multiple sclerosis (MS) (n = 1,814).
- **Supplementary Table S30.** Coefficients for the logistic regression for the fully adjusted model, residential area not taken into account, used for evaluating per- and polyfluorinated substances (PFAS) and hydroxyl polychlorinated biphenyls (OH-PCBs) association with odds of multiple sclerosis (MS), males and females (n = 1,814).
- **Supplementary Table S31.** Coefficients for the logistic regression for the fully adjusted model, residential area not taken into account, used for evaluating per- and polyfluorinated substances (PFAS) and hydroxyl polychlorinated biphenyls (OH-PCBs) association with odds of multiple sclerosis (MS), in males (n = 458).
- **Supplementary Table S32.** Coefficients for the logistic regression for the fully adjusted model, residential area not taken into account, used for evaluating per- and polyfluorinated substances (PFAS) and hydroxyl polychlorinated biphenyls (OH-PCBs) association with odds of multiple sclerosis (MS), in females (n = 1,356).
- **Supplementary Table S33.** Associations between single serum per- and polyfluorinated substances (PFAS) and hydroxyl polychlorinated biphenyls (OH-PCBs), and the odds ratio of multiple sclerosis (MS) (n = 1,814).
- **Supplementary Table S34.** General and clinical characteristics for the 551 individuals included in the Cox proportional hazard analysis regards confirmed disability worsening (CDW).
- **Supplementary Table S35.** Cox proportional hazard analysis output from confirmed disability worsening analysis in males and females (n = 551).
- **Supplementary Table S36.** Cox proportional hazard analysis output from confirmed disability worsening analysis in males (n = 137).
- **Supplementary Table S37.** Cox proportional hazard analysis output from confirmed disability worsening analysis in females (n = 414).
- **Supplementary Table S38.** Cox proportional hazard analysis output from confirmed disability worsening analysis in males and females (n = 551), adjusting for treatment during the follow-up time.
- **Supplementary Table S39.** Cox proportional hazard analysis output from confirmed disability worsening analysis in males (n = 137), adjusting for treatment during the follow-up time.
- **Supplementary Table S40.** Cox proportional hazard analysis output from confirmed disability worsening analysis in females (n = 414), adjusting for treatment during the follow-up time.
- **Supplementary Table S41.** Cox proportional hazard analysis for serum albumin concentrations association with confirmed disability worsening.

- **Supplementary Table S42.** Cox proportional hazard analysis output from confirmed disability worsening analysis in males and females (n = 551), adjusting for serum albumin concentrations.
- **Supplementary Table S43.** Cox proportional hazard analysis output from confirmed disability worsening analysis in males (n = 137), adjusting for serum albumin concentrations.
- **Supplementary Table S44.** Cox proportional hazard analysis output from confirmed disability worsening analysis in females (n = 414), adjusting for serum albumin concentrations.
- **Supplementary Table S45.** Cox proportional hazard analysis output from relapsing-remitting multiple sclerosis (RRMS) to secondary-progressive multiple sclerosis (SPMS) transition analysis (n = 801).
- **Supplementary Table S46.** Cox proportional hazard analysis output from relapsing-remitting multiple sclerosis (RRMS) to secondary-progressive multiple sclerosis (SPMS) transition analysis (n = 801), adjusting for treatment during the follow-up time.
- **Supplementary Table S47.** Cox proportional hazard analysis output from relapsing-remitting multiple sclerosis (RRMS) to secondary-progressive multiple sclerosis (SPMS) transition analysis (n = 801), adjusting for serum albumin concentrations.
- **Supplementary Table S48.** Per- and polyfluorinated substances (PFAS) and hydroxyl polychlorinated biphenyls (OH-PCBs) two-tailed Spearman rank correlation test with 25(OH)D3.
- **Supplementary Table S49.** Cox proportional hazard analysis for 25(OH)D3 serum levels association with confirmed disability worsening.
- **Supplementary Table S50.** Cox proportional hazard analysis for per- and polyfluorinated substances (PFAS) and hydroxyl polychlorinated biphenyls (OH-PCBs) association with confirmed disability worsening adjusting for 25(OH)D3 levels (n = 551).
- **Supplementary Table S51.** Cox proportional hazard analysis for per- and polyfluorinated substances (PFAS) and hydroxyl polychlorinated biphenyls (OH-PCBs) association with confirmed disability worsening in males (n = 137), adjusting for 25(OH)D3 levels.
- **Supplementary Table S52.** Cox proportional hazard analysis for per- and polyfluorinated substances (PFAS) and hydroxyl polychlorinated biphenyls (OH-PCBs) association with confirmed disability worsening in females (n = 414), adjusting for 25(OH)D3 levels.
- **Supplementary Table S53.** Cox proportional hazard analysis for 25(OH)D3 serum levels association with the transition from relapsing-remitting multiple sclerosis (RRMS) to secondary-progressive multiple sclerosis (SPMS) (n = 801).
- **Supplementary Table 54.** Cox proportional hazard analysis for per- and polyfluorinated substances (PFAS) and hydroxyl polychlorinated biphenyls (OH-PCBs) association with the transition from relapsing-remitting multiple sclerosis (RRMS) to secondary-progressive multiple sclerosis (SPMS) (n = 801), adjusting for 25(OH)D3 levels.
- **Supplementary Table S55.** *P* values for per- and polyfluorinated substances (PFAS) and hydroxyl polychlorinated biphenyls (OH-PCBs) correlations.
- **Supplementary Table S56.** Correlation coefficient for per- and polyfluorinated substances (PFAS) and hydroxyl polychlorinated biphenyls (OH-PCBs) correlations.
- **Supplementary Table S57.** Description of treatments included in each treatment category.
- **Supplementary Figure S1.** Per- and polyfluorinated substance (PFAS) and hydroxyl polychlorinated biphenyls (OH-PCB) concentration (log_2 ng/mL) versus sample collection year of persons with multiple sclerosis (MS) and matched controls (HC), separated by sex.
- **Supplementary Figure S2.** Per- and polyfluorinated substance (PFAS) and hydroxyl polychlorinated biphenyls (OH-PCB) concentration (log_2 ng/mL) versus age of persons with multiple sclerosis (MS) and matched controls (HC).
- **Supplementary Figure S3.** Per- and polyfluorinated substance (PFAS) and hydroxyl polychlorinated biphenyls (OH-PCB) concentration (log_2 ng/mL) in males and females.
- **Supplementary Figure S4**. Perfluorobutanesulfonic acid (PFBS), perfluoropentanesulfonic acid (PFPeS), and perfluoroheptanoic acid (PFHpA) concentration distribution across Sweden.
- **Supplementary Figure S5**. Perfluorohexanesulfonic acid (PFHxS), perfluoroheptanesulfonic acid (PFHpS), and perfluorooctanesulfonic acid (PFOS) concentration distribution across Sweden.
- **Supplementary Figure S6**. Perfluoro-1-octanesulfonamide (FOSA), N-methylperfluoro-1-octanesulfonamidoacetic acid (N-MeFOSAA), and N-ethylperfluoro-1-octanesulfonamidoacetic acid (N-EtFOSAA) concentration distribution across Sweden.
- **Supplementary Figure S7**. Perfluorononanoic acid (PFNA), perfluorodecanoic acid (PFDA), and perfluorodecane sulfonic acid (PFDS) concentration distribution across Sweden.
- **Supplementary Figure S8**. Perfluoroundecanoic acid (PFUnA), perfluorododecanoic acid (PFDoA), and perfluorotridecanoic acid (PFTrDA) concentration distribution across Sweden.

- **Supplementary Figure S9**. 1h,1h,2h,2h-perfluoro-1-decanesulfonic acid (8:2) (8:2 FTS), 2,2',3,4',5,5',6-heptachloro-4-biphenylol (4-OH-CB187), 2,2',4,4',5,5'-,Hexachloro-3-biphenylol (3-OH-CB153), and 2,3,3',4',5-Pentachloro-4-biphenylol (4-OH-CB107) concentration distribution across Sweden.
- **Supplementary Figure S10.** Concentration distribution of per- and polyfluorinated substances (PFAS) and hydroxyl polychlorinated biphenyls (OH-PCBs) in multiple sclerosis (MS) subjects and matched control (HC) divided by sex.
- **Supplementary Figure S11**. Per- and polyfluorinated substances (PFAS) and hydroxyl polychlorinated biphenyls (OH-PCBs) association with confirmed disability worsening adjusting for 25(OH)D3 levels.
- **Supplementary Figure S12**. Per- and polyfluorinated substances (PFAS) and hydroxyl polychlorinated biphenyls (OH-PCBs) association with the transition from relapsing-remitting multiple sclerosis (RRMS) to secondary progressive multiple sclerosis (SPMS) (n = 801) adjusted for 25(OH)D3 levels.
- **Supplementary Figure S13.** Spearman's rank correlation between per- and polyfluorinated substances (PFAS) and hydroxyl polychlorinated biphenyls (OH-PCBs).
- **Supplementary Figure S14.** Spearman's rank correlation between per- and polyfluorinated substances (PFAS) and hydroxyl polychlorinated biphenyls (OH-PCBs).

Supplementary Table S1. Per- and polyfluorinated substance (PFAS) and hydroxyl polychlorinated biphenyls (OH-PCBs) information. Compound abbreviation, calibration type, coverage (%), grouping, and mean concentration (ng/mL), including the standard deviation (SD) in matched control (HC) subjects (n = 907), relapsing-remitting multiple sclerosis (RRMS) (n = 801) and progressive multiple sclerosis (PMS) (n = 106). Two forms of calibration used are calibration curves and one-point calibration.

**Compound**	**Abbreviation**	**Calibration**	**Coverage (%)**	**Group**	**Concentration (ng/ml) mean (SD)**		
					HC	RRMS	PMS
**Perfluorobutanesulfonic acid**	PFBS	calibration curve	36	Short-chain PFAS	1.135e-02 (1.590e-02)	9.555e-03 (1.510e-02)	1.261e-02 (1.447e-02)
**Perfluoropentanesulfonic acid**	PFPeS	calibration curve	68	Short-chain PFAS	4.526e-02 (1.342e-01)	5.901e-02 (4.509e-01)	6.699e-02 (1.794e-01)
**Perfluoroheptanoic acid**	PFHpA	calibration curve	94	Short-chain PFAS	4.341e-02 (5.879e-02)	4.473e-02 (7.150e-02)	4.075e-02 (5.077e-02)
**Perfluorohexanesulfonic acid**	PFHxS	calibration curve	94	Long-chain PFAS	2.032 (3.580)	2.593 (21.130)	2.848 (4.325)
**Perfluoroheptanesulfonic acid**	PFHpS	calibration curve	96	Long-chain PFAS	2.339e-01 (1.949e-01)	2.715e-01 (1.108)	3.610e-01 (2.162e-01)
**Perfluorooctanoic acid**	PFOA	calibration curve	96	Long-chain PFAS	2.714 (1.780)	2.806 (3.379)	3.683 (2.652)
**Perfluorooctanesulfonic acid**	PFOS	calibration curve	95	Long-chain PFAS	9.432 (6.937)	9.813 (17.970)	12.970 (7.359)
**Perfluoro-1-octanesulfonamide**	FOSA	calibration curve	59	Long-chain PFAS	8.828e-03 (6.773e-03)	8.574e-03 (6.467e-03)	9.652e-03 (5.740e-03)
**N-methylperfluoro-1-octanesulfonamidoacetic acid**	N-MeFOSAA	calibration curve	97	Long-chain PFAS	8.378e-02 (1.061e-01)	9.134e-02 (1.214e-01)	1.033e-01 (1.074e-01)
**N-ethylperfluoro-1-octanesulfonamidoacetic acid**	N-EtFOSAA	calibration curve	42	Long-chain PFAS	4.373e-02 (5.420e-02)	4.503-02 (4.531e-02)	5.014e-02 (4.226e-02)
**Perfluorononanoic acid**	PFNA	calibration curve	96	Long-chain PFAS	9.278e-01 (1.908)	8.132e-01 (4.912e-01)	1.009 (0.770)
**Perfluorodecanoic acid**	PFDA	calibration curve	93	Long-chain PFAS	3.726e-01 (2.536e-01)	3.497e.01 (0.2143e-01)	4.205e-01 (3.043e-01)
**Perfluorodecane sulfonic acid**	PFDS	calibration curve	27	Long-chain PFAS	1.046e-02 (9.510e-03)	9.253e-03 (8.775e-03)	1.084e-02 (6.766e-03)
**Perfluoroundecanoic acid**	PFUnA	calibration curve	96	Long-chain PFAS	3.327e-01 (2.441e-01)	3.030e-01 (2.122e-02)	3.459e-01 (1.942e-01)
**Perfluorododecanoic acid**	PFDoA	calibration curve	97	Long-chain PFAS	4.432e-02 (3.204e-02)	3.980e-02 (2.772e-02)	4.440e-02 (2.755e-02)
**Perfluorotridecanoic acid**	PFTrDA	calibration curve	80	Long-chain PFAS	6.557e-02 (6.066e-02)	5.573e-02 (5.338e-02)	5.981e-02 (5.037e-02)
**1H,1H,2H,2H-perfluoro-1-decanesulfonic acid (8:2)**	8:2 FTS	calibration curve	76	FTS	3.271e-02 (4.132e-02)	3.391e-02 (4.581e-02)	3.008e-02 (3.484e-02)
**2,2',3,4',5,5',6-heptachloro-4-biphenylol**	4-OH-CB187	one-point calibration	90	OH-PCB	2.622e-02 (1.679e-02)	2.642e-02 (1.697e-02)	4.487e-02 (3.073e-02)
**2,2',4,4',5,5'-,Hexachloro-3-biphenylol**	3-OH-CB153	one-point calibration	35	OH-PCB	6.229e-01 (1.640)	6.686e-01 (1.315)	1.326 (1.783)
**2,3,3',4',5-Pentachloro-4-biphenylol**	4-OH-CB107	one-point calibration	41	OH-PCB	1.250e-01 (2.218e-01)	1.186e-01 (1.642e-01)	2.328e-01 (4.089e-01)

Supplementary Table S2. Per- and polyfluorinated substances (PFAS) and hydroxyl polychlorinated biphenyls (OH-PCBs) concentration differences in males (n = 458) and females (n = 1356), as well as between individuals with and without biological children stratified by sex. Log_2 fold changes (FC) were calculated using estimated marginal means based on a linear mixed-effect regression model and *P* values for the two-sided tests were computed using Satterthwaite's degrees of freedom method. No adjustments were made for multiple comparisons.

Compound	Females vs Males (ref. = males)		Biological children vs no biological children			
			Females		Males	
	Log_2 FC	*P* value	Log_2 FC	*P* value	Log_2 FC	*P* value
**PFBS**	0.466	4.86e-01	-0.644	1.41e-01	-0.166	8.22e-01
**PFPeS**	-0.013	2.46e-01	-1.132	1.23e-03	0.443	4.70e-01
**PFHpA**	0.377	6.04e-01	-0.751	8.45e-04	-0.618	1.05e-01
**PFHxS**	-0.666	1.37e-09	-0.460	3.13e-07	0.189	2.90e-01
**PFHpS**	-0.844	5.30e-10	-0.473	1.10e-07	0.059	6.54e-01
**PFOA**	-0.284	2.54e-04	-0.539	2.98e-14	-0.050	6.08e-01
**PFOS**	-0.527	9.75e-05	-0.248	3.39e-03	0.013	9.30e-01
**FOSA**	-0.008	8.58e-01	-0.044	5.95e-01	-0.055	6.91e-01
**N-MeFOSAA**	-0.056	4.18e-01	-0.092	3.42e-01	-0.087	5.96e-01
**N-EtFOSAA**	-0.016	3.75e-01	-0.161	1.13e-01	-0.125	4.58e-01
**PFNA**	-0.151	1.03e-01	-0.359	1.11e-05	-0.138	3.71e-01
**PFDA**	-0.148	1.42e-01	-0.178	5.21e-02	0.045	7.26e-01
**PFDS**	0.070	7.16e-01	-0.067	5.72e-01	0.149	4.30e-01
**PFUnA**	0.186	1.11e-01	-0.135	1.69e-01	0.207	1.82e-01
**PFDoA**	0.188	1.55e-01	-0.205	1.39e-02	-0.255	6.98e-01
**PFTrDA**	0.393	2.59e-02	-0.072	6.61e-01	0.122	6.70e-01
**8:2 FTS**	0.391	1.68e-02	-0.054	7.27e-01	-0.093	7.05e-01
**4-OH-CB187**	-0.324	4.54e-02	0.024	8.45e-01	-0.302	1.61e-01
**3-OH-CB153**	-0.024	9.25e-01	-0.400	2.81e-02	0.051	8.69e-01
**4-OH-CB107**	0.099	8.54e-01	-0.399	7.20e-03	-0.299	3.53e-01

The model females vs. males was adjusted for age, BMI at sample collection, BMI at the age of 20, the year the blood sample was taken, years of regular smoking, years of irregular smoking, years of passive smoking (indoors), type of undergoing treatment, number of childbirths, and disease phenotype.

The model biological children vs no biological children was adjusted for age, BMI at sample collection, BMI at the age of 20, the year the blood sample was taken, years of regular smoking, years of irregular smoking, years of passive smoking (indoors), type of undergoing treatment, and disease phenotype.

Supplementary Table S3. Paired comparison of per- and polyfluorinated substances (PFAS) and hydroxyl polychlorinated biphenyls (OH-PCBs) in multiple sclerosis (MS) patients and matched control (HC) subjects. Comparisons have been made between the entire MS group (n = 907) and HC subjects (n = 907), between patients with relapsing-remitting MS (RRMS) (n = 801) and HC subjects (n = 801), and between progressive MS (PMS) (n = 106) and HC subjects (n = 106). Log_2 fold changes (FC) were calculated using estimated marginal means based on a linear mixed-effect regression model and *P* values for the two-sided tests were computed using Satterthwaite's degrees of freedom method. No adjustments were made for multiple comparisons.

**Compound**	**MS vs HC**		**RRMS vs HC**		**PMS vs HC**	
	**Log_2 FC**	***P* value**	**Log_2 FC**	***P* value**	**Log_2 FC**	***P* value**
**PFBS**	-2.57e-02	9.62e-01	-3.98e-02	9.16e-01	-1.87e-02	9.80e-01
**PFPeS**	-8.09e-01	6.12e-02	-3.76e-01	2.10e-01	-1.03	9.12e-02
**PFHpA**	-7.93e-01	6.18e-03	-2.50e-01	2.22e-01	-1.06	8.05e-03
**PFHxS**	2.03e-02	8.50e-01	-1.44e-01	5.12e-02	1.03e-01	5.00e-01
**PFHpS**	1.34e-01	2.01e-01	9.08e-03	8.99e-01	1.96e-01	1.86e-01
**PFOA**	6.17e-02	3.94e-01	-4.87e-02	3.26e-01	1.17e-01	2.56e-01
**PFOS**	4.71e-02	6.30e-01	-1.46e-04	9.98e-01	7.07e-02	6.10e-01
**FOSA**	6.62e-02	5.20e-01	-1.17e-02	8.71e-01	1.05e-01	4.63e-01
**N-MeFOSAA**	-5.25e-02	6.71e-01	4.63e-02	5.96e-01	-1.02e-01	5.53e-01
**N-EtFOSAA**	-1.05e-01	4.15e-01	-7.06e-02	4.40e-01	-1.23e-01	4.93e-01
**PFNA**	1.52e-03	9.88e-01	-2.84e-02	6.71e-01	1.65e-02	9.05e-01
**PFDA**	-4.09e-02	6.80e-01	-4.24e-02	5.33e-01	-4.01e-02	7.75e-01
**PFDS**	-2.66e-01	7.65e-02	-4.20e-02	6.93e-01	-3.78e-01	6.94e-02
**PFUnA**	2.12e-02	8.45e-01	5.44e-02	4.65e-01	4.64e-03	9.76e-01
**PFDoA**	-6.50e-02	5.27e-01	7.07e-02	3.27e-01	-1.33e-01	3.55e-01
**PFTrDA**	-1.74e-01	3.99e-01	2.70e-02	8.53e-01	-2.75e-01	3.38e-01
**8:2 FTS**	3.39e-02	8.58e-01	-1.35e-01	3.11e-01	1.18e-01	6.56e-01
**4-OH-CB187**	2.83e-01	4.91e-02	2.17e-01	2.82e-02	3.16e-01	1.20e-01
**3-OH-CB153**	5.18e-01	3.14e-02	3.40e-01	4.45e-02	6.06e-01	7.03e-02
**4-OH-CB107**	-1.04e-01	5.97e-01	-3.64e-02	7.96e-01	-1.38e-01	6.13e-01

The fully adjusted model was adjusted for age, sex, BMI at sample collection, BMI at the age of 20, the year the blood sample was taken, years of regular smoking, years of irregular smoking, years of passive smoking from indoors, type of undergoing treatment, and number of childbirths.

Supplementary Table S4. Coefficients for mixed-linear regression model used for the paired comparison of per- and polyfluorinated substances (PFAS) and hydroxyl polychlorinated biphenyls (OH-PCBs) in multiple sclerosis (MS) patients (n = 907) and matched control (HC) subjects (n = 907). The coefficients and the two-way ANOVA *P* value for respective covariates are presented in the table below.

	**Sample collection year**		**Age**		**Sex (ref. male)**		**BMI at sample collection**		**BMI at the age of 20**		**Regular smoking (years)**		**Irregular smoking (years)**		**Passive smoking indoors (years)**		**Treatment (ref. no treatment)**				**Number of childbirths**		**Disease type * Sex**
	**β**	***P***	**β**	***P***	**β**	***P***	**β**	***P***	**β**	***P***	**β**	***P***	**β**	***P***	**β**	***P***	**β (First line DMT)**	**β (Other drugs)**	**β (Second linde DMT)**	***P***	**β**	***P***	***P***
**PFBS**	4.12e-02	6.15e-01	-3.45e-02	1.10e-01	1.26e-01	4.86e-01	-8.16e-03	8.76e-01	5.79e-02	3.87e-01	1.58e-02	4.91e-01	3.55e-01	2.09e-02	1.09e-02	7.97e-01	4.35e-01	4.21	-1.61	3.60e-01	-6.81e-02	7.43e-01	5.76e-01
**PFPeS**	-1.76e-01	8.50e-03	3.92e-02	2.64e-02	-7.97e-01	2.46e-01	-4.48e-02	2.88e-01	-3.92e-02	4.67e-01	1.69e-02	3.59e-01	6.38e-02	6.08e-01	-4.06e-02	2.31e-01	4.49e-01	3.31	-2.68e-01	5.32e-01	1.00e-01	5.49e-01	1.23e-01
**PFHpA**	-1.62e-01	2.14e-05	4.54e-02	8.44e-06	-5.98e-02	6.04e-01	-5.75e-02	3.21e-02	6.54e-02	5.69e-02	-3.53e-03	7.65e-01	-9.50e-02	2.18e-01	-2.00e-02	3.52e-01	-6.72e-01	3.15e-01	-7.68e-01	4.88e-01	-2.21e-02	8.32e-01	1.32e-01
**PFHxS**	-6.86e-02	4.54e-04	3.35e-02	5.42e-11	-9.98e-01	8.33e-10	-1.62e-02	1.40e-01	-4.41e-03	7.52e-01	7.48e-03	1.16e-01	6.70e-03	8.42e-01	-1.34e-02	1.29e-01	1.81e-01	9.83e-02	-9.50e-02	8.50e-01	-6.98e-02	1.25e-01	2.89e-02
**PFHpS**	-8.80e-02	8.98e-06	3.70e-02	6.75e-13	-1.01	3.33e-10	4.42e-03	6.81e-01	-1.05e-02	4.43e-01	-9.98e-04	8.30e-01	1.56e-02	6.40e-01	-6.36e-03	4.63e-01	1.60e-01	-8.60e-01	-3.20e-01	2.81e-01	-1.05e-01	2.07e-02	2.12e-01
**PFOA**	-7.78e-02	2.57e-07	2.88e-02	1.75e-13	-4.63e-01	2.33e-04	-3.64e-03	6.32e-01	-1.57e-02	1.05e-01	7.40e-04	8.21e-01	-2.36e-02	3.30e-01	-4.95e-03	4.18e-01	1.66e-01	-3.79e-03	-5.03e-02	7.39e-01	-6.23e-02	5.80e-02	1.44e-01
**PFOS**	-9.41e-02	1.21e-06	3.39e-02	1.60e-11	-6.35e-01	8.97e-05	-2.71e-02	7.65e-03	-1.08e-02	4.01e-01	-7.36e-03	9.21e-02	2.85e-02	3.71e-01	-2.33e-02	4.31e-03	-4.08e-02	-1.10	-1.92e-01	2.19e-01	-5.57e-02	1.97e-01	3.07e-01
**FOSA**	-1.41e-01	3.65e-23	3.33e-02	1.78e-18	-6.47e-02	8.59e-01	-4.87e-02	5.15e-07	1.35e-02	2.78e-01	-1.09e-02	1.07e-02	4.87e-02	8.28e-02	-1.92e-02	1.40e-02	-2.80e-01	-7.74e-01	-5.01e-01	8.91e-02	6.08e-03	8.72e-01	8.24e-01
**N-MeFOSAA**	-2.33e-01	1.32e-43	2.52e-02	1.77e-08	-8.56e-02	4.18e-01	-3.40e-02	3.36e-03	2.65e-02	7.36e-02	-1.03e-02	4.36e-02	-3.32e-02	3.21e-01	-1.13e-03	9.04e-01	-2.37e-02	-1.11	-6.07e-01	1.28e-01	1.89e-02	6.75e-01	9.84e-01
**N-EtFOSAA**	-1.86e-01	1.23e-27	1.82e-02	6.91e-05	-9.75e-02	3.75e-01	-5.26e-03	6.61e-01	-9.52e-03	5.35e-01	-4.79e-03	3.63e-01	-6.38e-02	6.43e-02	-1.64e-03	8.65e-01	-3.02e-01	-6.17e-01	8.34e-02	5.05e-01	2.09e-02	6.52e-01	9.74e-01
**PFNA**	-3.23e-02	7.29e-02	2.80e-02	2.32e-09	-2.59e-01	1.02e-01	-2.18e-02	2.93e-02	-8.76e-03	4.90e-01	-3.71e-03	3.91e-01	3.57e-02	2.45e-01	-5.71e-03	4.77e-01	-1.17e-01	-6.50e-01	-3.62e-01	3.75e-01	-5.38e-02	1.96e-01	3.23e-01
**PFDA**	-8.68e-03	6.25e-01	2.84e-02	9.73e-10	-2.66e-01	1.41e-01	-4.09e-02	4.88e-05	-1.21e-02	3.46e-01	-6.81e-03	1.19e-01	3.67e-02	2.33e-01	-4.21e-03	6.03e-01	-1.74e-01	-1.62e-01	-1.97e-01	7.52e-01	-1.89e-02	6.50e-01	5.51e-01
**PFDS**	-1.10e-01	2.40e-08	1.53e-02	3.73e-03	-3.33e-01	7.18e-01	-5.54e-02	6.87e-05	2.85e-02	1.10e-01	-3.15e-03	6.06e-01	-1.64e-02	6.82e-01	2.24e-03	8.41e-01	-3.69e-01	-1.01	5.08e-01	1.90e-01	6.68e-02	2.15e-01	2.27e-02
**PFUnA**	2.54e-02	2.16e-01	3.58e-02	2.11e-11	1.36e-01	1.10e-01	-5.94e-02	1.07e-07	1.15e-02	4.17e-01	-6.59e-03	1.72e-01	8.11e-02	1.92e-02	-7.03e-03	4.34e-01	-3.68e-01	-1.04	-3.42e-01	9.81e-02	-5.32e-02	2.56e-01	3.18e-01
**PFDoA**	-1.52e-02	2.96e-01	3.27e-02	2.85e-17	1.38e-01	1.55e-01	-6.72e-02	6.39e-12	6.92e-03	5.80e-01	-1.39e-02	1.17e-03	5.63e-02	4.69e-02	-1.55e-02	4.84e-02	-2.24e-01	-1.03	-6.55e-01	2.67e-02	-3.04e-02	4.27e-01	9.84e-01
**PFTrDA**	-4.40e-02	1.10e-01	6.07e-02	1.32e-16	4.19e-01	2.57e-02	-1.09e-01	1.28e-08	3.61e-02	1.42e-01	-2.88e-02	6.37e-04	1.34e-01	1.54e-02	-2.37e-02	1.24e-01	-3.14e-01	-2.14	-2.21e-01	2.26e-01	-3.71e-02	6.19e-01	7.87e-01
**8:2 FTS**	-2.45e-01	2.07e-19	1.67e-03	8.17e-01	5.13e-01	1.65e-02	-4.78e-02	8.37e-03	2.94e-02	2.03e-01	-8.59e-03	2.79e-01	1.51e-02	7.74e-01	8.49e-03	5.60e-01	-3.76e-02	-9.90e-01	-3.41e-01	7.32e-01	-7.89e-02	2.66e-01	6.51e-01
**4-OH-CB187**	-1.23e-01	1.42e-06	4.72e-02	1.18e-12	-4.26e-01	4.49e-02	-4.78e-02	1.04e-03	1.80e-02	3.30e-01	-3.62e-03	5.67e-01	5.08e-02	2.52e-01	-4.94e-03	6.73e-01	-4.27e-02	4.34e-02	-1.30	2.93e-02	4.87e-02	4.16e-01	4.04e-01
**3-OH-CB153**	-2.02e-01	1.34e-09	1.34e-01	1.35e-51	1.81e-02	9.27e-01	-5.18e-02	2.24e-02	1.00e-01	5.39e-04	-1.39e-02	1.61e-01	1.66e-02	8.00e-01	-2.07e-02	2.57e-01	-3.53e-01	4.51e-01	-1.03	4.05e-01	-8.24e-02	3.50e-01	1.51e-01
**4-OH-CB107**	-1.64e-01	8.98e-11	9.06e-02	9.15e-41	7.44e-02	8.54e-01	-7.62e-02	2.58e-05	1.03e-01	9.43e-06	-9.51e-04	9.05e-01	2.09e-02	6.87e-01	1.15e-02	4.28e-01	2.28e-02	-2.55e-02	-9.13e-01	4.25e-01	2.28e-02	7.44e-01	3.90e-01

β beta (regression coefficient)

Supplementary Table S5. ANOVA F values and degrees of freedom for mixed-linear regression model used for the paired comparison of per- and polyfluorinated substances (PFAS) and hydroxyl polychlorinated biphenyls (OH-PCBs) in multiple sclerosis (MS) patients (n = 907) and matched control (HC) subjects (n = 907). The two-way ANOVA *F* value and degrees of freedom (Df) for respective covariates are presented in the table below.

	Sample collection year		Age		Sex (ref. male)		BMI at sample collection		BMI at the age of 20		Regular smoking (years)		Irregular smoking (years)		Passive smoking indoors (years)		Treatment (ref. no treatment)		Number of childbirth		Disease type * Sex	
	F	Df	F	Df	F	Df	F	Df	F	Df	F	Df	F	Df	F	Df	F	Df	F	Df	F	Df
**PFBS**	2.53e-01	1	2.56	1	4.84e-01	1	2.41e-02	1	7.45e-01	1	4.72e-01	1	5.33	1	6.62e-02	1	1.07	3	1.07e-01	1	6.60e-01	3
**PFPeS**	6.92	1	4.92	1	1.35	1	1.12	1	5.28e-01	1	8.39e-01	1	2.62e-01	1	1.43	1	7.30e-01	3	3.57e-01	1	1.92	3
**PFHpA**	1.80e+01	1	1.98e+01	1	2.68e-01	1	4.58	1	3.61	1	8.93e-02	1	1.51	1	8.62e-01	1	8.08e-01	3	4.51e-02	1	1.87	3
**PFHxS**	1.23e+01	1	4.30e+01	1	3.76e+01	1	2.17	1	9.93e-02	1	2.46	1	3.96e-02	1	2.29	1	2.65e-01	3	2.35	1	3.00	3
**PFHpS**	1.97e+01	1	5.16e+01	1	3.94e+01	1	1.68e-01	1	5.87e-01	1	4.59e-02	1	2.18e-01	1	5.37e-01	1	1.27	3	5.34	1	1.50	3
**PFOA**	2.65e+01	1	5.42e+01	1	1.35e+01	1	2.28e-01	1	2.63	1	5.12e-02	1	9.47e-01	1	6.53e-01	1	4.19e-01	3	3.58	1	1.80	3
**PFOS**	2.35e+01	1	4.54e+01	1	1.53e+01	1	7.09	1	7.02e-01	1	2.83	1	7.97e-01	1	8.12	1	1.47	3	1.66	1	1.20	3
**FOSA**	9.82e+01	1	7.68e+01	1	3.18e-02	1	2.51e+01	1	1.17	1	6.49	1	3.00	1	6.02	1	2.16	3	2.58e-02	1	3.01e-01	3
**N-MeFOSAA**	1.92e+02	1	3.17e+01	1	6.56e-01	1	8.57	1	3.19	1	4.05	1	9.85e-01	1	1.46e-02	1	1.89	3	1.76e-01	1	5.20e-02	3
**N-EtFOSAA**	1.19e+02	1	1.58e+01	1	7.86e-01	1	1.91e-01	1	3.83e-01	1	8.23e-01	1	3.42	1	2.89e-02	1	7.77e-01	3	2.03e-01	1	7.36e-02	3
**PFNA**	3.21	1	3.57e+01	1	2.67	1	4.73	1	4.74e-01	1	7.34e-01	1	1.35	1	5.03e-01	1	1.03	3	1.67	1	1.16	3
**PFDA**	2.38e-01	1	3.73e+01	1	2.16	1	1.64e+01	1	8.83e-01	1	2.43	1	1.42	1	2.70e-01	1	4.00e-01	3	2.06e-01	1	7.00e-01	3
**PFDS**	3.11e+01	1	8.40	1	1.30e-01	1	1.58e+01	1	2.55	1	2.65e-01	1	1.68e-01	1	3.99e-02	1	1.58	3	1.54	1	3.18	3
**PFUnA**	1.53	1	4.48e+01	1	2.55	1	2.81e+01	1	6.56e-01	1	1.86	1	5.47	1	6.09e-01	1	2.09	3	1.29	1	1.17	3
**PFDoA**	1.09	1	7.14e+01	1	2.02	1	4.70e+01	1	3.06e-01	1	1.05e+01	1	3.94	1	3.88	1	3.05	3	6.31e-01	1	5.34e-02	3
**PFTrDA**	2.55	1	6.83e+01	1	4.97	1	3.23e+01	1	2.14	1	1.16e+01	1	5.86	1	2.35	1	1.44	3	2.47e-01	1	3.52e-01	3
**8:2 FTS**	8.11e+01	1	5.33e-02	1	5.74	1	6.93	1	1.61	1	1.17	1	8.25e-02	1	3.39e-01	1	4.27e-01	3	1.24	1	5.43e-01	3
**4-OH-CB187**	2.32e+01	1	5.05e+01	1	4.02	1	1.07e+01	1	9.45e-01	1	3.27e-01	1	1.31	1	1.77e-01	1	2.99	3	6.60e-01	1	9.71e-01	3
**3-OH-CB153**	3.67e+01	1	2.28e+02	1	8.36e-03	1	5.20	1	1.19e+01	1	1.96	1	6.38e-02	1	1.28	1	9.68e-01	3	8.71e-01	1	1.76	3
**4-OH-CB107**	4.20e+01	1	1.78e+02	1	3.35e-02	1	1.76e+01	1	1.96e+01	1	1.42e-02	1	1.62e-01	1	6.27e-01	1	9.26e-01	3	1.07e-01	1	9.99e-01	3

Supplementary Table S6. Paired comparison of per- and polyfluorinated substances (PFAS) and hydroxyl polychlorinated biphenyls (OH-PCBs) in multiple sclerosis (MS) patients and matched control (HC) subjects divided in males and females. Comparisons have been made between the entire MS group (n = 907) and HC subjects (n = 907), between patients with relapsing-remitting MS (RRMS) (n = 801) and HC subjects (n = 801), and between progressive MS (PMS) (n = 106) and HC subjects (n = 106). Log_2 fold changes (FC) were calculated using estimated marginal means based on a linear mixed-effect regression model and *P* values for the two-sided tests were computed using Satterthwaite's degrees of freedom method. No adjustments were made for multiple comparisons.

**Compound**	**Males**						**Females**					
	**MS vs HC**		**RRMS vs HC**		**PMS vs HC**		**MS vs HC**		**RRMS vs HC**		**PMS vs HC**	
	**Log_2 FC**	***P* value**	**Log_2 FC**	***P* value**	**Log_2 FC**	***P* value**	**Log_2 FC**	***P* value**	**Log_2 FC**	***P* value**	**Log_2 FC**	***P* value**
**PFBS**	-7.47e-01	3.52e-01	-2.66e-01	6.62e-01	-9.88e-01	3.76e-01	6.96e-01	3.16e-01	1.87e-01	6.57e-01	9.51e-01	3.38e-01
**PFPeS**	-1.75	6.35e-03	-3.94e-01	4.15e-01	-2.43	6.64e-03	1.31e-01	8.14e-01	-3.58e-01	2.86e-01	3.75e-01	6.37e-01
**PFHpA**	-1.41	1.13e-03	-3.64e-01	2.75e-01	-1.94	1.12e-03	-1.72e-01	6.38e-01	-1.37e-01	5.48e-01	-1.90e-01	7.14e-01
**PFHxS**	-2.59e-01	1.01e-01	-2.24e-01	5.90e-02	-2.77e-01	2.13e-01	3.00e-01	3.30e-02	-6.50e-02	4.34e-01	4.82e-01	1.72e-02
**PFHpS**	-5.45e-02	7.22e-01	-7.62e-02	5.06e-01	-4.36e-02	8.40e-01	3.22e-01	1.89e-02	9.43e-02	2.42e-01	4.36e-01	2.74e-02
**PFOA**	-6.55e-02	5.34e-01	-1.03e-01	1.90e-01	-4.66e-02	7.55e-01	1.89e-01	4.79e-02	6.01e-03	9.14e-01	2.81e-01	4.21e-02
**PFOS**	-1.01e-01	4.76e-01	-7.76e-02	4.67e-01	-1.13e-01	5.74e-01	1.96e-01	1.27e-01	7.73e-02	3.05e-01	2.55e-01	1.69e-01
**FOSA**	2.68e-02	8.62e-01	-6.42e-02	5.84e-01	7.23e-02	7.33e-01	1.06e-01	4.20e-01	4.08e-02	6.12e-01	1.38e-01	4.57e-01
**N-MeFOSAA**	-4.23e-02	8.19e-01	7.19e-02	6.12e-01	-9.94e-02	6.96e-01	-6.28e-02	6.89e-01	2.07e-02	8.31e-01	-1.05e-01	6.39e-01
**N-EtFOSAA**	-6.26e-02	7.46e-01	-5.37e-02	7.18e-01	-6.71e-02	8.00e-01	-1.48e-01	3.65e-01	-8.75e-02	3.89e-01	-1.78e-01	4.40e-01
**PFNA**	-7.04e-02	6.23e-01	-6.92e-02	5.19e-01	-7.10e-02	7.25e-01	7.34e-02	5.65e-01	1.23e-02	8.70e-01	1.04e-01	5.71e-01
**PFDA**	-8.26e-02	5.69e-01	-1.36e-01	2.14e-01	-5.62e-02	7.84e-01	8.55e-04	9.95e-01	5.07e-02	5.07e-01	-2.41e-02	8.97e-01
**PFDS**	-5.12e-01	2.29e-02	-3.55e-01	4.08e-02	-5.91e-01	5.53e-02	-1.99e-02	9.17e-01	2.71e-01	2.19e-02	-1.65e-01	5.38e-01
**PFUnA**	-7.97e-02	6.16e-01	-6.96e-02	5.59e-01	-8.47e-02	7.06e-01	1.22e-01	3.90e-01	1.78e-01	3.35e-02	9.40e-02	6.46e-01
**PFDoA**	-8.58e-02	5.76e-01	4.56e-02	6.96e-01	-1.52e-01	4.75e-01	-4.43e-02	7.36e-01	9.58e-02	2.33e-01	-1.14e-01	5.40e-01
**PFTrDA**	-3.68e-01	2.34e-01	-2.44e-03	9.92e-01	-5.51e-01	1.94e-01	1.93e-02	9.41e-01	5.65e-02	7.27e-01	6.20e-04	9.99e-01
**8:2 FTS**	1.94e-01	4.92e-01	-1.66e-01	4.41e-01	3.75e-01	3.39e-01	-1.27e-01	6.02e-01	-1.03e-01	4.86e-01	-1.38e-01	6.89e-01
**4-OH-CB187**	5.98e-02	7.77e-01	2.14e-01	1.78e-01	-1.72e-02	9.54e-01	5.07e-01	6.94e-03	2.21e-01	4.67e-02	6.50e-01	1.60e-02
**3-OH-CB153**	2.86e-01	4.26e-01	3.94e-01	1.52e-01	2.32e-01	6.39e-01	7.49e-01	1.43e-02	2.86e-01	1.28e-01	9.81e-01	2.39e-02
**4-OH-CB107**	-1.13e-01	7.03e-01	1.25e-01	5.85e-01	-2.32e-01	5.66e-01	-9.55e-02	7.01e-01	-1.98e-01	2.03e-01	-4.42e-02	9.00e-01

The fully adjusted model was adjusted for age, sex, BMI at sample collection, BMI at the age of 20, the year the blood sample was taken, years of regular smoking, years of irregular smoking, years of passive smoking (indoors), type of undergoing treatment, and number of childbirths.

Supplementary Table S7. Coefficients for mixed-linear regression model used in the sensitivity analysis of the paired comparison of per- and polyfluorinated substances (PFAS) and hydroxyl polychlorinated biphenyls (OH-PCBs) in multiple sclerosis (MS) patients (n = 907) and matched control (HC) subjects (n = 907), considering whether they were born in Swedish or not. The coefficients and the two-way ANOVA *P* value for respective covariates are presented in the table below.

	Sample collection year		Age		Sex (ref. male)		BMI at sample collection		BMI at the age of 20		Regular smoking (years)		Irregular smoking (years)		Passive smoking indoors (years)		Treatment (ref. no treatment)				Number of childbirths		Born in Sweden (ref = 0)		Disease type * Sex
	β	*P*	β	*P*	β	*P*	β	*P*	β	*P*	β	*P*	β	*P*	β	*P*	β (First line DMT)	β (Other drugs)	β (Second linde DMT)	*P*	β	*P*	β	*P*	*P*
**PFBS**	3.30e-02	6.64e-01	-4.04e-02	3.22e-02	5.62e-01	1.59e-01	-1.44e-02	7.41e-01	6.84e-03	9.01e-01	1.21e-02	5.50e-01	3.54e-01	1.80e-02	-3.56e-03	9.18e-01	-5.92e-02	4.87	-2.22	1.18e-01	-1.61e-01	2.83e-01	1.35e-01	7.53e-01	6.23e-01
**PFPeS**	-1.83e-01	2.81e-03	2.92e-02	5.58e-02	2.76e-02	5.69e-01	-1.32e-02	7.08e-01	-5.32e-02	2.30e-01	1.26e-02	4.40e-01	5.47e-02	6.52e-01	-4.00e-02	1.54e-01	4.22e-01	2.55	-5.28e-01	5.84e-01	-1.81e-01	1.35e-01	-6.65e-02	8.48e-01	2.14e-01
**PFHpA**	-1.76e-01	2.33e-07	2.44e-02	5.07e-03	4.18e-01	7.47e-03	-2.98e-02	1.91e-01	3.88e-02	1.75e-01	-5.78e-03	5.85e-01	-7.77e-02	3.11e-01	-2.54e-02	1.63e-01	-1.86e-01	-1.15	-2.15e-01	8.31e-01	-1.77e-01	1.99e-02	-8.79e-02	6.94e-01	2.47e-01
**PFHxS**	-6.79e-02	2.15e-04	2.51e-02	2.00e-08	-7.61e-01	1.35e-08	-3.30e-03	7.20e-01	-3.95e-04	9.73e-01	4.80e-03	2.60e-01	1.60e-02	6.23e-01	-2.16e-03	7.68e-01	1.28e-01	2.53e-02	2.17e-01	8.08e-01	-1.40e-01	1.80e-05	4.68e-02	6.06e-01	1.83e-01
**PFHpS**	-8.90e-02	1.06e-06	2.81e-02	2.14e-10	-8.17e-01	6.46e-10	4.60e-03	5.95e-01	2.94e-03	7.89e-01	-2.76e-03	4.90e-01	2.13e-02	4.89e-01	2.37e-03	7.29e-01	1.49e-01	-7.26e-01	-2.26e-02	4.28e-01	-1.57e-01	4.21e-07	8.73e-02	3.05e-01	1.51e-01
**PFOA**	-8.05e-02	8.71e-09	1.88e-02	2.77e-08	-1.70e-01	2.74e-01	-2.47e-03	7.11e-01	-2.01e-03	8.12e-01	-1.02e-03	7.40e-01	-3.13e-03	8.95e-01	-1.41e-03	7.89e-01	1.91e-01	-1.51e-01	1.47e-01	4.54e-01	-1.50e-01	3.61e-10	-8.52e-02	1.94e-01	1.81e-01
**PFOS**	-9.86e-02	3.01e-08	2.74e-02	2.23e-10	-5.03e-01	2.25e-04	-2.42e-02	4.34e-03	1.31e-03	9.03e-01	-9.04e-03	2.10e-02	3.13e-02	2.98e-01	-1.41e-02	3.51e-02	-5.86e-03	-8.98e-01	-9.35e-02	3.60e-01	-8.71e-02	4.20e-03	2.51e-02	7.63e-01	9.03e-02
**FOSA**	-1.54e-01	8.42e-33	3.10e-02	2.86e-21	-4.05e-02	9.54e-01	-3.72e-02	6.66e-06	2.58e-04	9.80e-01	-1.24e-02	1.22e-03	5.56e-02	4.68e-02	-1.81e-02	6.11e-03	-2.81e-01	-3.92e-01	-4.37e-01	1.25e-01	-3.89e-03	8.89e-01	2.26e-02	7.81e-01	9.23e-01
**N-MeFOSAA**	-2.56e-01	5.77e-67	2.31e-02	9.27e-10	-7.37e-03	5.38e-01	-3.61e-02	2.24e-04	2.14e-02	8.07e-02	-1.24e-02	6.31e-03	-2.54e-02	4.40e-01	1.68e-03	8.30e-01	-1.69e-03	-8.91e-01	-4.69e-01	2.30e-01	-2.17e-03	9.47e-01	-2.67e-02	7.81e-01	9.02e-01
**N-EtFOSAA**	-2.00e-01	3.14e-38	1.27e-02	1.28e-03	1.76e-02	7.65e-01	-4.03e-03	6.94e-01	-1.75e-02	1.74e-01	-6.70e-03	1.60e-01	-6.14e-02	7.54e-02	3.30e-03	6.87e-01	-3.13e-01	-3.55e-01	-7.48e-02	4.64e-01	-3.25e-02	3.42e-01	1.03e-01	3.06e-01	8.22e-01
**PFNA**	-3.96e-02	1.25e-02	1.95e-02	5.09e-07	-6.50e-02	8.69e-01	-1.35e-02	1.00e-01	-7.63e-03	4.62e-01	-6.76e-03	7.53e-02	4.48e-02	1.19e-01	-1.95e-03	7.64e-01	-1.16e-01	-1.06	-2.97e-01	1.08e-01	-1.12e-01	1.15e-04	-1.10e-01	1.74e-01	3.80e-01
**PFDA**	-1.28e-02	4.40e-01	2.18e-02	8.20e-08	-1.54e-01	4.42e-01	-3.15e-02	2.99e-04	-6.10e-03	5.79e-01	-7.61e-03	5.93e-02	4.67e-02	1.24e-01	-2.89e-03	6.75e-01	-3.32e-01	-1.45e-01	-2.96e-02	3.07e-01	-4.69e-02	1.25e-01	1.23e-02	8.86e-01	5.74e-01
**PFDS**	-1.02e-01	1.84e-08	1.11e-02	1.56e-02	-1.37e-01	6.50e-01	-3.65e-02	1.97e-03	2.26e-03	8.79e-01	-4.39e-03	4.23e-01	-1.73e-02	6.63e-01	1.19e-03	9.00e-01	-3.91e-02	-9.18e-01	5.56e-01	2.89e-01	1.96e-02	6.20e-01	1.08e-01	3.49e-01	1.85e-01
**PFUnA**	1.53e-02	4.17e-01	3.29e-02	7.20e-13	1.71e-01	3.30e-02	-5.43e-02	1.27e-08	9.28e-03	4.42e-01	-8.80e-03	4.59e-02	8.24e-02	1.39e-02	-7.42e-03	3.26e-01	-4.30e-01	-1.06	-2.64e-01	3.39e-02	-5.19e-02	1.24e-01	8.02e-02	3.92e-01	5.62e-01
**PFDoA**	-2.10e-02	1.13e-01	2.75e-02	2.39e-16	2.54e-01	2.54e-03	-5.89e-02	1.84e-12	6.36e-03	5.45e-01	-1.29e-02	8.60e-04	5.84e-02	3.90e-02	-1.57e-02	1.84e-02	-1.82e-01	-9.06e-01	-3.93e-01	1.09e-01	-6.07e-02	3.10e-02	8.46e-03	9.18e-01	9.92e-01
**PFTrDA**	-5.05e-02	4.20e-02	5.90e-02	1.74e-20	3.64e-01	7.41e-03	-1.01e-01	1.27e-09	1.80e-02	3.90e-01	-2.45e-02	1.57e-03	1.35e-01	1.65e-02	-2.38e-02	7.39e-02	-3.06e-01	-2.40	1.49e-01	1.02e-01	-3.28e-02	5.55e-01	2.34e-01	1.53e-01	6.64e-01
**8:2 FTS**	-2.60e-01	6.40e-27	-5.14e-03	4.01e-01	4.23e-01	4.50e-03	-4.37e-02	4.24e-03	4.39e-02	2.21e-02	-8.04e-03	2.56e-01	7.43e-03	8.86e-01	6.87e-03	5.73e-01	4.53e-02	-1.63e-01	-1.79e-01	9.80e-01	-4.27e-02	4.07e-01	2.96e-01	4.81e-02	4.20e-01
**4-OH-CB187**	-1.15e-01	7.75e-07	4.40e-02	1.25e-14	-3.89e-01	1.31e-02	-3.26e-02	8.62e-03	-1.04e-02	5.05e-01	-1.77e-03	7.58e-01	5.94e-02	1.69e-01	-3.39e-03	7.31e-01	-3.93e-01	-6.38e-01	-1.30	4.05e-03	3.05e-02	4.82e-01	-1.31e-01	2.84e-01	9.57e-01
**3-OH-CB153**	-1.91e-01	4.04e-11	1.26e-01	3.34e-66	1.94e-01	4.44e-01	-6.83e-02	2.03e-04	6.96e-02	2.61e-03	-1.58e-02	6.46e-02	2.76e-02	6.57e-01	-1.94e-02	1.86e-01	-1.34e-01	1.60	-7.06e-01	3.14e-01	-1.35e-01	2.91e-02	1.81e-01	3.16e-01	3.48e-01
**4-OH-CB107**	-1.64e-01	9.12e-14	8.51e-02	4.04e-51	3.05e-01	2.71e-01	-7.54e-02	5.30e-07	8.17e-02	1.49e-05	1.11e-03	8.73e-01	3.22e-02	5.24e-01	-4.82e-03	6.88e-01	1.61e-01	-6.24e-01	-6.68e-01	4.48e-01	-6.66e-02	1.82e-01	-1.68e-01	2.55e-01	5.09e-01

β beta (regression coefficient)

Supplementary Table S8. ANOVA F values and degrees of freedom for mixed-linear regression model used in the sensitivity analysis of the paired comparison of per- and polyfluorinated substances (PFAS) and hydroxyl polychlorinated biphenyls (OH-PCBs) in multiple sclerosis (MS) patients (n = 907) and matched control (HC) subjects (n = 907), considering whether they were born in Swedish or not. The two-way ANOVA *F* value and degrees of freedom (Df) for respective covariates are presented in the table below.

	**Sample collection year**		**Age**		**Sex (ref. male)**		**BMI at sample collection**		**BMI at the age of 20**		**Regular smoking (years)**		**Irregular smoking (years)**		**Passive smoking indoors (years)**		**Treatment (ref. no treatment)**		**Number of childbirth**		**Born in Sweden (ref = 0)**		**Disease type * Sex**	
	**F**	**Df**	**F**	**Df**	**F**	**Df**	**F**	**Df**	**F**	**Df**	**F**	**Df**	**F**	**Df**	**F**	**Df**	**F**	**Df**	**F**	**Df**	**F**	**Df**	**F**	**Df**
**PFBS**	1.89e-01	1	4.58	1	1.99	1	1.09e-01	1	1.55e-02	1	3.56e-01	1	5.59	1	1.05e-02	1	1.95	3	1.15	1	9.91e-02	1	5.86e-01	3
**PFPeS**	8.93	1	3.66	1	3.24e-01	1	1.40e-01	1	1.43	1	5.95e-01	1	2.04e-01	1	2.03	1	6.47e-01	3	2.22	1	3.67e-02	1	1.49	3
**PFHpA**	2.67e+01	1	7.85	1	7.15	1	1.70	1	1.84	1	2.97e-01	1	1.03	1	1.94	1	2.91e-01	3	5.41	1	1.54e-01	1	1.38	3
**PFHxS**	1.37e+01	1	3.15e+01	1	3.22e+01	1	1.28e-01	1	1.15e-03	1	1.27	1	2.42e-01	1	8.72e-02	1	3.23e-01	3	1.83e+01	1	2.66e-01	1	1.61	3
**PFHpS**	2.38e+01	1	4.03e+01	1	3.82e+01	1	2.81e-01	1	7.16e-02	1	4.76e-01	1	4.79e-01	1	1.20e-01	1	9.22e-01	3	2.55e+01	1	1.05	1	1.76	3
**PFOA**	3.31e+01	1	3.08e+01	1	1.20	1	1.37e-01	1	5.66e-02	1	1.10e-01	1	1.74e-02	1	7.13e-02	1	8.71e-01	3	3.92e+01	1	1.68	1	1.62	3
**PFOS**	3.07e+01	1	4.02e+01	1	1.36e+01	1	8.12	1	1.49e-02	1	5.32	1	1.08	1	4.43	1	1.07	3	8.18	1	9.05e-02	1	2.16	3
**FOSA**	1.42e+02	1	8.96e+01	1	3.37e-03	1	2.02e+01	1	6.16e-04	1	1.04e+01	1	3.94	1	7.50	1	1.91	3	1.96e-02	1	7.72e-02	1	1.60e-01	3
**N-MeFOSAA**	2.99e+02	1	3.74e+01	1	3.79e-01	1	1.36e+01	1	3.04	1	7.44	1	5.95e-01	1	4.60e-02	1	1.43	3	4.40e-03	1	7.71e-02	1	1.92e-01	3
**N-EtFOSAA**	1.67e+02	1	1.04e+01	1	8.90e-02	1	1.54e-01	1	1.85	1	1.97	1	3.15	1	1.61e-01	1	8.53e-01	3	9.00e-01	1	1.05	1	3.04e-01	3
**PFNA**	6.24	1	2.52e+01	1	2.74e-02	1	2.70	1	5.40e-01	1	3.16	1	2.42	1	8.98e-02	1	2.02	3	1.48e+01	1	1.84	1	1.02	3
**PFDA**	5.96e-01	1	2.87e+01	1	5.90e-01	1	1.30e+01	1	3.06e-01	1	3.55	1	2.35	1	1.75e-01	1	1.20	3	2.35	1	2.05e-02	1	6.63e-01	3
**PFDS**	3.16e+01	1	5.84	1	2.05e-01	1	9.55	1	2.33e-02	1	6.41e-01	1	1.90e-01	1	1.58e-02	1	1.25	3	2.45e-01	1	8.75e-01	1	1.61	3
**PFUnA**	6.59e-01	1	5.15e+01	1	4.55	1	3.23e+01	1	5.91e-01	1	3.97	1	6.04	1	9.63e-01	1	2.89	3	2.36	1	7.30e-01	1	6.83e-01	3
**PFDoA**	2.52	1	6.72e+01	1	9.10	1	4.95e+01	1	3.66e-01	1	1.11e+01	1	4.25	1	5.54	1	2.01	3	4.64	1	1.06e-02	1	3.19e-02	3
**PFTrDA**	4.13	1	8.60e+01	1	7.16	1	3.68e+01	1	7.36e-01	1	9.96	1	5.73	1	3.18	1	2.06	3	3.48e-01	1	2.04	1	5.25e-01	3
**8:2 FTS**	1.15e+02	1	7.03e-01	1	8.06	1	8.16	1	5.22	1	1.28	1	2.06e-02	1	3.17e-01	1	6.15e-02	3	6.87e-01	1	3.90	1	9.37e-01	3
**4-OH-CB187**	2.44e+01	1	5.94e+01	1	6.15	1	6.88	1	4.44e-01	1	9.44e-02	1	1.89	1	1.18e-01	1	4.42	3	4.94e-01	1	1.15	1	1.05e-01	3
**3-OH-CB153**	4.36e+01	1	2.95e+02	1	5.84e-01	1	1.38e+01	1	9.04	1	3.41	1	1.97e-01	1	1.74	1	1.18	3	4.75	1	1.00	1	1.10	3
**4-OH-CB107**	5.55e+01	1	2.26e+02	1	1.21	1	2.51e+01	1	1.87e+01	1	2.54e-02	1	4.05e-01	1	1.61e-01	1	8.82e-01	3	1.78	1	1.29	1	7.71e-01	3

Supplementary Table S9. Paired comparison of per- and polyfluorinated substances (PFAS) and hydroxyl polychlorinated biphenyls (OH-PCBs) in multiple sclerosis (MS) patients and matched control (HC) subjects, considering whether they were born in Swedish or not. Comparisons have been made between the entire MS group (n = 907) and HC subjects (n = 907), between patients with relapsing-remitting MS (RRMS) (n = 801) and HC subjects (n = 801), and between progressive MS (PMS) (n = 106) and HC subjects (n = 106). Log_2 fold changes (FC) were calculated using estimated marginal means based on a linear mixed-effect regression model and *P* values for the two-sided tests were computed using Satterthwaite's degrees of freedom method. No adjustments were made for multiple comparisons.

**Compound**	**MS vs HC**		**RRMS vs HC**		**PMS vs HC**	
	**Log_2 FC**	***P* value**	**Log_2 FC**	***P* value**	**Log_2 FC**	***P* value**
**PFBS**	-6.51e-02	8.97e-01	-2.62e-01	4.50e-01	3.34e-02	9.63e-01
**PFPeS**	-8.54e-01	3.68e-02	-3.26e-01	2.45e-01	-1.12	5.23e-02
**PFHpA**	-7.67e-01	5.75e-03	-3.06e-01	1.18e-01	-9.98e-01	9.53e-03
**PFHxS**	-7.24e-02	4.86e-01	-1.50e-01	3.45e-02	-3.38e-02	8.18e-01
**PFHpS**	1.25e-01	1.96e-01	-1.96e-02	7.65e-01	1.97e-01	1.51e-01
**PFOA**	4.55e-02	5.41e-01	-5.09e-02	3.14e-01	9.37e-02	3.76e-01
**PFOS**	7.72e-02	4.15e-01	-3.14e-02	6.26e-01	1.31e-01	3.28e-01
**FOSA**	4.64e-02	6.40e-01	-2.61e-02	7.06e-01	8.26e-02	5.50e-01
**N-MeFOSAA**	-1.01e-01	3.95e-01	-5.17e-03	9.50e-01	-1.49e-01	3.66e-01
**N-EtFOSAA**	-6.96e-02	5.76e-01	-5.43e-02	5.35e-01	-7.72e-02	6.55e-01
**PFNA**	8.95e-03	9.23e-01	-1.75e-02	7.82e-01	2.22e-02	8.66e-01
**PFDA**	-1.91e-02	8.47e-01	-5.64e-02	4.03e-01	-4.79e-04	9.97e-01
**PFDS**	-2.89e-01	4.18e-02	-1.53e-01	1.25e-01	-3.58e-01	7.04e-02
**PFUnA**	-7.35e-03	9.46e-01	2.02e-02	7.83e-01	-2.11e-02	8.90e-01
**PFDoA**	-4.99e-02	6.17e-01	2.70e-02	6.96e-01	-8.83e-02	5.25e-01
**PFTrDA**	-1.86e-01	3.61e-01	1.99e-02	8.90e-01	-2.89e-01	3.06e-01
**8:2 FTS**	1.07e-02	9.53e-01	-1.38e-01	2.77e-01	8.49e-02	7.38e-01
**4-OH-CB187**	1.76e-01	2.13e-01	2.55e-01	8.33e-03	1.36e-01	4.95e-01
**3-OH-CB153**	3.56e-01	1.05e-01	2.52e-01	1.01e-01	4.09e-01	1.82e-01
**4-OH-CB107**	-1.54e-01	4.04e-01	-4.91e-02	7.08e-01	-2.07e-01	4.18e-01

The model was adjusted for age, sex, BMI at sample collection, BMI at the age of 20, the year the blood sample was taken, years of regular smoking, years of irregular smoking, years of passive smoking (indoors), type of undergoing treatment, number of childbirths, and Swedish born.

Supplementary Table S10. Paired comparison of per- and polyfluorinated substances (PFAS) and hydroxyl polychlorinated biphenyls (OH-PCBs) in multiple sclerosis (MS) patients and matched control (HC) subjects divided in males and females, considering whether they were born in Swedish or not. Comparisons have been made between the entire MS group (n = 907) and HC subjects (n = 907), between patients with relapsing-remitting MS (RRMS) (n = 801) and HC subjects (n = 801), and between progressive MS (PMS) (n = 106) and HC subjects (n = 106). Log_2 fold changes (FC) were calculated using estimated marginal means based on a linear mixed-effect regression model and *P* values for the two-sided tests were computed using Satterthwaite's degrees of freedom method. No adjustments were made for multiple comparisons.

Compound	Males						Females					
	MS vs HC		RRMS vs HC		PMS vs HC		MS vs HC		RRMS vs HC		PMS vs HC	
	Log_2 FC	*P* value	Log_2 FC	*P* value	Log_2 FC	*P* value	Log_2 FC	*P* value	Log_2 FC	*P* value	Log_2 FC	*P* value
**PFBS**	-6.39e-01	4.15e-01	-2.80e-01	6.38e-01	-8.19e-01	4.55e-01	5.09e-01	3.97e-01	-2.44e-01	4.64e-01	8.85e-01	3.06e-01
**PFPeS**	-1.64	9.87e-03	-3.73e-01	4.39e-01	-2.28	1.04e-02	-6.56e-02	8.93e-01	-2.80e-01	3.00e-01	4.15e-02	9.53e-01
**PFHpA**	-1.30	2.88e-03	-4.08e-01	2.26e-01	-1.74	3.41e-03	-2.38e-01	4.65e-01	-2.04e-01	2.83e-01	-2.55e-01	5.81e-01
**PFHxS**	-2.43e-01	1.31e-01	-2.34e-01	5.33e-02	-2.47e-01	2.76e-01	9.80e-02	4.32e-01	-6.49e-02	3.36e-01	1.79e-01	3.19e-01
**PFHpS**	-6.46e-02	6.66e-01	-1.06e-01	3.47e-01	-4.41e-02	8.35e-01	3.14e-01	6.96e-03	6.64e-02	2.89e-01	4.38e-01	9.39e-03
**PFOA**	-6.21e-02	5.90e-01	-1.22e-01	1.60e-01	-3.23e-02	8.43e-01	1.53e-01	8.75e-02	1.99e-02	6.80e-01	2.20e-01	9.05e-02
**PFOS**	-1.19e-01	4.18e-01	-9.58e-02	3.84e-01	-1.30e-01	5.30e-01	2.73e-01	1.66e-02	3.31e-02	5.89e-01	3.93e-01	1.73e-02
**FOSA**	2.46e-02	8.74e-01	-6.79e-02	5.68e-01	7.09e-02	7.39e-01	6.81e-02	5.60e-01	1.57e-02	8.14e-01	9.43e-02	5.71e-01
**N-MeFOSAA**	-6.52e-02	7.25e-01	5.63e-02	6.94e-01	-1.26e-01	6.20e-01	-1.36e-01	3.27e-01	-6.66e-02	4.09e-01	-1.71e-01	3.87e-01
**N-EtFOSAA**	-5.36e-02	7.83e-01	-7.58e-02	6.15e-01	-4.25e-02	8.73e-01	-8.56e-02	5.58e-01	-3.28e-02	7.00e-01	-1.12e-01	5.90e-01
**PFNA**	-3.08e-02	8.31e-01	-7.37e-02	4.98e-01	-9.41e-03	9.63e-01	4.87e-02	6.62e-01	3.87e-02	5.24e-01	5.38e-02	7.38e-01
**PFDA**	-5.49e-02	7.21e-01	-1.45e-01	2.12e-01	-1.00e-02	9.63e-01	1.66e-02	8.88e-01	3.18e-02	6.23e-01	9.07e-03	9.58e-01
**PFDS**	-4.97e-01	2.54e-02	-3.52e-01	4.00e-02	-5.69e-01	6.21e-02	-8.19e-02	6.24e-01	4.65e-02	6.30e-01	-1.46e-01	5.40e-01
**PFUnA**	-7.69e-02	6.45e-01	-7.65e-02	5.42e-01	-7.70e-02	7.43e-01	6.22e-02	6.30e-01	1.17e-01	9.51e-02	3.48e-02	8.52e-01
**PFDoA**	-7.80e-02	6.16e-01	2.36e-02	8.43e-01	-1.29e-01	5.48e-01	-2.17e-02	8.53e-01	3.05e-02	6.49e-01	-4.79e-02	7.76e-01
**PFTrDA**	-3.84e-01	2.28e-01	-2.84e-02	9.09e-01	-5.62e-01	1.97e-01	1.25e-02	9.58e-01	6.81e-02	6.25e-01	-1.53e-02	9.64e-01
**8:2 FTS**	1.6e-01	5.73e-01	-2.2e-01	3.13e-01	3.5e-01	3.72e-01	-1.39e-01	5.19e-01	-5.56e-02	6.49e-01	-1.80e-01	5.57e-01
**4-OH-CB187**	1.25e-01	5.68e-01	2.43e-01	1.43e-01	6.65e-02	8.29e-01	2.27e-01	1.80e-01	2.67e-01	3.91e-03	2.06e-01	3.98e-01
**3-OH-CB153**	2.44e-01	4.76e-01	3.51e-01	1.83e-01	1.91e-01	6.87e-01	4.69e-01	7.11e-02	1.52e-01	3.07e-01	6.27e-01	9.05e-02
**4-OH-CB107**	-6.14e-02	8.32e-01	1.18e-01	6.01e-01	-1.51e-01	7.01e-01	-2.47e-01	2.53e-01	-2.16e-01	9.00e-02	-2.63e-01	3.91e-01

The model was adjusted for age, sex, BMI at sample collection, BMI at the age of 20, the year the blood sample was taken, years of regular smoking, years of irregular smoking, years of passive smoking (indoors), type of undergoing treatment, number of childbirths, and Swedish born.

Supplementary Table S11. Coefficients for mixed-linear regression model used in the sensitivity analysis of the paired comparison of per- and polyfluorinated substances (PFAS) and hydroxyl polychlorinated biphenyls (OH-PCBs) in multiple sclerosis (MS) patients (n =907) and matched control (HC) subjects (n = 907), considering whether they were born in a Nordic country or not. The coefficients and the two-way ANOVA *P* value for respective covariates are presented in the table below.

	Sample collection year		Age		Sex (ref. male)		BMI at sample collection		BMI at the age of 20		Regular smoking (years)		Irregular smoking (years)		Passive smoking indoors (years)		Treatment (ref. no treatment)				Number of childbirths		Born in a Nordic country (ref = 0)		Disease type * Sex
	β	*P*	β	*P*	β	*P*	β	*P*	β	*P*	β	*P*	β	*P*	β	*P*	β (First line DMT)	β (Other drugs)	β (Second linde DMT)	*P*	β	*P*	β	*P*	*P*
**PFBS**	2.99e-02	6.94e-01	-4.03e-02	3.28e-02	5.60e-01	1.57e-01	-1.40e-02	7.47e-01	6.52e-03	9.05e-01	1.22e-02	5.45e-01	3.56e-01	1.74e-02	-3.78e-03	9.13e-01	-6.27e-02	4.99	-2.23	1.09e-01	-1.63e-01	2.75e-01	4.21e-01	3.81e-01	6.08e-01
**PFPeS**	-1.81e-01	3.11e-03	2.91e-02	5.64e-02	2.84e-02	5.70e-01	-1.36e-02	6.99e-01	-5.29e-02	2.33e-01	1.25e-02	4.45e-01	5.37e-02	6.57e-01	-3.99e-02	1.53e-01	4.25e-01	2.47	-5.21e-01	6.02e-01	-1.79e-01	1.39e-01	-2.61e-01	5.03e-01	2.21e-01
**PFHpA**	-1.75e-01	2.93e-07	2.44e-02	5.10e-03	4.20e-01	7.36e-03	-2.97e-02	1.91e-01	3.87e-02	1.75e-01	-5.75e-03	5.87e-01	-7.80e-02	3.09e-01	-2.51e-02	1.66e-01	-1.82e-01	-1.18	-2.09e-01	8.24e-01	-1.77e-01	1.99e-02	-1.75e-01	4.85e-01	2.52e-01
**PFHxS**	-6.82e-02	2.06e-04	2.51e-02	2.07e-08	-7.62e-01	1.27e-08	-3.54e-03	7.00e-01	-2.04e-04	9.86e-01	4.75e-03	2.65e-01	1.61e-02	6.20e-01	-2.46e-03	7.35e-01	1.27e-01	2.37e-02	2.17e-01	8.10e-01	-1.40e-01	1.93e-05	2.63e-02	7.96e-01	1.87e-01
**PFHpS**	-9.00e-02	8.27e-07	2.80e-02	2.22e-10	-8.19e-01	6.06e-10	4.36e-03	6.13e-01	3.08e-03	7.78e-01	-2.77e-03	4.87e-01	2.20e-02	4.75e-01	1.92e-03	7.78e-01	1.48e-01	-7.03e-01	-2.48e-02	4.52e-01	-1.57e-01	4.35e-07	1.15e-01	2.30e-01	1.47e-01
**PFOA**	-7.97e-02	1.23e-08	1.89e-02	2.59e-08	-1.69e-01	2.81e-01	-2.17e-03	7.44e-01	-2.23e-03	7.92e-01	-9.81e-04	7.50e-01	-3.62e-03	8.78e-01	-9.23e-04	8.60e-01	1.92e-01	-1.66e-01	1.49e-01	4.44e-01	-1.51e-01	3.00e-10	-9.05e-02	2.20e-01	1.83e-01
**PFOS**	-9.92e-02	2.53e-08	2.74e-02	2.18e-10	-5.04e-01	2.29e-04	-2.41e-02	4.43e-03	1.18e-03	9.12e-01	-8.96e-03	2.20e-02	3.18e-02	2.91e-01	-1.41e-02	3.38e-02	-6.79e-03	-8.71e-01	-9.56e-02	3.86e-01	-8.76e-02	3.95e-03	8.44e-02	3.67e-01	8.34e-02
**FOSA**	-1.54e-01	1.10e-32	3.10e-02	3.15e-21	-4.12e-02	9.42e-01	-3.74e-02	5.77e-06	4.02e-04	9.69e-01	-1.25e-02	1.17e-03	5.56e-02	4.68e-02	-1.83e-02	5.44e-03	-2.81e-01	-4.05e-01	-4.37e-01	1.22e-01	-3.20e-03	9.08e-01	-1.98e-02	8.28e-01	9.23e-01
**N-MeFOSAA**	-2.56e-01	6.50e-67	2.32e-02	8.65e-10	-6.41e-03	5.49e-01	-3.58e-02	2.44e-04	2.13e-02	8.32e-02	-1.24e-02	6.51e-03	-2.54e-02	4.40e-01	1.92e-03	8.05e-01	-9.00e-04	-8.73e-01	-4.69e-01	2.38e-01	-3.12e-03	9.24e-01	3.37e-02	7.54e-01	9.03e-01
**N-EtFOSAA**	-2.01e-01	1.38e-38	1.27e-02	1.26e-03	1.57e-02	7.72e-01	-4.23e-03	6.80e-01	-1.73e-02	1.78e-01	-6.75e-03	1.56e-01	-6.10e-02	7.70e-02	2.86e-03	7.26e-01	-3.17e-01	-3.33e-01	-8.12e-02	4.57e-01	-3.23e-02	3.44e-01	1.55e-01	1.69e-01	8.23e-01
**PFNA**	-3.88e-02	1.46e-02	1.95e-02	4.82e-07	-6.26e-02	8.86e-01	-1.31e-02	1.11e-01	-7.97e-03	4.42e-01	-6.67e-03	7.91e-02	4.43e-02	1.24e-01	-1.31e-03	8.40e-01	-1.15e-01	-1.07	-2.94e-01	1.06e-01	-1.13e-01	9.90e-05	-9.81e-02	2.80e-01	3.80e-01
**PFDA**	-1.34e-02	4.19e-01	2.18e-02	7.75e-08	-1.54e-01	4.47e-01	-3.13e-02	3.14e-04	-6.25e-03	5.69e-01	-7.52e-03	6.19e-02	4.71e-02	1.21e-01	-2.83e-03	6.81e-01	-3.32e-01	-1.16e-01	-3.22e-02	3.08e-01	-4.77e-02	1.19e-01	8.35e-02	3.85e-01	5.74e-01
**PFDS**	-1.03e-01	1.54e-08	1.11e-02	1.60e-02	-1.39e-01	6.66e-01	-3.69e-02	1.70e-03	2.62e-03	8.60e-01	-4.49e-03	4.12e-01	-1.71e-02	6.68e-01	5.63e-04	9.52e-01	-4.29e-02	-9.22e-01	5.52e-01	2.91e-01	2.08e-02	5.98e-01	7.48e-02	5.64e-01	1.86e-01
**PFUnA**	1.43e-02	4.48e-01	3.29e-02	7.22e-13	1.70e-01	3.35e-02	-5.45e-02	1.06e-08	9.39e-03	4.35e-01	-8.80e-03	4.59e-02	8.30e-02	1.32e-02	-7.79e-03	3.00e-01	-4.32e-01	-1.03	-2.67e-01	3.55e-02	-5.18e-02	1.25e-01	1.21e-01	2.52e-01	5.56e-01
**PFDoA**	-2.15e-02	1.05e-01	2.75e-02	2.17e-16	2.54e-01	2.44e-03	-5.87e-02	1.91e-12	6.27e-03	5.50e-01	-1.29e-02	8.81e-04	5.86e-02	3.84e-02	-1.57e-02	1.84e-02	-1.82e-01	-8.85e-01	-3.95e-01	1.14e-01	-6.14e-02	2.91e-02	6.88e-02	4.55e-01	9.91e-01
**PFTrDA**	-5.34e-02	3.16e-02	5.91e-02	1.55e-20	3.60e-01	7.58e-03	-1.02e-01	1.01e-09	1.83e-02	3.81e-01	-2.46e-02	1.48e-03	1.35e-01	1.58e-02	-2.48e-02	6.23e-02	-3.16e-01	-2.34	1.33e-01	1.12e-01	-3.27e-02	5.55e-01	3.81e-01	3.79e-02	6.52e-01
**8:2 FTS**	-2.63e-01	1.70e-27	-5.15e-03	4.01e-01	4.17e-01	4.85e-03	-4.45e-02	3.52e-03	4.45e-02	2.01e-02	-8.21e-03	2.47e-01	8.59e-03	8.68e-01	5.44e-03	6.54e-01	3.56e-02	-1.14e-01	-1.94e-01	9.80e-01	-4.14e-02	4.20e-01	3.80e-01	2.39e-02	4.18e-01
**4-OH-CB187**	-1.14e-01	9.88e-07	4.40e-02	1.18e-14	-3.86e-01	1.38e-02	-3.20e-02	9.73e-03	-1.09e-02	4.87e-01	-1.65e-03	7.74e-01	5.89e-02	1.73e-01	-2.61e-03	7.90e-01	-3.91e-01	-6.46e-01	-1.30	4.17e-03	2.93e-02	5.00e-01	-1.09e-01	4.27e-01	9.55e-01
**3-OH-CB153**	-1.92e-01	3.02e-11	1.26e-01	3.40e-66	1.90e-01	4.56e-01	-6.90e-02	1.71e-04	7.01e-02	2.41e-03	-1.59e-02	6.19e-02	2.81e-02	6.51e-01	-2.04e-02	1.63e-01	-1.39e-01	1.60	-7.11e-01	3.10e-01	-1.33e-01	3.10e-02	1.52e-01	4.53e-01	3.51e-01
**4-OH-CB107**	-1.63e-01	1.40e-13	8.52e-02	3.58e-51	3.10e-01	2.58e-01	-7.47e-02	6.59e-07	8.12e-02	1.69e-05	1.26e-03	8.57e-01	3.18e-02	5.29e-01	-3.87e-03	7.46e-01	1.68e-01	-6.14e-01	-6.61e-01	4.53e-01	-6.87e-02	1.69e-01	-1.10e-01	5.05e-01	5.07e-01

ß beta (regression coefficient)

Supplementary Table S12. ANOVA F values and degrees of freedom for mixed-linear regression model used in the sensitivity analysis of the paired comparison of per- and polyfluorinated substances (PFAS) and hydroxyl polychlorinated biphenyls (OH-PCBs) in multiple sclerosis (MS) patients (n = 907) and matched control (HC) subjects (n = 907), considering whether they were born in a Nordic country or not. The two-way ANOVA *F* value and degrees of freedom (Df) for respective covariates are presented in the table below.

	**Sample collection year**		**Age**		**Sex (ref. male)**		**BMI at sample collection**		**BMI at the age of 20**		**Regular smoking (years)**		**Irregular smoking (years)**		**Passive smoking indoors (years)**		**Treatment (ref. no treatment)**		**Number of childbirth**		**Born in a Nordic country (ref = 0)**		**Disease type * Sex**	
	**F**	**Df**	**F**	**Df**	**F**	**Df**	**F**	**Df**	**F**	**Df**	**F**	**Df**	**F**	**Df**	**F**	**Df**	**F**	**Df**	**F**	**Df**	**F**	**Df**	**F**	**Df**
**PFBS**	1.55e-01	1	4.55	1	2.00	1	1.04e-01	1	1.41e-02	1	3.65e-01	1	5.64	1	1.20e-02	1	2.02	3	1.19	1	7.66e-01	1	6.10e-01	3
**PFPeS**	8.74	1	3.64	1	3.22e-01	1	1.49e-01	1	1.42	1	5.82e-01	1	1.96e-01	1	2.04	1	6.18e-01	3	2.19	1	4.48e-01	1	1.46	3
**PFHpA**	2.63e+01	1	7.83	1	7.18	1	1.70	1	1.83	1	2.95e-01	1	1.03	1	1.91	1	3.01e-01	3	5.41	1	4.85e-01	1	1.36	3
**PFHxS**	1.38e+01	1	3.14e+01	1	3.24e+01	1	1.48e-01	1	3.05e-04	1	1.24	1	2.46e-01	1	1.15e-01	1	3.21e-01	3	1.82e+01	1	6.66e-02	1	1.60	3
**PFHpS**	2.43e+01	1	4.02e+01	1	3.83e+01	1	2.55e-01	1	7.91e-02	1	4.81e-01	1	5.08e-01	1	7.92e-02	1	8.75e-01	3	2.55e+01	1	1.44	1	1.79	3
**PFOA**	3.24e+01	1	3.10e+01	1	1.16	1	1.06e-01	1	6.96e-02	1	1.01e-01	1	2.34e-02	1	3.09e-02	1	8.91e-01	3	3.96e+01	1	1.50	1	1.62	3
**PFOS**	3.10e+01	1	4.03e+01	1	1.36e+01	1	8.08	1	1.22e-02	1	5.24	1	1.11	1	4.50	1	1.01	3	8.29	1	8.11e-01	1	2.22	3
**FOSA**	1.42e+02	1	8.94e+01	1	5.24e-03	1	2.05e+01	1	1.50e-03	1	1.05e+01	1	3.94	1	7.71	1	1.93	3	1.33e-02	1	4.69e-02	1	1.61e-01	3
**N-MeFOSAA**	2.98e+02	1	3.76e+01	1	3.58e-01	1	1.34e+01	1	2.99	1	7.38	1	5.95e-01	1	6.06e-02	1	1.40	3	9.10e-03	1	9.77e-02	1	1.90e-01	3
**N-EtFOSAA**	1.69e+02	1	1.04e+01	1	8.35e-02	1	1.70e-01	1	1.81	1	2.01	1	3.12	1	1.22e-01	1	8.65e-01	3	8.93e-01	1	1.88	1	3.02e-01	3
**PFNA**	5.96	1	2.53e+01	1	2.05e-02	1	2.53	1	5.90e-01	1	3.08	1	2.37	1	4.07e-02	1	2.03	3	1.51e+01	1	1.17	1	1.02	3
**PFDA**	6.53e-01	1	2.88e+01	1	5.78e-01	1	1.30e+01	1	3.23e-01	1	3.48	1	2.39	1	1.69e-01	1	1.20	3	2.43	1	7.52e-01	1	6.63e-01	3
**PFDS**	3.20e+01	1	5.80	1	1.85e-01	1	9.82	1	3.11e-02	1	6.70e-01	1	1.83e-01	1	3.58e-03	1	1.24	3	2.78e-01	1	3.31e-01	1	1.60	3
**PFUnA**	5.76e-01	1	5.14e+01	1	4.52	1	3.27e+01	1	6.07e-01	1	3.98	1	6.13	1	1.07	1	2.85	3	2.35	1	1.31	1	6.93e-01	3
**PFDoA**	2.63	1	6.74e+01	1	9.18	1	4.94e+01	1	3.56e-01	1	1.10e+01	1	4.28	1	5.54	1	1.98	3	4.75	1	5.58e-01	1	3.69e-02	3
**PFTrDA**	4.62	1	8.62e+01	1	7.12	1	3.72e+01	1	7.66e-01	1	1.01e+01	1	5.81	1	3.47	1	1.99	3	3.47e-01	1	4.30	1	5.43e-01	3
**8:2 FTS**	1.18e+02	1	7.06e-01	1	7.93	1	8.49	1	5.39	1	1.34	1	2.76e-02	1	2.00e-01	1	6.12e-02	3	6.48e-01	1	5.09	1	9.42e-01	3
**4-OH-CB187**	2.39e+01	1	5.95e+01	1	6.06	1	6.67	1	4.81e-01	1	8.23e-02	1	1.86	1	7.09e-02	1	4.40	3	4.55e-01	1	6.30e-01	1	1.08e-01	3
**3-OH-CB153**	4.41e+01	1	2.95e+02	1	5.55e-01	1	1.41e+01	1	9.19	1	3.47	1	2.04e-01	1	1.94	1	1.19	3	4.64	1	5.62e-01	1	1.09	3
**4-OH-CB107**	5.47e+01	1	2.26e+02	1	1.28	1	2.47e+01	1	1.85e+01	1	3.25e-02	1	3.95e-01	1	1.04e-01	1	8.72e-01	3	1.89	1	4.43e-01	1	7.75e-01	3

Supplementary Table S13. Paired comparison of per- and polyfluorinated substances (PFAS) and hydroxyl polychlorinated biphenyls (OH-PCBs) in multiple sclerosis (MS) patients and matched control (HC) subjects, considering whether they were born in a Nordic country or not. Comparisons have been made between the entire MS group (n = 907) and HC subjects (n = 907), between patients with relapsing-remitting MS (RRMS) (n = 801) and HC subjects (n = 801), and between progressive MS (PMS) (n = 106) and HC subjects (n = 106). Log_2 fold changes (FC) were calculated using estimated marginal means based on a linear mixed-effect regression model and *P* values for the two-sided tests were computed using Satterthwaite's degrees of freedom method. No adjustments were made for multiple comparisons.

Compound	MS vs HC		RRMS vs HC		PMS vs HC	
	**Log_2 FC**	***P* value**	**Log_2 FC**	***P* value**	**Log_2 FC**	***P* value**
**PFBS**	-8.48e-02	8.67e-01	-2.80e-01	4.20e-01	1.28e-02	9.86e-01
**PFPeS**	-8.40e-01	4.02e-02	-3.15e-01	2.62e-01	-1.10	5.56e-02
**PFHpA**	-7.6e-01	6.29e-03	-3.0e-01	1.26e-01	-9.9e-01	1.02e-02
**PFHxS**	-7.23e-02	4.87e-01	-1.49e-01	3.53e-02	-3.39e-02	8.18e-01
**PFHpS**	1.21e-01	2.11e-01	-2.24e-02	7.33e-01	1.93e-01	1.61e-01
**PFOA**	4.80e-02	5.20e-01	-4.93e-02	3.31e-01	9.66e-02	3.61e-01
**PFOS**	7.30e-02	4.41e-01	-3.49e-02	5.87e-01	1.27e-01	3.45e-01
**FOSA**	4.85e-02	6.25e-01	-2.42e-02	7.26e-01	8.48e-02	5.39e-01
**N-MeFOSAA**	-1.04e-01	3.81e-01	-7.93e-03	9.24e-01	-1.52e-01	3.56e-01
**N-EtFOSAA**	-7.49e-02	5.47e-01	-5.86e-02	5.04e-01	-8.31e-02	6.31e-01
**PFNA**	1.10e-02	9.06e-01	-1.64e-02	7.96e-01	2.46e-02	8.52e-01
**PFDA**	-2.38e-02	8.10e-01	-6.04e-02	3.72e-01	-5.52e-03	9.69e-01
**PFDS**	-2.90e-01	4.18e-02	-1.53e-01	1.25e-01	-3.58e-01	7.03e-02
**PFUnA**	-1.18e-02	9.13e-01	1.68e-02	8.18e-01	-2.61e-02	8.64e-01
**PFDoA**	-5.37e-02	5.90e-01	2.37e-02	7.32e-01	-9.25e-02	5.06e-01
**PFTrDA**	-2.00e-01	3.27e-01	8.52e-03	9.53e-01	-3.04e-01	2.82e-01
**8:2 FTS**	-6.85e-04	9.97e-01	-1.47e-01	2.47e-01	7.23e-02	7.76e-01
**4-OH-CB187**	1.78e-01	2.09e-01	2.56e-01	8.22e-03	1.39e-01	4.87e-01
**3-OH-CB153**	3.54e-01	1.08e-01	2.50e-01	1.04e-01	4.06e-01	1.86e-01
**4-OH-CB107**	-1.55e-01	4.04e-01	-4.94e-02	7.07e-01	-2.07e-01	4.18e-01

The model was adjusted for age, sex, BMI at sample collection, BMI at the age of 20, the year the blood sample was taken, years of regular smoking, years of irregular smoking, years of passive smoking (indoors), type of undergoing treatment, number of childbirths, and born in a Nordic country.

Supplementary Table S14. Paired comparison of per- and polyfluorinated substances (PFAS) and hydroxyl polychlorinated biphenyls (OH-PCBs) in multiple sclerosis (MS) patients and matched control (HC) subjects divided in males and females, considering whether they were born in in a Nordic country or not. Comparisons have been made between the entire MS group (n = 907) and HC subjects (n = 907), between patients with relapsing-remitting MS (RRMS) (n = 801) and HC subjects (n = 801), and between progressive MS (PMS) (n = 106) and HC subjects (n = 106). Log_2 fold changes (FC) were calculated using estimated marginal means based on a linear mixed-effect regression model and *P* values for the two-sided tests were computed using Satterthwaite's degrees of freedom method. No adjustments were made for multiple comparisons.

**Compound**	**Males**						**Females**					
	**MS vs HC**		**RRMS vs HC**		**PMS vs HC**		**MS vs HC**		**RRMS vs HC**		**PMS vs HC**	
	**Log_2 FC**	***P* value**	**Log_2 FC**	***P* value**	**Log_2 FC**	***P* value**	**Log_2 FC**	***P* value**	**Log_2 FC**	***P* value**	**Log_2 FC**	***P* value**
**PFBS**	-6.71e-01	3.93e-01	-3.00e-01	6.15e-01	-8.56e-01	4.34e-01	5.01e-01	4.04e-01	-2.60e-01	4.35e-01	8.82e-01	3.07e-01
**PFPeS**	-1.62	1.10e-02	-3.61e-01	4.55e-01	-2.25	1.13e-02	-6.02e-02	9.02e-01	-2.70e-01	3.19e-01	4.47e-02	9.49e-01
**PFHpA**	-1.29	3.13e-03	-4.01e-01	2.35e-01	-1.73	3.65e-03	-2.34e-01	4.73e-01	-1.99e-01	2.96e-01	-2.51e-01	5.87e-01
**PFHxS**	-2.42e-01	1.34e-01	-2.34e-01	5.37e-02	-2.45e-01	2.80e-01	9.70e-02	4.36e-01	-6.44e-02	3.41e-01	1.78e-01	3.24e-01
**PFHpS**	-6.98e-02	6.41e-01	-1.09e-01	3.33e-01	-5.03e-02	8.12e-01	3.12e-01	7.40e-03	6.39e-02	3.08e-01	4.36e-01	9.77e-03
**PFOA**	-5.95e-02	6.06e-01	-1.20e-01	1.67e-01	-2.93e-02	8.58e-01	1.55e-01	8.28e-02	2.12e-02	6.60e-01	2.23e-01	8.64e-02
**PFOS**	-1.26e-01	3.90e-01	-9.96e-02	3.65e-01	-1.39e-01	5.01e-01	2.72e-01	1.69e-02	2.98e-02	6.27e-01	3.93e-01	1.72e-02
**FOSA**	2.84e-02	8.55e-01	-6.59e-02	5.80e-01	7.55e-02	7.23e-01	6.86e-02	5.57e-01	1.75e-02	7.95e-01	9.41e-02	5.72e-01
**N-MeFOSAA**	-7.06e-02	7.03e-01	5.32e-02	7.10e-01	-1.33e-01	6.02e-01	-1.37e-01	3.25e-01	-6.91e-02	3.93e-01	-1.71e-01	3.87e-01
**N-EtFOSAA**	-6.05e-02	7.56e-01	-8.10e-02	5.91e-01	-5.03e-02	8.50e-01	-8.93e-02	5.41e-01	-3.62e-02	6.70e-01	-1.16e-01	5.77e-01
**PFNA**	-2.96e-02	8.38e-01	-7.22e-02	5.07e-01	-8.25e-03	9.68e-01	5.15e-02	6.44e-01	3.94e-02	5.17e-01	5.75e-02	7.21e-01
**PFDA**	-6.31e-02	6.81e-01	-1.49e-01	1.99e-01	-2.01e-02	9.26e-01	1.54e-02	8.96e-01	2.81e-02	6.64e-01	9.09e-03	9.58e-01
**PFDS**	-4.96e-01	2.59e-02	-3.53e-01	4.00e-02	-5.67e-01	6.33e-02	-8.38e-02	6.16e-01	4.69e-02	6.27e-01	-1.49e-01	5.31e-01
**PFUnA**	-8.32e-02	6.18e-01	-8.03e-02	5.23e-01	-8.47e-02	7.19e-01	5.96e-02	6.44e-01	1.14e-01	1.04e-01	3.25e-02	8.62e-01
**PFDoA**	-8.42e-02	5.88e-01	1.99e-02	8.68e-01	-1.36e-01	5.26e-01	-2.33e-02	8.43e-01	2.76e-02	6.81e-01	-4.87e-02	7.72e-01
**PFTrDA**	-4.02e-01	2.07e-01	-4.21e-02	8.65e-01	-5.82e-01	1.81e-01	3.03e-03	9.90e-01	5.91e-02	6.72e-01	-2.50e-02	9.41e-01
**8:2 FTS**	1.46e-01	6.06e-01	-2.31e-01	2.90e-01	3.35e-01	3.93e-01	-1.48e-01	4.92e-01	-6.27e-02	6.09e-01	-1.90e-01	5.36e-01
**4-OH-CB187**	1.26e-01	5.66e-01	2.44e-01	1.41e-01	6.69e-02	8.28e-01	2.30e-01	1.74e-01	2.68e-01	3.90e-03	2.11e-01	3.87e-01
**3-OH-CB153**	2.43e-01	4.79e-01	3.50e-01	1.86e-01	1.90e-01	6.88e-01	4.65e-01	7.35e-02	1.51e-01	3.10e-01	6.22e-01	9.33e-02
**4-OH-CB107**	-6.43e-02	8.25e-01	1.18e-01	6.01e-01	-1.56e-01	6.93e-01	-2.45e-01	2.58e-01	-2.17e-01	8.91e-02	-2.59e-01	3.99e-01

The model was adjusted for age, sex, BMI at sample collection, BMI at the age of 20, the year the blood sample was taken, years of regular smoking, years of irregular smoking, years of passive smoking (indoors), type of undergoing treatment, number of childbirths, and born in a Nordic country.

Supplementary Table S15. Coefficients for mixed-linear regression model used in the sensitivity analysis of the paired comparison of per- and polyfluorinated substances (PFAS) and hydroxyl polychlorinated biphenyls (OH-PCBs) in multiple sclerosis (MS) patients (n = 907) and matched control (HC) subjects (n = 907), with the residential area criteria removed. The coefficients and the two-way ANOVA *P* value for respective covariates are presented in the table below.

	**Sample collection year**		**Age**		**Sex (ref. male)**		**BMI at sample collection**		**BMI at the age of 20**		**Regular smoking (years)**		**Irregular smoking (years)**		**Passive smoking indoors (years)**		**Treatment (ref. no treatment)**				**Number of childbirths**		**Disease type * Sex**
	**β**	***P***	**β**	***P***	**β**	***P***	**β**	***P***	**β**	***P***	**β**	***P***	**β**	***P***	**β**	***P***	**β (First line DMT)**	**β (Other drugs)**	**β (Second linde DMT)**	***P***	**β**	***P***	***P***
**PFBS**	3.08e-02	6.46e-01	-3.90e-02	2.47e-02	6.04e-01	1.03e-01	-1.53e-02	7.41e-01	2.38e-02	6.85e-01	3.13e-02	1.49e-01	3.45e-01	2.80e-02	-3.12e-03	9.33e-01	-4.75e-01	3.80	-2.33	2.25e-01	-1.80e-01	2.45e-01	5.74e-01
**PFPeS**	-1.81e-01	8.85e-04	3.18e-02	2.37e-02	9.98e-02	3.28e-01	-2.47e-02	5.10e-01	-3.93e-02	4.07e-01	1.48e-02	4.00e-01	4.31e-02	7.34e-01	-3.26e-02	2.79e-01	5.58e-01	2.20	-7.93e-01	6.06e-01	-2.28e-01	6.88e-02	1.48e-01
**PFHpA**	-1.73e-01	1.63e-07	2.56e-02	2.59e-03	4.56e-01	4.46e-03	-3.17e-02	1.63e-01	3.74e-02	1.92e-01	-5.92e-03	5.77e-01	-7.31e-02	3.41e-01	-2.59e-02	1.56e-01	-1.41e-01	-1.30	-2.44e-01	7.95e-01	-1.96e-01	9.64e-03	2.93e-01
**PFHxS**	-6.84e-02	1.21e-05	2.84e-02	2.54e-12	-7.15e-01	6.44e-09	-1.60e-02	1.37e-01	-8.32e-06	1.00	5.89e-03	2.42e-01	1.71e-02	6.39e-01	-4.73e-03	5.84e-01	1.14e-01	1.14e-01	-1.93e-01	8.92e-01	-1.63e-01	6.13e-06	2.98e-01
**PFHpS**	-8.93e-02	7.07e-09	3.09e-02	1.09e-14	-7.91e-01	3.00e-11	-7.11e-03	5.02e-01	4.03e-03	7.63e-01	-1.57e-03	7.51e-01	1.73e-02	6.29e-01	-4.92e-03	5.63e-01	1.93e-01	-5.12e-01	-4.87e-01	3.20e-01	-1.75e-01	8.26e-07	2.95e-01
**PFOA**	-7.92e-02	3.15e-11	2.11e-02	8.19e-12	-1.56e-01	3.84e-01	-8.47e-03	3.00e-01	-3.80e-03	7.13e-01	-1.40e-03	7.14e-01	3.58e-03	8.97e-01	-4.68e-03	4.76e-01	2.58e-01	-3.02e-01	-1.68e-01	3.44e-01	-1.67e-01	1.22e-09	3.29e-01
**PFOS**	-1.04e-01	7.42e-12	3.01e-02	1.60e-14	-4.62e-01	2.13e-04	-3.32e-02	1.40e-03	2.24e-03	8.64e-01	-8.65e-03	7.43e-02	2.76e-02	4.30e-01	-2.02e-02	1.54e-02	1.34e-01	-7.34e-01	-4.31e-01	3.34e-01	-1.13e-01	1.13e-03	4.05e-01
**FOSA**	-1.54e-01	2.06e-35	3.12e-02	6.43e-23	-3.35e-02	9.21e-01	-3.79e-02	5.82e-06	-9.08e-04	9.31e-01	-1.22e-02	1.77e-03	5.36e-02	5.73e-02	-1.94e-02	3.82e-03	-2.33e-01	-5.01e-01	-4.44e-01	1.57e-01	-4.26e-03	8.78e-01	9.64e-01
**N-MeFOSAA**	-2.56e-01	2.34e-66	2.30e-02	3.78e-10	-3.54e-04	5.59e-01	-3.57e-02	2.67e-04	1.97e-02	1.11e-01	-1.26e-02	5.87e-03	-3.12e-02	3.46e-01	8.40e-04	9.15e-01	3.77e-02	-9.38e-01	-4.08e-01	2.56e-01	-5.09e-03	8.76e-01	9.07e-01
**N-EtFOSAA**	-2.00e-01	2.68e-39	1.25e-02	1.09e-03	6.46e-03	8.65e-01	-5.58e-03	5.86e-01	-1.84e-02	1.56e-01	-7.14e-03	1.36e-01	-5.83e-02	9.28e-02	1.95e-03	8.13e-01	-1.93e-01	-4.20e-01	-7.18e-02	7.30e-01	-2.97e-02	3.85e-01	7.87e-01
**PFNA**	-3.87e-02	4.61e-03	2.06e-02	5.94e-09	-5.10e-02	8.22e-01	-1.86e-02	4.84e-02	-8.06e-03	4.99e-01	-6.30e-03	1.53e-01	5.09e-02	1.11e-01	-5.87e-03	4.38e-01	-3.91e-02	-9.93e-01	-6.32e-01	7.32e-02	-1.26e-01	5.92e-05	4.74e-01
**PFDA**	-9.42e-03	5.10e-01	2.29e-02	7.42e-10	-1.29e-01	6.13e-01	-3.74e-02	1.60e-04	-6.13e-03	6.23e-01	-6.06e-03	1.89e-01	5.14e-02	1.24e-01	-6.37e-03	4.21e-01	-2.01e-01	-5.15e-01	-2.43e-01	5.38e-01	-6.31e-02	5.52e-02	7.44e-01
**PFDS**	-1.02e-01	3.06e-09	1.05e-02	1.73e-02	-1.41e-01	6.32e-01	-3.43e-02	3.72e-03	1.83e-03	9.03e-01	-4.62e-03	4.03e-01	-2.06e-02	6.06e-01	9.59e-04	9.20e-01	2.54e-03	-9.77e-01	4.31e-01	3.88e-01	1.90e-02	6.29e-01	2.22e-01
**PFUnA**	1.96e-02	2.22e-01	3.31e-02	2.60e-15	1.90e-01	7.02e-03	-5.33e-02	1.66e-06	8.58e-04	9.51e-01	-7.63e-03	1.41e-01	7.06e-02	5.97e-02	-1.69e-02	5.73e-02	-2.59e-01	-7.80e-01	-5.97e-01	1.68e-01	-7.95e-02	3.13e-02	6.75e-01
**PFDoA**	-2.07e-02	9.22e-02	2.74e-02	1.35e-17	2.57e-01	8.70e-04	-5.99e-02	2.23e-12	6.43e-03	5.48e-01	-1.23e-02	1.92e-03	5.60e-02	5.06e-02	-1.66e-02	1.45e-02	-9.88e-02	-9.05e-01	-4.44e-01	1.30e-01	-6.44e-02	2.27e-02	9.83e-01
**PFTrDA**	-5.01e-02	3.75e-02	5.89e-02	9.27e-21	3.56e-01	7.87e-03	-1.03e-01	7.16e-10	1.96e-02	3.52e-01	-2.50e-02	1.30e-03	1.34e-01	1.68e-02	-2.69e-02	4.40e-02	-2.41e-01	-2.45	1.60e-01	1.06e-01	-3.15e-02	5.70e-01	6.83e-01
**8:2 FTS**	-2.60e-01	7.94e-30	-5.28e-03	3.63e-01	4.03e-01	3.68e-03	-4.81e-02	1.96e-03	4.43e-02	2.39e-02	-9.85e-03	1.75e-01	6.70e-03	8.98e-01	-8.62e-04	9.45e-01	1.63e-01	-4.76e-01	-2.59e-01	8.47e-01	-3.65e-02	4.80e-01	4.50e-01
**4-OH-CB187**	-1.16e-01	9.07e-09	4.56e-02	3.50e-18	-3.91e-01	9.94e-03	-4.01e-02	3.78e-03	-1.12e-02	5.21e-01	-5.92e-03	3.60e-01	6.57e-02	1.61e-01	-5.09e-04	9.63e-01	-4.18e-01	-8.47e-01	-1.43	5.69e-03	1.63e-02	7.24e-01	8.29e-01
**3-OH-CB153**	-1.96e-01	4.84e-13	1.26e-01	1.71e-66	2.13e-01	3.96e-01	-6.90e-02	2.09e-04	6.94e-02	3.17e-03	-1.70e-02	5.09e-02	2.51e-02	6.89e-01	-2.09e-02	1.61e-01	-4.53e-02	1.60	-4.11e-01	4.90e-01	-1.47e-01	1.74e-02	2.79e-01
**4-OH-CB107**	-1.64e-01	5.97e-14	8.49e-02	1.45e-48	3.22e-01	2.17e-01	-7.25e-02	1.34e-06	8.09e-02	1.95e-05	2.10e-03	7.63e-01	3.16e-02	5.32e-01	-3.90e-03	7.45e-01	1.05e-01	-6.36e-01	-6.80e-01	4.68e-01	-7.32e-02	1.42e-01	5.31e-01

ß beta (regression coefficient)

Supplementary Table S16. ANOVA F values and degrees of freedom for mixed-linear regression model used in the sensitivity analysis of the paired comparison of per- and polyfluorinated substances (PFAS) and hydroxyl polychlorinated biphenyls (OH-PCBs) in multiple sclerosis (MS) patients (n = 907) and matched control (HC) subjects (n = 907), with the residential area criteria removed. The two-way ANOVA *F* value and degrees of freedom (Df) for respective covariates are presented in the table below.

	Sample collection year		Age		Sex (ref. male)		BMI at sample collection		BMI at the age of 20		Regular smoking (years)		Irregular smoking (years)		Passive smoking indoors (years)		Treatment (ref. no treatment)		Number of childbirth		Disease type * Sex	
	**F**	**Df**	**F**	**Df**	**F**	**Df**	**F**	**Df**	**F**	**Df**	**F**	**Df**	**F**	**Df**	**F**	**Df**	**F**	**Df**	**F**	**Df**	**F**	**Df**
**PFBS**	2.11e-01	1	5.06	1	2.66	1	1.09e-01	1	1.65e-01	1	2.08	1	4.84	1	7.03e-03	1	1.46	3	1.36	1	6.64e-01	3
**PFPeS**	1.11e+01	1	5.13	1	9.56e-01	1	4.34e-01	1	6.88e-01	1	7.10e-01	1	1.15e-01	1	1.17	1	6.14e-01	3	3.32	1	1.79	3
**PFHpA**	2.77e+01	1	9.11	1	8.11	1	1.95	1	1.70	1	3.11e-01	1	9.05e-01	1	2.02	1	3.41e-01	3	6.72	1	1.24	3
**PFHxS**	1.93e+01	1	4.98e+01	1	3.41e+01	1	2.22	1	3.75e-07	1	1.37	1	2.20e-01	1	3.00e-01	1	2.06e-01	3	2.06e+01	1	1.23	3
**PFHpS**	3.39e+01	1	6.10e+01	1	4.49e+01	1	4.51e-01	1	9.08e-02	1	1.01e-01	1	2.33e-01	1	3.35e-01	1	1.17	3	2.45e+01	1	1.24	3
**PFOA**	4.48e+01	1	4.75e+01	1	7.58e-01	1	1.07	1	1.35e-01	1	1.35e-01	1	1.68e-02	1	5.09e-01	1	1.11	3	3.74e+01	1	1.15	3
**PFOS**	4.77e+01	1	6.02e+01	1	1.38e+01	1	1.02e+01	1	2.92e-02	1	3.19	1	6.22e-01	1	5.89	1	1.13	3	1.06e+01	1	9.73e-01	3
**FOSA**	1.63e+02	1	1.00e+02	1	9.81e-03	1	2.07e+01	1	7.43e-03	1	9.81	1	3.62	1	8.39	1	1.74	3	2.36e-02	1	9.37e-02	3
**N-MeFOSAA**	3.28e+02	1	3.98e+01	1	3.41e-01	1	1.34e+01	1	2.55	1	7.61	1	8.90e-01	1	1.15e-02	1	1.35	3	2.45e-02	1	1.85e-01	3
**N-EtFOSAA**	1.82e+02	1	1.07e+01	1	2.89e-02	1	2.96e-01	1	2.01	1	2.22	1	2.83	1	5.60e-02	1	4.32e-01	3	7.57e-01	1	3.52e-01	3
**PFNA**	8.05	1	3.43e+01	1	5.09e-02	1	3.90	1	4.58e-01	1	2.05	1	2.55	1	6.02e-01	1	2.32	3	1.62e+01	1	8.36e-01	3
**PFDA**	4.35e-01	1	3.84e+01	1	2.56e-01	1	1.43e+01	1	2.41e-01	1	1.73	1	2.37	1	6.47e-01	1	7.23e-01	3	3.68	1	4.13e-01	3
**PFDS**	3.56e+01	1	5.68	1	2.30e-01	1	8.44	1	1.50e-02	1	7.00e-01	1	2.66e-01	1	1.02e-02	1	1.01	3	2.33e-01	1	1.47	3
**PFUnA**	1.49	1	6.39e+01	1	7.29	1	2.31e+01	1	3.75e-03	1	2.17	1	3.55	1	3.62	1	1.69	3	4.65	1	5.10e-01	3
**PFDoA**	2.84	1	7.48e+01	1	1.11e+01	1	5.01e+01	1	3.61e-01	1	9.66	1	3.83	1	6.00	1	1.89	3	5.20	1	5.45e-02	3
**PFTrDA**	4.33	1	9.00e+01	1	7.08	1	3.85e+01	1	8.67e-01	1	1.04e+01	1	5.73	1	4.06	1	2.04	3	3.23e-01	1	4.99e-01	3
**8:2 FTS**	1.34e+02	1	8.29e-01	1	8.46	1	9.62	1	5.12	1	1.84	1	1.63e-02	1	4.79e-03	1	2.69e-01	3	4.99e-01	1	8.82e-01	3
**4-OH-CB187**	3.34e+01	1	7.76e+01	1	6.66	1	8.41	1	4.13e-01	1	8.37e-01	1	1.97	1	2.11e-03	1	4.20	3	1.25e-01	1	2.95e-01	3
**3-OH-CB153**	5.32e+01	1	3.29e+02	1	7.20e-01	1	1.38e+01	1	8.74	1	3.82	1	1.60e-01	1	1.97	1	8.07e-01	3	5.67	1	1.28	3
**4-OH-CB107**	5.75e+01	1	2.31e+02	1	1.52	1	2.36e+01	1	1.84e+01	1	9.08e-02	1	3.91e-01	1	1.06e-01	1	8.47e-01	3	2.16	1	7.36e-01	3

Supplementary Table S17. Paired comparison of per- and polyfluorinated substances (PFAS) and hydroxyl polychlorinated biphenyls (OH-PCBs) in multiple sclerosis (MS) patients and matched control (HC) subjects, with the residential area criteria removed. Comparisons have been made between the entire MS group (n = 907) and HC subjects (n = 907), between patients with relapsing-remitting MS (RRMS) (n = 801) and HC subjects (n = 801), and between progressive MS (PMS) (n = 106) and HC subjects (n =106). Log_2 fold changes (FC) were calculated using estimated marginal means based on a linear mixed-effect regression model and *P* values for the two-sided tests were computed using Satterthwaite's degrees of freedom method. No adjustments were made for multiple comparisons.

Compound	MS vs HC		RRMS vs HC		PMS vs HC	
	Log_2 FC	*P* value	Log_2 FC	*P* value	Log_2 FC	*P* value
**PFBS**	-3.50e-01	5.45e-01	-8.86e-02	8.30e-01	-4.81e-01	5.46e-01
**PFPeS**	-8.75e-01	6.16e-02	-3.69e-01	2.69e-01	-1.13	8.01e-02
**PFHpA**	-7.65e-01	7.01e-03	-3.10e-01	1.25e-01	-9.92e-01	1.11e-02
**PFHxS**	-7.60e-02	5.71e-01	-1.35e-01	1.58e-01	-4.63e-02	8.02e-01
**PFHpS**	9.47e-02	4.73e-01	2.82e-03	9.76e-01	1.41e-01	4.39e-01
**PFOA**	4.00e-02	6.95e-01	-5.38e-02	4.60e-01	8.69e-02	5.36e-01
**PFOS**	2.29e-02	8.59e-01	-1.99e-02	8.29e-01	4.43e-02	8.04e-01
**FOSA**	3.60e-02	7.29e-01	-2.12e-02	7.75e-01	6.46e-02	6.52e-01
**N-MeFOSAA**	-9.68e-02	4.27e-01	2.40e-03	9.78e-01	-1.46e-01	3.83e-01
**N-EtFOSAA**	-6.14e-02	6.31e-01	-5.85e-02	5.21e-01	-6.29e-02	7.21e-01
**PFNA**	3.93e-02	7.38e-01	-1.04e-02	9.02e-01	6.41e-02	6.92e-01
**PFDA**	-9.02e-03	9.42e-01	-6.23e-02	4.78e-01	1.76e-02	9.17e-01
**PFDS**	-2.78e-01	5.94e-02	-1.55e-01	1.40e-01	-3.40e-01	9.44e-02
**PFUnA**	-2.03e-02	8.83e-01	1.71e-02	8.62e-01	-3.90e-02	8.37e-01
**PFDoA**	-3.61e-02	7.33e-01	1.81e-02	8.11e-01	-6.31e-02	6.64e-01
**PFTrDA**	-1.77e-01	3.94e-01	1.64e-02	9.12e-01	-2.73e-01	3.38e-01
**8:2 FTS**	5.44e-02	7.79e-01	-1.31e-01	3.43e-01	1.47e-01	5.81e-01
**4-OH-CB187**	8.07e-02	6.40e-01	2.67e-01	3.01e-02	-1.25e-02	9.58e-01
**3-OH-CB153**	3.45e-01	1.37e-01	2.61e-01	1.15e-01	3.87e-01	2.25e-01
**4-OH-CB107**	-1.50e-01	4.20e-01	-4.95e-02	7.10e-01	-2.01e-01	4.34e-01

The model was adjusted for age, sex, BMI at sample collection, BMI at the age of 20, the year the blood sample was taken, years of regular smoking, years of irregular smoking, years of passive smoking (indoors), type of undergoing treatment, and number of childbirths, with the paring removed thus removing the residential area criteria.

Supplementary Table S18. Paired comparison of per- and polyfluorinated substances (PFAS) and hydroxyl polychlorinated biphenyls (OH-PCBs) in multiple sclerosis (MS) patients and matched control (HC) subjects divided in males and females, with the residential area criteria removed. Comparisons have been made between the entire MS group (n = 907) and HC subjects (n = 907), between patients with relapsing-remitting MS (RRMS) (n = 801) and HC subjects (n = 801), and between progressive MS (PMS) (n = 106) and HC subjects (n = 106). Log_2 fold changes (FC) were calculated using estimated marginal means based on a linear mixed-effect regression model and *P* values for the two-sided tests were computed using Satterthwaite's degrees of freedom method. No adjustments were made for multiple comparisons.

Compound	Males						Females					
	MS vs HC		RRMS vs HC		PMS vs HC		MS vs HC		RRMS vs HC		PMS vs HC	
	Log_2 FC	*P* value	Log_2 FC	*P* value	Log_2 FC	*P* value	Log_2 FC	*P* value	Log_2 FC	*P* value	Log_2 FC	*P* value
**PFBS**	-1.03	2.56e-01	-6.58e-02	9.26e-01	-1.51	2.18e-01	3.31e-01	6.24e-01	-1.11e-01	7.81e-01	5.53e-01	5.62e-01
**PFPeS**	-1.84	1.24e-02	-4.38e-01	4.47e-01	-2.54	1.09e-02	8.76e-02	8.73e-01	-3.00e-01	3.55e-01	2.82e-01	7.15e-01
**PFHpA**	-1.27	4.33e-03	-3.94e-01	2.58e-01	-1.71	4.65e-03	-2.59e-01	4.33e-01	-2.26e-01	2.50e-01	-2.76e-01	5.55e-01
**PFHxS**	-3.20e-01	1.29e-01	-2.10e-01	2.03e-01	-3.75e-01	1.89e-01	1.68e-01	2.84e-01	-6.07e-02	5.14e-01	2.83e-01	2.02e-01
**PFHpS**	-1.48e-01	4.76e-01	-6.88e-02	6.72e-01	-1.87e-01	5.05e-01	3.37e-01	2.89e-02	7.44e-02	4.16e-01	4.69e-01	3.16e-02
**PFOA**	-1.23e-01	4.43e-01	-1.25e-01	3.17e-01	-1.21e-01	5.76e-01	2.03e-01	8.89e-02	1.78e-02	8.01e-01	2.95e-01	7.94e-02
**PFOS**	-1.78e-01	3.81e-01	-8.36e-02	5.99e-01	-2.25e-01	4.13e-01	2.24e-01	1.39e-01	4.38e-02	6.25e-01	3.13e-01	1.42e-01
**FOSA**	2.52e-02	8.77e-01	-5.24e-02	6.82e-01	6.40e-02	7.72e-01	4.68e-02	7.00e-01	9.89e-03	8.91e-01	6.52e-02	7.04e-01
**N-MeFOSAA**	-5.99e-02	7.54e-01	6.56e-02	6.61e-01	-1.23e-01	6.36e-01	-1.34e-01	3.47e-01	-6.08e-02	4.71e-01	-1.70e-01	3.97e-01
**N-EtFOSAA**	-1.53e-02	9.39e-01	-8.54e-02	5.87e-01	1.97e-02	9.42e-01	-1.07e-01	4.72e-01	-3.17e-02	7.21e-01	-1.45e-01	4.90e-01
**PFNA**	-2.44e-02	8.95e-01	-8.88e-02	5.39e-01	7.88e-03	9.75e-01	1.03e-01	4.54e-01	6.81e-02	4.03e-01	1.20e-01	5.35e-01
**PFDA**	-6.16e-02	7.50e-01	-1.56e-01	3.04e-01	-1.46e-02	9.55e-01	4.36e-02	7.62e-01	3.11e-02	7.16e-01	4.98e-02	8.06e-01
**PFDS**	-4.90e-01	3.44e-02	-3.59e-01	4.80e-02	-5.55e-01	7.68e-02	-6.65e-02	6.99e-01	4.85e-02	6.35e-01	-1.24e-01	6.10e-01
**PFUnA**	-9.18e-02	6.72e-01	-9.67e-02	5.69e-01	-8.93e-02	7.61e-01	5.11e-02	7.51e-01	1.31e-01	1.72e-01	1.13e-02	9.60e-01
**PFDoA**	-7.53e-02	6.50e-01	7.70e-03	9.53e-01	-1.17e-01	6.03e-01	3.20e-03	9.79e-01	2.84e-02	6.98e-01	-9.41e-03	9.57e-01
**PFTrDA**	-3.61e-01	2.67e-01	-2.78e-02	9.13e-01	-5.28e-01	2.31e-01	7.65e-03	9.75e-01	6.07e-02	6.73e-01	-1.89e-02	9.56e-01
**8:2 FTS**	1.89e-01	5.33e-01	-2.15e-01	3.65e-01	3.92e-01	3.42e-01	-8.05e-02	7.22e-01	-4.62e-02	7.31e-01	-9.76e-02	7.60e-01
**4-OH-CB187**	-6.76e-02	8.03e-01	2.48e-01	2.44e-01	-2.25e-01	5.40e-01	2.29e-01	2.56e-01	2.87e-01	1.67e-02	2.00e-01	4.82e-01
**3-OH-CB153**	1.94e-01	5.93e-01	3.86e-01	1.75e-01	9.82e-02	8.42e-01	4.96e-01	6.72e-02	1.35e-01	4.01e-01	6.76e-01	7.71e-02
**4-OH-CB107**	-5.87e-02	8.41e-01	1.17e-01	6.11e-01	-1.46e-01	7.12e-01	-2.42e-01	2.66e-01	-2.16e-01	9.56e-02	-2.56e-01	4.06e-01

The model was adjusted for age, sex, BMI at sample collection, BMI at the age of 20, the year the blood sample was taken, years of regular smoking, years of irregular smoking, years of passive smoking (indoors), type of undergoing treatment, and number of childbirths, with the paring removed thus removing the residential area criteria.

Supplementary Table S19. Associations between single serum per- and polyfluorinated substances (PFAS) and hydroxyl polychlorinated biphenyls (OH-PCBs), and the odds ratio of multiple sclerosis (MS) based on the Base model (n = 1,814). Analysis were performed using logistic regression**.** No adjustments were made for multiple comparisons.

Substance (ng/ml)	Males and Females		Males		Females	
	OR (95% CI)	*P*	OR (95% CI)	*P*	OR (95% CI)	*P*
**PFHxS**	0.985 (0.923 - 1.051)	0.649	0.926 (0.820 - 1.043)	0.210	1.012 (0.938 - 1.092)	0.763
**PFHpS**	1.046 (0.979 - 1.119)	0.187	0.980 (0.872 - 1.101)	0.729	1.087 (1.004 - 1.178)	0.041
**PFOA**	1.039 (0.953 - 1.134)	0.381	0.945 (0.800 - 1.115)	0.504	1.088 (0.988 - 1.200)	0.088
**PFOS**	1.011 (0.945 - 1.083)	0.744	0.946 (0.837 - 1.068)	0.372	1.045 (0.965 - 1.133)	0.280
**FOSA**	1.035 (0.954 - 1.124)	0.410	1.007 (0.860 - 1.179)	0.931	1.038 (0.947 - 1.137)	0.427
**N-MeFOSAA**	1.026 (0.960 - 1.096)	0.458	1.032 (0.907 - 1.175)	0.634	1.028 (0.954 - 1.108)	0.463
**N-EtFOSAA**	1.040 (0.974 - 1.111)	0.238	1.030 (0.902 - 1.177)	0.657	1.043 (0.969 - 1.123)	0.262
**PFNA**	0.995 (0.921 - 1.074)	0.891	0.927 (0.805 - 1.065)	0.288	1.030 (0.943 - 1.124)	0.514
**PFDA**	0.982 (0.914 - 1.056)	0.628	0.938 (0.810 - 1.084)	0.385	1.002 (0.925 - 1.085)	0.963
**PFDS**	0.996 (0.941 - 1.058)	0.891	0.882 (0.776 - 0.999)	0.050	1.033 (0.967 - 1.104)	0.335
**PFUnA**	0.994 (0.933 - 1.060)	0.866	0.934 (0.827 - 1.052)	0.263	1.020 (0.949 - 1.098)	0.592
**PFDoA**	0.953 (0.876 - 1.036)	0.256	0.938 (0.799 - 1.100)	0.435	0.970 (0.883 - 1.065)	0.519
**PFTrDA**	0.971 (0.930 - 1.014)	0.182	0.965 (0.889 - 1.047)	0.395	0.990 (0.943 - 1.039)	0.686
**8:2 FTS**	1.024 (0.979 - 1.070)	0.300	0.994 (0.910 - 1.084)	0.884	1.030 (0.980 - 1.082)	0.247
**4-OH-CB187**	1.078 (1.024 - 1.137)	0.005	1.047 (0.949 - 1.159)	0.363	1.072 (1.012 - 1.137)	0.018
**3-OH-CB153**	1.045 (1.005 - 1.086)	0.026	1.062 (0.987 - 1.145)	0.107	1.049 (1.004 - 1.096)	0.032
**4-OH-CB107**	0.988 (0.942 - 1.036)	0.616	1.002 (0.911 - 1.101)	0.971	0.988 (0.937 - 1.042)	0.651

Model adjusted for age, sex, BMI at sample collection, and BMI at age of 20. Sex was excluded as a covariate for the sex-specific models.
OR, odds ratio; *CI*, confidence interval

Supplementary Table S20. Coefficients for the logistic regression base model used for evaluating per- and polyfluorinated substances (PFAS) and hydroxyl polychlorinated biphenyls (OH-PCBs) association with odds of multiple sclerosis (MS), males and females combined (n = 1,814). The coefficients and the two-way ANOVA *P* value for respective covariates are presented in the table below.

	**Age**		**Sex**		**Current BMI**		**BMI at age 20**	
	**β**	***P***	**β**	***P***	**β**	***P***	**β**	***P***
**PFHxS**	1.41e-03	7.72e-01	7.58e-02	5.31e-01	-1.94e-02	1.84e-01	4.77e-02	1.09e-02
**PFHpS**	8.98e-05	9.85e-01	1.29e-01	2.90e-01	-1.85e-02	2.04e-01	4.73e-02	1.15e-02
**PFOA**	5.47e-04	9.10e-01	9.95e-02	4.01e-01	-1.86e-02	2.02e-01	4.76e-02	1.09e-02
**PFOS**	8.33e-04	8.65e-01	9.34e-02	4.31e-01	-1.87e-02	2.02e-01	4.76e-02	1.10e-02
**FOSA**	1.52e-04	9.76e-01	8.72e-02	4.59e-01	-1.76e-02	2.30e-01	4.77e-02	1.08e-02
**N-MeFOSAA**	6.02e-04	9.02e-01	8.80e-02	4.55e-01	-1.81e-02	2.15e-01	4.73e-02	1.15e-02
**N-EtFOSAA**	6.64e-04	8.91e-01	8.77e-02	4.57e-01	-1.87e-02	2.01e-01	4.84e-02	9.80e-03
**PFNA**	1.16e-03	8.11e-01	8.77e-02	4.57e-01	-1.92e-02	1.89e-01	4.76e-02	1.11e-02
**PFDA**	1.43e-03	7.69e-01	8.70e-02	4.60e-01	-1.98e-02	1.77e-01	4.77e-02	1.09e-02
**PFDS**	1.12e-03	8.17e-01	8.91e-02	4.49e-01	-1.92e-02	1.88e-01	4.76e-02	1.11e-02
**PFUnA**	1.22e-03	8.03e-01	8.97e-02	4.47e-01	-1.94e-02	1.87e-01	4.77e-02	1.10e-02
**PFDoA**	2.20e-03	6.54e-01	1.01e-01	3.91e-01	-2.22e-02	1.36e-01	4.80e-02	1.05e-02
**PFTrDA**	2.57e-03	6.04e-01	1.03e-01	3.86e-01	-2.24e-02	1.31e-01	4.82e-02	1.02e-02
**8:2 FTS**	1.32e-03	7.85e-01	8.10e-02	4.92e-01	-1.79e-02	2.21e-01	4.68e-02	1.25e-02
**4-OH-CB187**	-2.13e-03	6.68e-01	1.11e-01	3.48e-01	-1.56e-02	2.86e-01	4.70e-02	1.20e-02
**3-OH-CB153**	-4.44e-03	4.14e-01	8.86e-02	4.52e-01	-1.55e-02	2.89e-01	4.47e-02	1.72e-02
**4-OH-CB107**	2.11e-03	6.87e-01	8.96e-02	4.47e-01	-2.01e-02	1.73e-01	4.86e-02	9.92e-03

ß beta (regression coefficient)

Supplementary Table S21. Coefficients for the logistic regression Base model used for evaluating per- and polyfluorinated substances (PFAS) and hydroxyl polychlorinated biphenyls (OH-PCBs) association with odds of multiple sclerosis (MS), in males (n = 458). The coefficients and the two-way ANOVA *P* value for respective covariates are presented in the table below.

	Age		Current BMI		BMI at age 20	
	β	*P*	β	*P*	β	*P*
**PFHxS**	9.36e-03	3.07e-01	-1.28e-01	6.02e-04	1.07e-01	1.17e-02
**PFHpS**	8.30e-03	3.65e-01	-1.24e-01	8.86e-04	1.05e-01	1.29e-02
**PFOA**	8.54e-03	3.50e-01	-1.24e-01	8.19e-04	1.06e-01	1.29e-02
**PFOS**	9.10e-03	3.22e-01	-1.27e-01	6.89e-04	1.05e-01	1.33e-02
**FOSA**	7.61e-03	4.22e-01	-1.22e-01	1.14e-03	1.05e-01	1.31e-02
**N-MeFOSAA**	7.27e-03	4.27e-01	-1.20e-01	1.29e-03	1.04e-01	1.48e-02
**N-EtFOSAA**	7.02e-03	4.48e-01	-1.21e-01	1.13e-03	1.06e-01	1.26e-02
**PFNA**	9.09e-03	3.21e-01	-1.27e-01	6.84e-04	1.07e-01	1.23e-02
**PFDA**	9.23e-03	3.17e-01	-1.27e-01	6.97e-04	1.07e-01	1.20e-02
**PFDS**	8.33e-03	3.61e-01	-1.26e-01	7.14e-04	1.06e-01	1.29e-02
**PFUnA**	9.82e-03	2.89e-01	-1.28e-01	6.31e-04	1.06e-01	1.27e-02
**PFDoA**	9.44e-03	3.10e-01	-1.26e-01	7.28e-04	1.05e-01	1.31e-02
**PFTrDA**	9.64e-03	3.01e-01	-1.27e-01	6.98e-04	1.08e-01	1.10e-02
**8:2 FTS**	7.81e-03	3.89e-01	-1.23e-01	9.55e-04	1.06e-01	1.26e-02
**4-OH-CB187**	6.06e-03	5.14e-01	-1.19e-01	1.38e-03	1.02e-01	1.58e-02
**3-OH-CB153**	3.67e-04	9.71e-01	-1.21e-01	1.18e-03	1.03e-01	1.58e-02
**4-OH-CB107**	7.71e-03	4.33e-01	-1.22e-01	1.08e-03	1.05e-01	1.34e-02

ß beta (regression coefficient)

Supplementary Table S22. Coefficients for the logistic regression Base model used for evaluating per- and polyfluorinated substances (PFAS) and hydroxyl polychlorinated biphenyls (OH-PCBs) association with odds of multiple sclerosis (MS), in females (n = 1,356). The coefficients and the two-way ANOVA *P* value for respective covariates are presented in the table below.

	**Age**		**Current BMI**		**BMI at age 20**	
	**β**	***P***	**β**	***P***	**β**	***P***
**PFHxS**	-8.32e-04	8.80e-01	-1.65e-03	9.13e-01	3.96e-02	4.61e-02
**PFHpS**	-2.64e-03	6.34e-01	-1.67e-03	9.12e-01	3.87e-02	5.17e-02
**PFOA**	-1.94e-03	7.25e-01	-1.04e-03	9.45e-01	3.96e-02	4.62e-02
**PFOS**	-1.69e-03	7.61e-01	-3.06e-04	9.84e-01	3.92e-02	4.82e-02
**FOSA**	-1.54e-03	7.83e-01	-4.10e-04	9.78e-01	4.00e-02	4.39e-02
**N-MeFOSAA**	-1.09e-03	8.42e-01	-9.99e-04	9.47e-01	3.95e-02	4.62e-02
**N-EtFOSAA**	-8.00e-04	8.83e-01	-1.52e-03	9.20e-01	4.06e-02	4.09e-02
**PFNA**	-9.72e-04	8.59e-01	-1.32e-03	9.30e-01	3.98e-02	4.50e-02
**PFDA**	-5.93e-04	9.14e-01	-1.68e-03	9.12e-01	3.96e-02	4.58e-02
**PFDS**	-8.15e-04	8.81e-01	-6.41e-04	9.66e-01	3.98e-02	4.49e-02
**PFUnA**	-1.06e-03	8.49e-01	-8.18e-04	9.57e-01	3.95e-02	4.66e-02
**PFDoA**	1.25e-04	9.82e-01	-3.61e-03	8.14e-01	3.99e-02	4.41e-02
**PFTrDA**	-3.43e-05	9.95e-01	-2.81e-03	8.55e-01	3.97e-02	4.53e-02
**8:2 FTS**	-3.25e-04	9.52e-01	-3.31e-04	9.83e-01	3.86e-02	5.24e-02
**4-OH-CB187**	-3.71e-03	5.10e-01	6.73e-04	9.65e-01	4.06e-02	4.08e-02
**3-OH-CB153**	-6.46e-03	2.91e-01	2.26e-03	8.82e-01	3.63e-02	6.83e-02
**4-OH-CB107**	4.54e-04	9.38e-01	-2.68e-03	8.61e-01	4.07e-02	4.16e-02

β beta (regression coefficient)

Supplementary Table S23. Coefficients for the logistic regression for the fully adjusted model used for evaluating per- and polyfluorinated substances (PFAS) and hydroxyl polychlorinated biphenyls (OH-PCBs) association with odds of multiple sclerosis (MS), males and females (n = 1,814). The coefficients and the two-way ANOVA *P* value for respective covariates are presented in the table below.

	Age		Sex		Current BMI		BMI at age 20		Regular smoking		Irregular smoking		Nr. childbirths		History of infectious mononucleosis		Sun exposure			Swedish born (ref = 0)	
	**β**	***P***	**β**	***P***	**β**	***P***	**β**	***P***	**β**	***P***	**β**	***P***	**β**	***P***	**β**	***P***	**Median β**	**High β**	***P***	**β**	***P***
**PFHxS**	5.06e-03	3.83e-01	2.45e-01	1.25e-01	-1.76e-02	2.80e-01	4.57e-02	2.48e-02	7.82e-01	5.97e-05	2.55e-01	5.81e-01	-1.02e-01	6.96e-02	3.25e-01	1.75e-02	-2.36e-02	-3.77e-01	1.57e-02	2.45e-01	1.21e-01
**PFHpS**	2.58e-03	6.56e-01	2.88e-01	7.04e-02	-1.64e-02	3.11e-01	4.55e-02	2.54e-02	7.70e-01	7.56e-05	2.35e-01	6.11e-01	-8.56e-02	1.21e-01	3.25e-01	1.77e-02	-4.55e-02	-4.06e-01	1.01e-02	2.45e-01	1.21e-01
**PFOA**	3.03e-03	6.01e-01	2.60e-01	9.96e-02	-1.65e-02	3.08e-01	4.59e-02	2.39e-02	7.69e-01	7.71e-05	2.29e-01	6.21e-01	-8.66e-02	1.19e-01	3.25e-01	1.76e-02	-4.29e-02	-4.09e-01	9.66e-03	2.51e-01	1.12e-01
**PFOS**	2.89e-03	6.17e-01	2.72e-01	8.53e-02	-1.53e-02	3.48e-01	4.57e-02	2.46e-02	7.78e-01	6.29e-05	2.30e-01	6.19e-01	-9.04e-02	1.02e-01	3.25e-01	1.75e-02	-4.95e-02	-4.10e-01	9.87e-03	2.47e-01	1.18e-01
**FOSA**	2.30e-03	6.94e-01	2.59e-01	1.01e-01	-1.48e-02	3.62e-01	4.60e-02	2.37e-02	7.90e-01	5.12e-05	2.17e-01	6.39e-01	-9.53e-02	8.40e-02	3.31e-01	1.58e-02	-4.91e-02	-4.21e-01	8.01e-03	2.43e-01	1.24e-01
**N-MeFOSAA**	3.41e-03	5.52e-01	2.61e-01	9.84e-02	-1.60e-02	3.24e-01	4.53e-02	2.59e-02	7.77e-01	6.54e-05	2.52e-01	5.85e-01	-9.53e-02	8.39e-02	3.27e-01	1.69e-02	-3.68e-02	-4.08e-01	9.48e-03	2.47e-01	1.19e-01
**N-EtFOSAA**	3.80e-03	5.05e-01	2.60e-01	9.95e-02	-1.70e-02	2.94e-01	4.64e-02	2.28e-02	7.71e-01	7.34e-05	2.55e-01	5.81e-01	-9.50e-02	8.67e-02	3.28e-01	1.67e-02	-3.41e-02	-4.06e-01	9.89e-03	2.44e-01	1.23e-01
**PFNA**	3.74e-03	5.15e-01	2.59e-01	1.01e-01	-1.65e-02	3.10e-01	4.58e-02	2.43e-02	7.75e-01	6.73e-05	2.34e-01	6.12e-01	-9.34e-02	9.27e-02	3.27e-01	1.69e-02	-3.71e-02	-3.96e-01	1.18e-02	2.49e-01	1.15e-01
**PFDA**	4.49e-03	4.35e-01	2.60e-01	9.95e-02	-1.75e-02	2.82e-01	4.57e-02	2.48e-02	7.71e-01	7.37e-05	2.53e-01	5.84e-01	-9.77e-02	7.93e-02	3.26e-01	1.73e-02	-2.51e-02	-3.82e-01	1.42e-02	2.48e-01	1.17e-01
**PFDS**	4.13e-03	4.68e-01	2.60e-01	1.01e-01	-1.68e-02	3.01e-01	4.57e-02	2.48e-02	7.74e-01	6.87e-05	2.45e-01	5.96e-01	-9.65e-02	8.17e-02	3.25e-01	1.77e-02	-3.16e-02	-3.90e-01	1.28e-02	2.46e-01	1.19e-01
**PFUnA**	3.76e-03	5.17e-01	2.54e-01	1.09e-01	-1.61e-02	3.24e-01	4.55e-02	2.54e-02	7.76e-01	6.59e-05	2.36e-01	6.10e-01	-9.50e-02	8.69e-02	3.24e-01	1.79e-02	-3.51e-02	-3.92e-01	1.24e-02	2.46e-01	1.20e-01
**PFDoA**	3.72e-03	5.25e-01	2.55e-01	1.09e-01	-1.59e-02	3.35e-01	4.55e-02	2.54e-02	7.78e-01	6.52e-05	2.36e-01	6.10e-01	-9.52e-02	8.61e-02	3.24e-01	1.80e-02	-3.78e-02	-3.96e-01	1.21e-02	2.46e-01	1.19e-01
**PFTrDA**	4.52e-03	4.41e-01	2.63e-01	9.73e-02	-1.76e-02	2.85e-01	4.58e-02	2.45e-02	7.71e-01	7.56e-05	2.49e-01	5.91e-01	-9.67e-02	8.13e-02	3.25e-01	1.77e-02	-2.47e-02	-3.82e-01	1.44e-02	2.49e-01	1.16e-01
**8:2 FTS**	4.34e-03	4.45e-01	2.50e-01	1.15e-01	-1.58e-02	3.29e-01	4.47e-02	2.82e-02	7.72e-01	7.13e-05	2.32e-01	6.16e-01	-9.47e-02	8.88e-02	3.31e-01	1.58e-02	-4.38e-02	-4.17e-01	8.60e-03	2.39e-01	1.31e-01
**4-OH-CB187**	6.71e-04	9.09e-01	2.88e-01	6.93e-02	-1.30e-02	4.24e-01	4.62e-02	2.33e-02	7.83e-01	5.91e-05	1.82e-01	6.94e-01	-9.60e-02	8.31e-02	3.38e-01	1.40e-02	-5.99e-02	-4.26e-01	7.47e-03	2.52e-01	1.12e-01
**3-OH-CB153**	-2.86e-03	6.53e-01	2.55e-01	1.08e-01	-1.27e-02	4.35e-01	4.20e-02	3.93e-02	7.91e-01	4.98e-05	2.27e-01	6.24e-01	-8.87e-02	1.11e-01	3.43e-01	1.27e-02	-5.30e-02	-4.26e-01	6.98e-03	2.45e-01	1.22e-01
**4-OH-CB107**	5.20e-03	4.00e-01	2.62e-01	9.80e-02	-1.78e-02	2.76e-01	4.65e-02	2.30e-02	7.77e-01	6.53e-05	2.43e-01	5.99e-01	-9.79e-02	7.90e-02	3.21e-01	1.91e-02	-2.54e-02	-3.81e-01	1.44e-02	2.45e-01	1.21e-01

ß beta (regression coefficient)

Supplementary Table S24. Coefficients for the logistic regression for the fully adjusted model used for evaluating per- and polyfluorinated substances (PFAS) and hydroxyl polychlorinated biphenyls (OH-PCBs) association with odds of multiple sclerosis (MS), in males (n = 458). The coefficients and the two-way ANOVA *P* value for respective covariates are presented in the table below.

	Age		Current BMI		BMI at age 20		Regular smoking		Irregular smoking		History of infectious mononucleosis		Sun exposure			Swedish born (ref = 0)	
	**β**	***P***	**β**	***P***	**β**	***P***	**β**	***P***	**β**	***P***	**β**	***P***	**Median β**	**High β**	***P***	**β**	***P***
**PFHxS**	1.02e-04	9.87e-01	-8.19e-03	6.41e-01	4.28e-02	6.00e-02	6.75e-01	3.07e-03	1.22e-01	8.10e-01	4.93e-01	2.33e-03	-3.87e-02	-4.63e-01	7.31e-03	1.89e-01	2.97e-01
**PFHpS**	-2.34e-03	7.18e-01	-7.48e-03	6.70e-01	4.09e-02	7.24e-02	6.71e-01	3.29e-03	1.29e-01	7.98e-01	4.89e-01	2.59e-03	-6.82e-02	-4.95e-01	4.98e-03	1.86e-01	3.06e-01
**PFOA**	-1.82e-03	7.78e-01	-7.20e-03	6.81e-01	4.25e-02	6.17e-02	6.63e-01	3.65e-03	1.05e-01	8.35e-01	4.92e-01	2.43e-03	-6.73e-02	-5.10e-01	3.79e-03	1.98e-01	2.74e-01
**PFOS**	-1.89e-03	7.71e-01	-5.53e-03	7.53e-01	4.17e-02	6.66e-02	6.75e-01	3.05e-03	1.18e-01	8.16e-01	4.89e-01	2.59e-03	-7.59e-02	-5.01e-01	4.78e-03	1.89e-01	2.97e-01
**FOSA**	-2.57e-03	6.95e-01	-4.97e-03	7.78e-01	4.33e-02	5.75e-02	6.95e-01	2.35e-03	9.33e-02	8.54e-01	5.04e-01	1.95e-03	-6.72e-02	-5.09e-01	3.80e-03	1.80e-01	3.22e-01
**N-MeFOSAA**	-6.40e-04	9.20e-01	-7.18e-03	6.82e-01	4.27e-02	6.08e-02	6.80e-01	2.84e-03	1.42e-01	7.79e-01	4.98e-01	2.16e-03	-4.66e-02	-4.82e-01	5.52e-03	1.87e-01	3.01e-01
**N-EtFOSAA**	-6.05e-06	9.99e-01	-8.66e-03	6.21e-01	4.40e-02	5.37e-02	6.72e-01	3.19e-03	1.39e-01	7.84e-01	4.97e-01	2.17e-03	-4.48e-02	-4.89e-01	5.02e-03	1.87e-01	3.03e-01
**PFNA**	-9.24e-04	8.85e-01	-6.79e-03	6.99e-01	4.29e-02	5.93e-02	6.76e-01	3.01e-03	1.02e-01	8.41e-01	5.02e-01	1.99e-03	-6.20e-02	-4.87e-01	5.55e-03	1.95e-01	2.82e-01
**PFDA**	-4.23e-05	9.95e-01	-7.79e-03	6.58e-01	4.28e-02	6.01e-02	6.78e-01	2.92e-03	1.16e-01	8.19e-01	4.91e-01	2.46e-03	-4.20e-02	-4.66e-01	7.06e-03	1.87e-01	3.02e-01
**PFDS**	-4.48e-04	9.44e-01	-5.97e-03	7.34e-01	4.29e-02	5.96e-02	6.84e-01	2.68e-03	1.46e-01	7.73e-01	4.98e-01	2.15e-03	-6.05e-02	-4.88e-01	5.36e-03	1.78e-01	3.25e-01
**PFUnA**	-1.14e-03	8.60e-01	-5.32e-03	7.64e-01	4.17e-02	6.68e-02	6.85e-01	2.64e-03	9.94e-02	8.45e-01	4.90e-01	2.52e-03	-5.70e-02	-4.74e-01	6.79e-03	1.81e-01	3.18e-01
**PFDoA**	-7.04e-04	9.14e-01	-5.95e-03	7.40e-01	4.22e-02	6.39e-02	6.84e-01	2.72e-03	1.11e-01	8.27e-01	4.92e-01	2.40e-03	-5.44e-02	-4.80e-01	6.25e-03	1.86e-01	3.06e-01
**PFTrDA**	-2.66e-04	9.68e-01	-7.41e-03	6.77e-01	4.27e-02	6.07e-02	6.78e-01	2.93e-03	1.17e-01	8.17e-01	4.91e-01	2.45e-03	-4.51e-02	-4.69e-01	7.06e-03	1.85e-01	3.09e-01
**8:2 FTS**	4.47e-04	9.44e-01	-6.42e-03	7.15e-01	4.09e-02	7.33e-02	6.77e-01	2.95e-03	1.19e-01	8.15e-01	5.09e-01	1.74e-03	-5.87e-02	-5.08e-01	3.85e-03	1.73e-01	3.40e-01
**4-OH-CB187**	-4.13e-03	5.31e-01	-3.75e-03	8.31e-01	4.37e-02	5.54e-02	6.68e-01	3.47e-03	5.62e-02	9.12e-01	5.07e-01	1.82e-03	-7.60e-02	-5.00e-01	4.76e-03	1.78e-01	3.27e-01
**3-OH-CB153**	-6.95e-03	3.31e-01	-3.06e-03	8.63e-01	3.84e-02	9.24e-02	6.87e-01	2.60e-03	1.07e-01	8.32e-01	5.15e-01	1.56e-03	-6.58e-02	-5.00e-01	4.39e-03	1.93e-01	2.87e-01
**4-OH-CB107**	2.00e-03	7.72e-01	-9.89e-03	5.77e-01	4.45e-02	5.21e-02	6.85e-01	2.67e-03	1.25e-01	8.05e-01	4.88e-01	2.62e-03	-2.82e-02	-4.50e-01	8.47e-03	1.85e-01	3.07e-01

ß beta (regression coefficient)

Supplementary Table S25. Coefficients for the logistic regression for the fully adjusted model used for evaluating per- and polyfluorinated substances (PFAS) and hydroxyl polychlorinated biphenyls (OH-PCBs) association with odds of multiple sclerosis (MS), in females (n = 1,356). The coefficients and the two-way ANOVA *P* value for respective covariates are presented in the table below.

	Age		Current BMI		BMI at age 20		Regular smoking		Irregular smoking		Nr. childbirths		History of infectious mononucleosis		Sun exposure			Swedish born (ref = 0)	
	β	*P*	β	*P*	β	*P*	β	*P*	β	*P*	β	*P*	β	*P*	Median β	High β	*P*	β	*P*
**PFHxS**	5.12e-03	4.66e-01	-7.49e-03	6.69e-01	4.17e-02	6.71e-02	6.71e-01	3.30e-03	1.52e-01	7.64e-01	-1.04e-01	7.59e-02	4.86e-01	2.75e-03	-3.61e-02	-4.55e-01	8.65e-03	2.10e-01	2.48e-01
**PFHpS**	1.77e-03	8.02e-01	-6.91e-03	6.93e-01	4.03e-02	7.72e-02	6.65e-01	3.59e-03	1.48e-01	7.69e-01	-8.28e-02	1.47e-01	4.84e-01	2.93e-03	-6.38e-02	-4.87e-01	5.75e-03	2.04e-01	2.62e-01
**PFOA**	2.29e-03	7.46e-01	-6.73e-03	7.01e-01	4.17e-02	6.73e-02	6.59e-01	3.89e-03	1.28e-01	7.99e-01	-8.26e-02	1.51e-01	4.86e-01	2.77e-03	-6.19e-02	-4.98e-01	4.78e-03	2.14e-01	2.38e-01
**PFOS**	2.49e-03	7.24e-01	-5.12e-03	7.71e-01	4.09e-02	7.31e-02	6.68e-01	3.41e-03	1.41e-01	7.80e-01	-9.13e-02	1.10e-01	4.83e-01	2.96e-03	-7.12e-02	-4.93e-01	5.56e-03	2.08e-01	2.52e-01
**FOSA**	1.97e-03	7.80e-01	-4.18e-03	8.13e-01	4.22e-02	6.46e-02	6.88e-01	2.67e-03	1.19e-01	8.14e-01	-9.98e-02	8.03e-02	4.97e-01	2.28e-03	-6.73e-02	-5.06e-01	4.21e-03	2.01e-01	2.68e-01
**N-MeFOSAA**	3.90e-03	5.73e-01	-6.39e-03	7.16e-01	4.15e-02	6.83e-02	6.73e-01	3.21e-03	1.66e-01	7.43e-01	-1.00e-01	8.02e-02	4.91e-01	2.54e-03	-4.67e-02	-4.78e-01	6.17e-03	2.09e-01	2.51e-01
**N-EtFOSAA**	4.53e-03	5.09e-01	-7.76e-03	6.58e-01	4.28e-02	6.11e-02	6.65e-01	3.60e-03	1.63e-01	7.47e-01	-1.01e-01	8.05e-02	4.90e-01	2.55e-03	-4.54e-02	-4.84e-01	5.66e-03	2.08e-01	2.52e-01
**PFNA**	3.50e-03	6.15e-01	-6.28e-03	7.20e-01	4.18e-02	6.64e-02	6.69e-01	3.38e-03	1.28e-01	8.00e-01	-9.34e-02	1.04e-01	4.94e-01	2.39e-03	-5.78e-02	-4.80e-01	6.43e-03	2.14e-01	2.39e-01
**PFDA**	4.64e-03	5.04e-01	-7.15e-03	6.85e-01	4.17e-02	6.76e-02	6.70e-01	3.35e-03	1.44e-01	7.75e-01	-1.01e-01	7.90e-02	4.86e-01	2.83e-03	-4.05e-02	-4.60e-01	7.99e-03	2.10e-01	2.49e-01
**PFDS**	4.09e-03	5.50e-01	-5.12e-03	7.71e-01	4.18e-02	6.71e-02	6.77e-01	3.04e-03	1.70e-01	7.36e-01	-1.01e-01	7.65e-02	4.91e-01	2.53e-03	-6.11e-02	-4.85e-01	5.87e-03	2.00e-01	2.71e-01
**PFUnA**	3.40e-03	6.27e-01	-4.92e-03	7.81e-01	4.08e-02	7.36e-02	6.77e-01	3.04e-03	1.27e-01	8.02e-01	-9.67e-02	9.15e-02	4.84e-01	2.91e-03	-5.46e-02	-4.69e-01	7.50e-03	2.03e-01	2.64e-01
**PFDoA**	3.98e-03	5.72e-01	-5.64e-03	7.53e-01	4.12e-02	7.08e-02	6.75e-01	3.16e-03	1.38e-01	7.84e-01	-9.94e-02	8.37e-02	4.86e-01	2.80e-03	-5.10e-02	-4.72e-01	7.24e-03	2.08e-01	2.53e-01
**PFTrDA**	4.35e-03	5.39e-01	-6.63e-03	7.10e-01	4.16e-02	6.84e-02	6.70e-01	3.32e-03	1.43e-01	7.77e-01	-1.01e-01	7.76e-02	4.85e-01	2.86e-03	-4.49e-02	-4.65e-01	7.85e-03	2.07e-01	2.56e-01
**8:2 FTS**	4.87e-03	4.77e-01	-5.65e-03	7.48e-01	3.99e-02	8.13e-02	6.70e-01	3.33e-03	1.44e-01	7.76e-01	-9.88e-02	8.71e-02	5.02e-01	2.08e-03	-5.85e-02	-5.03e-01	4.45e-03	1.95e-01	2.84e-01
**4-OH-CB187**	3.97e-04	9.55e-01	-2.97e-03	8.66e-01	4.26e-02	6.27e-02	6.60e-01	3.93e-03	8.12e-02	8.72e-01	-9.91e-02	8.39e-02	5.01e-01	2.13e-03	-7.65e-02	-4.97e-01	5.26e-03	1.99e-01	2.74e-01
**3-OH-CB153**	-2.33e-03	7.62e-01	-2.57e-03	8.85e-01	3.77e-02	9.99e-02	6.80e-01	2.94e-03	1.32e-01	7.94e-01	-9.34e-02	1.05e-01	5.08e-01	1.86e-03	-6.51e-02	-4.95e-01	4.94e-03	2.13e-01	2.41e-01
**4-OH-CB107**	7.00e-03	3.46e-01	-9.20e-03	6.04e-01	4.36e-02	5.76e-02	6.80e-01	2.96e-03	1.52e-01	7.63e-01	-1.05e-01	7.08e-02	4.80e-01	3.15e-03	-2.78e-02	-4.46e-01	9.50e-03	2.07e-01	2.54e-01

β beta (regression coefficient)

Supplementary Table S26. Coefficients for the logistic regression for the fully adjusted model including Nordic born status used for evaluating per- and polyfluorinated substances (PFAS) and hydroxyl polychlorinated biphenyls (OH-PCBs) association with odds of multiple sclerosis (MS), males and females (n = 1,814). The coefficients and the two-way ANOVA *P* value for respective covariates are presented in the table below.

	**Age**		**Sex**		**Current BMI**		**BMI at age 20**		**Regular smoking**		**Irregular smoking**		**Nr. childbirths**		**History of infectious mononucleosis**		**Sun exposure**			**Born in a Nordic country (ref = 0)**	
	**β**	***P***	**β**	***P***	**β**	***P***	**β**	***P***	**β**	***P***	**β**	***P***	**β**	***P***	**β**	***P***	**Median β**	**High β**	***P***	**β**	***P***
**PFHxS**	4.95e-03	3.93e-01	2.45e-01	1.25e-01	-1.78e-02	2.72e-01	4.60e-02	2.38e-02	7.79e-01	6.30e-05	2.68e-01	5.62e-01	-1.03e-01	6.87e-02	3.16e-01	2.13e-02	-2.30e-02	-3.71e-01	1.81e-02	3.79e-01	3.19e-02
**PFHpS**	2.53e-03	6.63e-01	2.87e-01	7.13e-02	-1.67e-02	3.02e-01	4.57e-02	2.44e-02	7.68e-01	7.82e-05	2.49e-01	5.91e-01	-8.64e-02	1.18e-01	3.15e-01	2.16e-02	-4.45e-02	-3.99e-01	1.19e-02	3.83e-01	2.99e-02
**PFOA**	2.94e-03	6.12e-01	2.59e-01	1.01e-01	-1.68e-02	2.99e-01	4.62e-02	2.28e-02	7.67e-01	8.05e-05	2.43e-01	6.00e-01	-8.70e-02	1.18e-01	3.15e-01	2.15e-02	-4.24e-02	-4.03e-01	1.13e-02	3.91e-01	2.71e-02
**PFOS**	2.84e-03	6.24e-01	2.71e-01	8.65e-02	-1.56e-02	3.38e-01	4.60e-02	2.36e-02	7.76e-01	6.52e-05	2.44e-01	5.98e-01	-9.11e-02	9.93e-02	3.15e-01	2.13e-02	-4.85e-02	-4.03e-01	1.16e-02	3.85e-01	2.94e-02
**FOSA**	2.19e-03	7.07e-01	2.58e-01	1.03e-01	-1.50e-02	3.55e-01	4.63e-02	2.28e-02	7.89e-01	5.23e-05	2.30e-01	6.20e-01	-9.62e-02	8.13e-02	3.21e-01	1.92e-02	-4.87e-02	-4.15e-01	9.34e-03	3.86e-01	2.91e-02
**N-MeFOSAA**	3.36e-03	5.57e-01	2.60e-01	9.99e-02	-1.63e-02	3.14e-01	4.56e-02	2.48e-02	7.75e-01	6.79e-05	2.66e-01	5.66e-01	-9.61e-02	8.16e-02	3.17e-01	2.06e-02	-3.57e-02	-4.00e-01	1.12e-02	3.83e-01	2.99e-02
**N-EtFOSAA**	3.76e-03	5.09e-01	2.60e-01	1.01e-01	-1.73e-02	2.85e-01	4.66e-02	2.19e-02	7.69e-01	7.61e-05	2.69e-01	5.62e-01	-9.58e-02	8.42e-02	3.18e-01	2.04e-02	-3.29e-02	-3.98e-01	1.18e-02	3.80e-01	3.13e-02
**PFNA**	3.68e-03	5.22e-01	2.58e-01	1.03e-01	-1.68e-02	3.00e-01	4.61e-02	2.33e-02	7.73e-01	6.99e-05	2.48e-01	5.92e-01	-9.41e-02	9.06e-02	3.17e-01	2.06e-02	-3.62e-02	-3.89e-01	1.39e-02	3.86e-01	2.90e-02
**PFDA**	4.46e-03	4.39e-01	2.60e-01	1.01e-01	-1.79e-02	2.73e-01	4.60e-02	2.38e-02	7.69e-01	7.70e-05	2.68e-01	5.64e-01	-9.86e-02	7.70e-02	3.16e-01	2.11e-02	-2.38e-02	-3.75e-01	1.67e-02	3.85e-01	2.94e-02
**PFDS**	4.07e-03	4.75e-01	2.59e-01	1.02e-01	-1.71e-02	2.93e-01	4.59e-02	2.38e-02	7.72e-01	7.14e-05	2.59e-01	5.75e-01	-9.73e-02	7.93e-02	3.15e-01	2.15e-02	-3.10e-02	-3.83e-01	1.49e-02	3.83e-01	2.99e-02
**PFUnA**	3.72e-03	5.21e-01	2.53e-01	1.11e-01	-1.65e-02	3.14e-01	4.58e-02	2.43e-02	7.74e-01	6.85e-05	2.50e-01	5.90e-01	-9.58e-02	8.44e-02	3.14e-01	2.17e-02	-3.39e-02	-3.85e-01	1.47e-02	3.83e-01	3.03e-02
**PFDoA**	3.69e-03	5.27e-01	2.54e-01	1.10e-01	-1.63e-02	3.24e-01	4.57e-02	2.43e-02	7.76e-01	6.81e-05	2.51e-01	5.89e-01	-9.61e-02	8.36e-02	3.14e-01	2.19e-02	-3.63e-02	-3.89e-01	1.44e-02	3.83e-01	3.02e-02
**PFTrDA**	4.51e-03	4.41e-01	2.62e-01	9.84e-02	-1.80e-02	2.74e-01	4.61e-02	2.34e-02	7.68e-01	7.95e-05	2.64e-01	5.69e-01	-9.75e-02	7.90e-02	3.15e-01	2.15e-02	-2.30e-02	-3.74e-01	1.71e-02	3.86e-01	2.90e-02
**8:2 FTS**	4.29e-03	4.50e-01	2.50e-01	1.16e-01	-1.61e-02	3.20e-01	4.50e-02	2.71e-02	7.70e-01	7.37e-05	2.46e-01	5.95e-01	-9.55e-02	8.61e-02	3.21e-01	1.93e-02	-4.24e-02	-4.09e-01	1.03e-02	3.75e-01	3.40e-02
**4-OH-CB187**	6.21e-04	9.15e-01	2.87e-01	7.05e-02	-1.34e-02	4.11e-01	4.65e-02	2.22e-02	7.81e-01	6.10e-05	1.97e-01	6.72e-01	-9.67e-02	8.12e-02	3.28e-01	1.71e-02	-5.90e-02	-4.19e-01	8.90e-03	3.89e-01	2.79e-02
**3-OH-CB153**	-2.91e-03	6.48e-01	2.54e-01	1.09e-01	-1.30e-02	4.23e-01	4.23e-02	3.79e-02	7.89e-01	5.16e-05	2.41e-01	6.03e-01	-8.94e-02	1.08e-01	3.33e-01	1.55e-02	-5.18e-02	-4.19e-01	8.32e-03	3.82e-01	3.07e-02
**4-OH-CB107**	5.13e-03	4.07e-01	2.61e-01	9.95e-02	-1.81e-02	2.68e-01	4.68e-02	2.21e-02	7.75e-01	6.81e-05	2.57e-01	5.78e-01	-9.86e-02	7.68e-02	3.12e-01	2.31e-02	-2.44e-02	-3.75e-01	1.68e-02	3.82e-01	3.06e-02

β beta (regression coefficient)

Supplementary Table S27. Coefficients for the logistic regression for the fully adjusted model including Nordic born status used for evaluating per- and polyfluorinated substances (PFAS) and hydroxyl polychlorinated biphenyls (OH-PCBs) association with odds of multiple sclerosis (MS), in males (n = 458). The coefficients and the two-way ANOVA *P* value for respective covariates are presented in the table below.

	Age		Current BMI		BMI at age 20		Regular smoking		Irregular smoking		History of infectious mononucleosis		Sun exposure			Nordic born (ref = 0)	
	**β**	***P***	**β**	***P***	**β**	***P***	**β**	***P***	**β**	***P***	**β**	***P***	**Median β**	**High β**	***P***	**β**	***P***
**PFHxS**	1.02e-04	9.87e-01	-8.19e-03	6.41e-01	4.28e-02	6.00e-02	6.75e-01	3.07e-03	1.22e-01	8.10e-01	4.93e-01	2.33e-03	-3.87e-02	-4.63e-01	7.31e-03	1.89e-01	2.97e-01
**PFHpS**	-2.34e-03	7.18e-01	-7.48e-03	6.70e-01	4.09e-02	7.24e-02	6.71e-01	3.29e-03	1.29e-01	7.98e-01	4.89e-01	2.59e-03	-6.82e-02	-4.95e-01	4.98e-03	1.86e-01	3.06e-01
**PFOA**	-1.82e-03	7.78e-01	-7.20e-03	6.81e-01	4.25e-02	6.17e-02	6.63e-01	3.65e-03	1.05e-01	8.35e-01	4.92e-01	2.43e-03	-6.73e-02	-5.10e-01	3.79e-03	1.98e-01	2.74e-01
**PFOS**	-1.89e-03	7.71e-01	-5.53e-03	7.53e-01	4.17e-02	6.66e-02	6.75e-01	3.05e-03	1.18e-01	8.16e-01	4.89e-01	2.59e-03	-7.59e-02	-5.01e-01	4.78e-03	1.89e-01	2.97e-01
**FOSA**	-2.57e-03	6.95e-01	-4.97e-03	7.78e-01	4.33e-02	5.75e-02	6.95e-01	2.35e-03	9.33e-02	8.54e-01	5.04e-01	1.95e-03	-6.72e-02	-5.09e-01	3.80e-03	1.80e-01	3.22e-01
**N-MeFOSAA**	-6.40e-04	9.20e-01	-7.18e-03	6.82e-01	4.27e-02	6.08e-02	6.80e-01	2.84e-03	1.42e-01	7.79e-01	4.98e-01	2.16e-03	-4.66e-02	-4.82e-01	5.52e-03	1.87e-01	3.01e-01
**N-EtFOSAA**	-6.05e-06	9.99e-01	-8.66e-03	6.21e-01	4.40e-02	5.37e-02	6.72e-01	3.19e-03	1.39e-01	7.84e-01	4.97e-01	2.17e-03	-4.48e-02	-4.89e-01	5.02e-03	1.87e-01	3.03e-01
**PFNA**	-9.24e-04	8.85e-01	-6.79e-03	6.99e-01	4.29e-02	5.93e-02	6.76e-01	3.01e-03	1.02e-01	8.41e-01	5.02e-01	1.99e-03	-6.20e-02	-4.87e-01	5.55e-03	1.95e-01	2.82e-01
**PFDA**	-4.23e-05	9.95e-01	-7.79e-03	6.58e-01	4.28e-02	6.01e-02	6.78e-01	2.92e-03	1.16e-01	8.19e-01	4.91e-01	2.46e-03	-4.20e-02	-4.66e-01	7.06e-03	1.87e-01	3.02e-01
**PFDS**	-4.48e-04	9.44e-01	-5.97e-03	7.34e-01	4.29e-02	5.96e-02	6.84e-01	2.68e-03	1.46e-01	7.73e-01	4.98e-01	2.15e-03	-6.05e-02	-4.88e-01	5.36e-03	1.78e-01	3.25e-01
**PFUnA**	-1.14e-03	8.60e-01	-5.32e-03	7.64e-01	4.17e-02	6.68e-02	6.85e-01	2.64e-03	9.94e-02	8.45e-01	4.90e-01	2.52e-03	-5.70e-02	-4.74e-01	6.79e-03	1.81e-01	3.18e-01
**PFDoA**	-7.04e-04	9.14e-01	-5.95e-03	7.40e-01	4.22e-02	6.39e-02	6.84e-01	2.72e-03	1.11e-01	8.27e-01	4.92e-01	2.40e-03	-5.44e-02	-4.80e-01	6.25e-03	1.86e-01	3.06e-01
**PFTrDA**	-2.66e-04	9.68e-01	-7.41e-03	6.77e-01	4.27e-02	6.07e-02	6.78e-01	2.93e-03	1.17e-01	8.17e-01	4.91e-01	2.45e-03	-4.51e-02	-4.69e-01	7.06e-03	1.85e-01	3.09e-01
**8:2 FTS**	4.47e-04	9.44e-01	-6.42e-03	7.15e-01	4.09e-02	7.33e-02	6.77e-01	2.95e-03	1.19e-01	8.15e-01	5.09e-01	1.74e-03	-5.87e-02	-5.08e-01	3.85e-03	1.73e-01	3.40e-01
**4-OH-CB187**	-4.13e-03	5.31e-01	-3.75e-03	8.31e-01	4.37e-02	5.54e-02	6.68e-01	3.47e-03	5.62e-02	9.12e-01	5.07e-01	1.82e-03	-7.60e-02	-5.00e-01	4.76e-03	1.78e-01	3.27e-01
**3-OH-CB153**	-6.95e-03	3.31e-01	-3.06e-03	8.63e-01	3.84e-02	9.24e-02	6.87e-01	2.60e-03	1.07e-01	8.32e-01	5.15e-01	1.56e-03	-6.58e-02	-5.00e-01	4.39e-03	1.93e-01	2.87e-01
**4-OH-CB107**	2.00e-03	7.72e-01	-9.89e-03	5.77e-01	4.45e-02	5.21e-02	6.85e-01	2.67e-03	1.25e-01	8.05e-01	4.88e-01	2.62e-03	-2.82e-02	-4.50e-01	8.47e-03	1.85e-01	3.07e-01

ß beta (regression coefficient)

Supplementary Table S28. Coefficients for the logistic regression for the fully adjusted model including Nordic born used for evaluating per- and polyfluorinated substances (PFAS) and hydroxyl polychlorinated biphenyls (OH-PCBs) association with odds of multiple sclerosis (MS), in females (n = 1,356). The coefficients and the two-way ANOVA *P* value for respective covariates are presented in the table below.

	**Age**		**Current BMI**		**BMI at age 20**		**Regular smoking**		**Irregular smoking**		**Nr. childbirths**		**History of infectious mononucleosis**		**Sun exposure**			**Born in a Nordic (ref = 0)**	
	**β**	***P***	**β**	***P***	**β**	***P***	**β**	***P***	**β**	***P***	**β**	***P***	**β**	***P***	**Median β**	**High β**	***P***	**β**	***P***
**PFHxS**	5.12e-03	4.66e-01	-7.49e-03	6.69e-01	4.17e-02	6.71e-02	6.71e-01	3.30e-03	1.52e-01	7.64e-01	-1.04e-01	7.59e-02	4.86e-01	2.75e-03	-3.61e-02	-4.55e-01	8.65e-03	2.10e-01	2.48e-01
**PFHpS**	1.77e-03	8.02e-01	-6.91e-03	6.93e-01	4.03e-02	7.72e-02	6.65e-01	3.59e-03	1.48e-01	7.69e-01	-8.28e-02	1.47e-01	4.84e-01	2.93e-03	-6.38e-02	-4.87e-01	5.75e-03	2.04e-01	2.62e-01
**PFOA**	2.29e-03	7.46e-01	-6.73e-03	7.01e-01	4.17e-02	6.73e-02	6.59e-01	3.89e-03	1.28e-01	7.99e-01	-8.26e-02	1.51e-01	4.86e-01	2.77e-03	-6.19e-02	-4.98e-01	4.78e-03	2.14e-01	2.38e-01
**PFOS**	2.49e-03	7.24e-01	-5.12e-03	7.71e-01	4.09e-02	7.31e-02	6.68e-01	3.41e-03	1.41e-01	7.80e-01	-9.13e-02	1.10e-01	4.83e-01	2.96e-03	-7.12e-02	-4.93e-01	5.56e-03	2.08e-01	2.52e-01
**FOSA**	1.97e-03	7.80e-01	-4.18e-03	8.13e-01	4.22e-02	6.46e-02	6.88e-01	2.67e-03	1.19e-01	8.14e-01	-9.98e-02	8.03e-02	4.97e-01	2.28e-03	-6.73e-02	-5.06e-01	4.21e-03	2.01e-01	2.68e-01
**N-MeFOSAA**	3.90e-03	5.73e-01	-6.39e-03	7.16e-01	4.15e-02	6.83e-02	6.73e-01	3.21e-03	1.66e-01	7.43e-01	-1.00e-01	8.02e-02	4.91e-01	2.54e-03	-4.67e-02	-4.78e-01	6.17e-03	2.09e-01	2.51e-01
**N-EtFOSAA**	4.53e-03	5.09e-01	-7.76e-03	6.58e-01	4.28e-02	6.11e-02	6.65e-01	3.60e-03	1.63e-01	7.47e-01	-1.01e-01	8.05e-02	4.90e-01	2.55e-03	-4.54e-02	-4.84e-01	5.66e-03	2.08e-01	2.52e-01
**PFNA**	3.50e-03	6.15e-01	-6.28e-03	7.20e-01	4.18e-02	6.64e-02	6.69e-01	3.38e-03	1.28e-01	8.00e-01	-9.34e-02	1.04e-01	4.94e-01	2.39e-03	-5.78e-02	-4.80e-01	6.43e-03	2.14e-01	2.39e-01
**PFDA**	4.64e-03	5.04e-01	-7.15e-03	6.85e-01	4.17e-02	6.76e-02	6.70e-01	3.35e-03	1.44e-01	7.75e-01	-1.01e-01	7.90e-02	4.86e-01	2.83e-03	-4.05e-02	-4.60e-01	7.99e-03	2.10e-01	2.49e-01
**PFDS**	4.09e-03	5.50e-01	-5.12e-03	7.71e-01	4.18e-02	6.71e-02	6.77e-01	3.04e-03	1.70e-01	7.36e-01	-1.01e-01	7.65e-02	4.91e-01	2.53e-03	-6.11e-02	-4.85e-01	5.87e-03	2.00e-01	2.71e-01
**PFUnA**	3.40e-03	6.27e-01	-4.92e-03	7.81e-01	4.08e-02	7.36e-02	6.77e-01	3.04e-03	1.27e-01	8.02e-01	-9.67e-02	9.15e-02	4.84e-01	2.91e-03	-5.46e-02	-4.69e-01	7.50e-03	2.03e-01	2.64e-01
**PFDoA**	3.98e-03	5.72e-01	-5.64e-03	7.53e-01	4.12e-02	7.08e-02	6.75e-01	3.16e-03	1.38e-01	7.84e-01	-9.94e-02	8.37e-02	4.86e-01	2.80e-03	-5.10e-02	-4.72e-01	7.24e-03	2.08e-01	2.53e-01
**PFTrDA**	4.35e-03	5.39e-01	-6.63e-03	7.10e-01	4.16e-02	6.84e-02	6.70e-01	3.32e-03	1.43e-01	7.77e-01	-1.01e-01	7.76e-02	4.85e-01	2.86e-03	-4.49e-02	-4.65e-01	7.85e-03	2.07e-01	2.56e-01
**8:2 FTS**	4.87e-03	4.77e-01	-5.65e-03	7.48e-01	3.99e-02	8.13e-02	6.70e-01	3.33e-03	1.44e-01	7.76e-01	-9.88e-02	8.71e-02	5.02e-01	2.08e-03	-5.85e-02	-5.03e-01	4.45e-03	1.95e-01	2.84e-01
**4-OH-CB187**	3.97e-04	9.55e-01	-2.97e-03	8.66e-01	4.26e-02	6.27e-02	6.60e-01	3.93e-03	8.12e-02	8.72e-01	-9.91e-02	8.39e-02	5.01e-01	2.13e-03	-7.65e-02	-4.97e-01	5.26e-03	1.99e-01	2.74e-01
**3-OH-CB153**	-2.33e-03	7.62e-01	-2.57e-03	8.85e-01	3.77e-02	9.99e-02	6.80e-01	2.94e-03	1.32e-01	7.94e-01	-9.34e-02	1.05e-01	5.08e-01	1.86e-03	-6.51e-02	-4.95e-01	4.94e-03	2.13e-01	2.41e-01
**4-OH-CB107**	7.00e-03	3.46e-01	-9.20e-03	6.04e-01	4.36e-02	5.76e-02	6.80e-01	2.96e-03	1.52e-01	7.63e-01	-1.05e-01	7.08e-02	4.80e-01	3.15e-03	-2.78e-02	-4.46e-01	9.50e-03	2.07e-01	2.54e-01

β beta (regression coefficient)

Supplementary Table S29. Associations between single serum per- and polyfluorinated substances (PFAS) and hydroxyl polychlorinated biphenyls (OH-PCBs), and the odds ratio of multiple sclerosis (MS) (n = 1,814). Logistic regression analysis was used to calculate the odds ratios per doubling of substance serum concentrations. Calculations based on the fully adjusted model including Nordic born status. No adjustments were made for multiple comparisons.

Substance (ng/ml)	Males and Females		Males		Females	
	OR (95% CI)	*P*	OR (95% CI)	*P*	OR (95% CI)	*P*
**PFHxS**	0.973 (0.903 - 1.049)	0.479	1.006 (0.919 - 1.101)	0.897	0.992 (0.905 - 1.087)	0.862
**PFHpS**	1.051 (0.976 - 1.133)	0.189	1.097 (0.998 - 1.206)	0.054	1.082 (0.983 - 1.191)	0.106
**PFOA**	1.052 (0.953 - 1.161)	0.314	1.112 (0.990 - 1.249)	0.075	1.090 (0.967 - 1.228)	0.157
**PFOS**	1.044 (0.968 - 1.127)	0.266	1.079 (0.982 - 1.186)	0.115	1.068 (0.971 - 1.175)	0.174
**FOSA**	1.068 (0.973 - 1.173)	0.166	1.101 (0.989 - 1.226)	0.078	1.100 (0.988 - 1.225)	0.082
**N-MeFOSAA**	1.038 (0.962 - 1.120)	0.341	1.041 (0.954 - 1.136)	0.371	1.039 (0.952 - 1.134)	0.396
**N-EtFOSAA**	1.030 (0.956 - 1.110)	0.438	1.043 (0.957 - 1.136)	0.341	1.041 (0.955 - 1.134)	0.366
**PFNA**	1.022 (0.937 - 1.113)	0.628	1.066 (0.962 - 1.182)	0.222	1.053 (0.949 - 1.169)	0.330
**PFDA**	0.986 (0.908 - 1.070)	0.732	1.010 (0.919 - 1.110)	0.830	1.004 (0.913 - 1.104)	0.935
**PFDS**	1.006 (0.940 - 1.075)	0.871	1.062 (0.985 - 1.146)	0.119	1.062 (0.985 - 1.146)	0.118
**PFUnA**	1.013 (0.942 - 1.090)	0.726	1.047 (0.959 - 1.144)	0.302	1.040 (0.952 - 1.136)	0.383
**PFDoA**	1.016 (0.922 - 1.119)	0.750	1.034 (0.924 - 1.157)	0.561	1.025 (0.916 - 1.148)	0.665
**PFTrDA**	0.993 (0.946 - 1.044)	0.796	1.006 (0.950 - 1.066)	0.831	1.005 (0.949 - 1.065)	0.859
**8:2 FTS**	1.027 (0.976 - 1.081)	0.307	1.041 (0.982 - 1.104)	0.175	1.039 (0.980 - 1.102)	0.204
**4-OH-CB187**	1.082 (1.019 - 1.148)	0.010	1.092 (1.019 - 1.171)	0.013	1.091 (1.018 - 1.17)	0.014
**3-OH-CB153**	1.055 (1.011 - 1.101)	0.015	1.057 (1.006 - 1.111)	0.030	1.053 (1.002 - 1.107)	0.041
**4-OH-CB107**	0.989 (0.937 - 1.043)	0.685	0.922 (0.922 - 1.042)	0.519	0.977 (0.918 - 1.039)	0.451

Model was adjusted for age, sex, BM at sample collection, BMI at the age of 20, number of childbirths, regular smoking, irregular smoking, history of infectious mononucleosis, sun exposure, born in a Nordic country, and residential area (as a random effect). Sex was excluded as a covariate from the sex-specific models.
OR, odds ratio; *CI*, confidence interval

Supplementary Table S30. Coefficients for the logistic regression for the fully adjusted model, residential area not taken into account, used for evaluating per- and polyfluorinated substances (PFAS) and hydroxyl polychlorinated biphenyls (OH-PCBs) association with odds of multiple sclerosis (MS), males and females (n = 1,814). The coefficients and the two-way ANOVA *P* value for respective covariates are presented in the table below.

	Age		Sex		Current BMI		BMI at age 20		Regular smoking		Irregular smoking		Nr. childbirths		History of infectious mononucleosis		Sun exposure			Swedish born (ref = 0)	
	β	*P*	β	*P*	β	*P*	β	*P*	β	*P*	β	*P*	β	*P*	β	*P*	Median β	High β	*P*	β	*P*
**PFHxS**	5.46e-03	3.34e-01	2.45e-01	1.12e-01	-2.17e-02	1.69e-01	4.81e-02	1.31e-02	7.87e-01	2.27e-05	2.55e-01	5.81e-01	-1.11e-01	3.83e-02	3.31e-01	1.19e-02	-2.49e-02	-4.08e-01	5.31e-03	2.52e-01	9.86e-02
**PFHpS**	2.70e-03	6.34e-01	2.93e-01	5.69e-02	-2.08e-02	1.87e-01	4.77e-02	1.36e-02	7.77e-01	2.88e-05	2.35e-01	6.11e-01	-9.29e-02	7.72e-02	3.31e-01	1.21e-02	-4.59e-02	-4.37e-01	3.16e-03	2.51e-01	1.01e-01
**PFOA**	3.15e-03	5.76e-01	2.57e-01	9.13e-02	-2.07e-02	1.88e-01	4.83e-02	1.25e-02	7.74e-01	3.10e-05	2.27e-01	6.23e-01	-9.27e-02	7.96e-02	3.31e-01	1.21e-02	-4.60e-02	-4.45e-01	2.76e-03	2.58e-01	9.10e-02
**PFOS**	3.08e-03	5.86e-01	2.73e-01	7.38e-02	-1.93e-02	2.23e-01	4.78e-02	1.34e-02	7.87e-01	2.24e-05	2.29e-01	6.19e-01	-9.87e-02	6.04e-02	3.32e-01	1.18e-02	-5.08e-02	-4.41e-01	3.05e-03	2.53e-01	9.74e-02
**FOSA**	2.67e-03	6.40e-01	2.58e-01	9.01e-02	-1.90e-02	2.29e-01	4.84e-02	1.25e-02	7.99e-01	1.76e-05	2.18e-01	6.37e-01	-1.05e-01	4.61e-02	3.35e-01	1.12e-02	-4.94e-02	-4.52e-01	2.44e-03	2.46e-01	1.08e-01
**N-MeFOSAA**	3.77e-03	4.99e-01	2.59e-01	8.92e-02	-2.01e-02	2.03e-01	4.75e-02	1.42e-02	7.87e-01	2.27e-05	2.57e-01	5.77e-01	-1.04e-01	4.63e-02	3.34e-01	1.13e-02	-3.79e-02	-4.40e-01	2.80e-03	2.51e-01	1.01e-01
**N-EtFOSAA**	4.27e-03	4.41e-01	2.56e-01	9.28e-02	-2.14e-02	1.75e-01	4.89e-02	1.16e-02	7.79e-01	2.71e-05	2.61e-01	5.73e-01	-1.04e-01	4.86e-02	3.34e-01	1.13e-02	-3.36e-02	-4.38e-01	2.96e-03	2.48e-01	1.04e-01
**PFNA**	4.11e-03	4.62e-01	2.55e-01	9.38e-02	-2.07e-02	1.91e-01	4.82e-02	1.27e-02	7.84e-01	2.40e-05	2.33e-01	6.14e-01	-1.01e-01	5.46e-02	3.34e-01	1.13e-02	-3.93e-02	-4.27e-01	3.84e-03	2.56e-01	9.33e-02
**PFDA**	5.01e-03	3.71e-01	2.57e-01	9.24e-02	-2.17e-02	1.71e-01	4.80e-02	1.31e-02	7.80e-01	2.63e-05	2.53e-01	5.85e-01	-1.07e-01	4.29e-02	3.32e-01	1.19e-02	-2.60e-02	-4.11e-01	4.80e-03	2.54e-01	9.62e-02
**PFDS**	4.70e-03	3.95e-01	2.56e-01	9.28e-02	-2.11e-02	1.82e-01	4.81e-02	1.30e-02	7.82e-01	2.53e-05	2.48e-01	5.91e-01	-1.06e-01	4.38e-02	3.31e-01	1.20e-02	-3.15e-02	-4.18e-01	4.36e-03	2.52e-01	9.84e-02
**PFUnA**	4.28e-03	4.49e-01	2.51e-01	1.01e-01	-2.04e-02	1.98e-01	4.79e-02	1.32e-02	7.84e-01	2.40e-05	2.37e-01	6.08e-01	-1.05e-01	4.72e-02	3.30e-01	1.22e-02	-3.42e-02	-4.19e-01	4.34e-03	2.52e-01	9.86e-02
**PFDoA**	4.11e-03	4.69e-01	2.49e-01	1.03e-01	-1.98e-02	2.18e-01	4.78e-02	1.36e-02	7.88e-01	2.27e-05	2.35e-01	6.10e-01	-1.04e-01	4.79e-02	3.30e-01	1.23e-02	-3.94e-02	-4.27e-01	3.96e-03	2.52e-01	9.86e-02
**PFTrDA**	4.33e-03	4.47e-01	2.53e-01	9.77e-02	-2.05e-02	1.99e-01	4.79e-02	1.34e-02	7.86e-01	2.40e-05	2.39e-01	6.04e-01	-1.06e-01	4.44e-02	3.30e-01	1.22e-02	-3.53e-02	-4.22e-01	4.24e-03	2.51e-01	1.01e-01
**8:2 FTS**	4.86e-03	3.78e-01	2.47e-01	1.07e-01	-2.01e-02	2.04e-01	4.69e-02	1.56e-02	7.79e-01	2.71e-05	2.36e-01	6.09e-01	-1.05e-01	4.81e-02	3.37e-01	1.07e-02	-4.21e-02	-4.42e-01	2.96e-03	2.45e-01	1.10e-01
**4-OH-CB187**	1.71e-03	7.64e-01	2.83e-01	6.43e-02	-1.85e-02	2.41e-01	4.89e-02	1.15e-02	7.84e-01	2.43e-05	1.90e-01	6.80e-01	-1.06e-01	4.37e-02	3.43e-01	9.49e-03	-5.77e-02	-4.49e-01	2.66e-03	2.60e-01	8.86e-02
**3-OH-CB153**	-2.98e-03	6.30e-01	2.49e-01	1.03e-01	-1.71e-02	2.82e-01	4.40e-02	2.36e-02	8.01e-01	1.66e-05	2.26e-01	6.24e-01	-9.60e-02	7.00e-02	3.52e-01	7.94e-03	-5.62e-02	-4.62e-01	1.87e-03	2.47e-01	1.06e-01
**4-OH-CB107**	5.68e-03	3.42e-01	2.59e-01	8.98e-02	-2.21e-02	1.64e-01	4.89e-02	1.21e-02	7.85e-01	2.39e-05	2.46e-01	5.94e-01	-1.08e-01	4.21e-02	3.28e-01	1.29e-02	-2.55e-02	-4.09e-01	5.09e-03	2.52e-01	9.84e-02

ß, beta (regression coefficient)

Supplementary Table S31. Coefficients for the logistic regression for the fully adjusted model, residential area not taken into account, used for evaluating per- and polyfluorinated substances (PFAS) and hydroxyl polychlorinated biphenyls (OH-PCBs) association with odds of multiple sclerosis (MS), in males (n = 458). The coefficients and the two-way ANOVA *P* value for respective covariates are presented in the table below.

	**Age**		**Current BMI**		**BMI at age 20**		**Regular smoking**		**Irregular smoking**		**History of infectious mononucleosis**		**Sun exposure**			**Swedish born (ref = 0)**	
	**β**	***P***	**β**	***P***	**β**	***P***	**β**	***P***	**β**	***P***	**β**	***P***	**Median β**	**High β**	***P***	**β**	***P***
**PFHxS**	-5.83e-04	9.26e-01	-1.10e-02	5.17e-01	4.38e-02	4.20e-02	6.82e-01	1.76e-03	1.22e-01	8.10e-01	4.81e-01	1.98e-03	-2.63e-02	-4.95e-01	1.95e-03	2.10e-01	2.31e-01
**PFHpS**	-3.30e-03	6.00e-01	-1.06e-02	5.33e-01	4.17e-02	5.31e-02	6.81e-01	1.82e-03	1.32e-01	7.93e-01	4.77e-01	2.23e-03	-5.28e-02	-5.26e-01	1.25e-03	2.04e-01	2.46e-01
**PFOA**	-2.74e-03	6.61e-01	-9.99e-03	5.57e-01	4.33e-02	4.41e-02	6.68e-01	2.21e-03	1.06e-01	8.33e-01	4.79e-01	2.12e-03	-5.74e-02	-5.50e-01	7.76e-04	2.17e-01	2.17e-01
**PFOS**	-2.71e-03	6.67e-01	-8.30e-03	6.27e-01	4.24e-02	4.92e-02	6.86e-01	1.66e-03	1.21e-01	8.11e-01	4.77e-01	2.18e-03	-6.06e-02	-5.32e-01	1.22e-03	2.08e-01	2.36e-01
**FOSA**	-2.78e-03	6.61e-01	-8.36e-03	6.25e-01	4.45e-02	3.90e-02	7.02e-01	1.30e-03	9.87e-02	8.46e-01	4.87e-01	1.78e-03	-4.73e-02	-5.34e-01	1.02e-03	1.98e-01	2.60e-01
**N-MeFOSAA**	-1.23e-03	8.42e-01	-9.93e-03	5.60e-01	4.36e-02	4.30e-02	6.93e-01	1.49e-03	1.52e-01	7.65e-01	4.87e-01	1.77e-03	-3.34e-02	-5.17e-01	1.32e-03	2.05e-01	2.42e-01
**N-EtFOSAA**	-4.75e-04	9.38e-01	-1.16e-02	4.94e-01	4.51e-02	3.67e-02	6.83e-01	1.74e-03	1.46e-01	7.73e-01	4.85e-01	1.86e-03	-3.03e-02	-5.23e-01	1.19e-03	2.04e-01	2.45e-01
**PFNA**	-1.50e-03	8.08e-01	-9.69e-03	5.70e-01	4.39e-02	4.12e-02	6.89e-01	1.58e-03	1.03e-01	8.39e-01	4.89e-01	1.68e-03	-5.12e-02	-5.20e-01	1.43e-03	2.15e-01	2.20e-01
**PFDA**	-4.46e-04	9.43e-01	-1.08e-02	5.29e-01	4.39e-02	4.13e-02	6.88e-01	1.60e-03	1.19e-01	8.15e-01	4.79e-01	2.09e-03	-2.71e-02	-4.94e-01	1.99e-03	2.07e-01	2.38e-01
**PFDS**	-8.26e-04	8.93e-01	-9.31e-03	5.85e-01	4.40e-02	4.12e-02	6.92e-01	1.49e-03	1.47e-01	7.72e-01	4.87e-01	1.77e-03	-4.21e-02	-5.14e-01	1.49e-03	1.99e-01	2.57e-01
**PFUnA**	-1.45e-03	8.17e-01	-8.90e-03	6.04e-01	4.33e-02	4.40e-02	6.93e-01	1.47e-03	1.05e-01	8.37e-01	4.79e-01	2.09e-03	-3.75e-02	-4.98e-01	2.01e-03	2.02e-01	2.49e-01
**PFDoA**	-1.07e-03	8.65e-01	-8.99e-03	6.05e-01	4.33e-02	4.46e-02	6.93e-01	1.48e-03	1.15e-01	8.21e-01	4.80e-01	2.02e-03	-3.82e-02	-5.07e-01	1.74e-03	2.05e-01	2.42e-01
**PFTrDA**	-1.14e-03	8.57e-01	-9.53e-03	5.81e-01	4.36e-02	4.28e-02	6.91e-01	1.53e-03	1.16e-01	8.19e-01	4.78e-01	2.12e-03	-3.74e-02	-5.05e-01	1.79e-03	2.01e-01	2.53e-01
**8:2 FTS**	-6.26e-05	9.92e-01	-9.30e-03	5.86e-01	4.19e-02	5.27e-02	6.85e-01	1.65e-03	1.25e-01	8.05e-01	4.95e-01	1.52e-03	-4.09e-02	-5.30e-01	1.11e-03	1.93e-01	2.72e-01
**4-OH-CB187**	-4.01e-03	5.28e-01	-8.03e-03	6.38e-01	4.56e-02	3.46e-02	6.74e-01	2.03e-03	6.29e-02	9.01e-01	4.95e-01	1.54e-03	-6.12e-02	-5.25e-01	1.41e-03	2.04e-01	2.47e-01
**3-OH-CB153**	-8.01e-03	2.45e-01	-5.90e-03	7.31e-01	3.93e-02	6.98e-02	6.99e-01	1.37e-03	1.07e-01	8.32e-01	5.05e-01	1.24e-03	-5.65e-02	-5.36e-01	1.01e-03	2.08e-01	2.36e-01
**4-OH-CB107**	1.63e-03	8.06e-01	-1.29e-02	4.53e-01	4.57e-02	3.51e-02	6.95e-01	1.45e-03	1.29e-01	7.99e-01	4.77e-01	2.18e-03	-1.44e-02	-4.78e-01	2.52e-03	2.06e-01	2.40e-01

ß, beta (regression coefficient)

Supplementary Table S32. Coefficients for the logistic regression for the fully adjusted model, residential area not taken into account, used for evaluating per- and polyfluorinated substances (PFAS) and hydroxyl polychlorinated biphenyls (OH-PCBs) association with odds of multiple sclerosis (MS), in females (n = 1,356). The coefficients and the two-way ANOVA *P* value for respective covariates are presented in the table below.

	Age		Current BMI		BMI at age 20		Regular smoking		Irregular smoking		Nr. childbirths		History of infectious mononucleosis		Sun exposure			Swedish born (ref = 0)	
	**β**	***P***	**β**	***P***	**β**	***P***	**β**	***P***	**β**	***P***	**β**	***P***	**β**	***P***	**Median β**	**High β**	***P***	**β**	***P***
**PFHxS**	4.92e-03	4.73e-01	-1.02e-02	5.50e-01	4.24e-02	4.94e-02	6.81e-01	1.85e-03	1.55e-01	7.59e-01	-1.12e-01	4.32e-02	4.74e-01	2.40e-03	-2.37e-02	-4.88e-01	2.28e-03	2.30e-01	1.90e-01
**PFHpS**	1.24e-03	8.57e-01	-9.92e-03	5.60e-01	4.09e-02	5.85e-02	6.77e-01	1.96e-03	1.54e-01	7.60e-01	-8.99e-02	9.81e-02	4.71e-01	2.59e-03	-4.85e-02	-5.20e-01	1.43e-03	2.22e-01	2.07e-01
**PFOA**	1.68e-03	8.06e-01	-9.44e-03	5.80e-01	4.24e-02	4.97e-02	6.67e-01	2.30e-03	1.31e-01	7.95e-01	-8.77e-02	1.08e-01	4.73e-01	2.48e-03	-5.17e-02	-5.39e-01	1.00e-03	2.33e-01	1.86e-01
**PFOS**	2.15e-03	7.54e-01	-7.77e-03	6.49e-01	4.13e-02	5.61e-02	6.81e-01	1.83e-03	1.47e-01	7.71e-01	-9.98e-02	6.58e-02	4.71e-01	2.58e-03	-5.60e-02	-5.25e-01	1.40e-03	2.27e-01	1.97e-01
**FOSA**	2.27e-03	7.40e-01	-7.42e-03	6.65e-01	4.31e-02	4.63e-02	6.97e-01	1.46e-03	1.28e-01	8.00e-01	-1.10e-01	4.29e-02	4.79e-01	2.17e-03	-4.72e-02	-5.32e-01	1.11e-03	2.19e-01	2.13e-01
**N-MeFOSAA**	3.80e-03	5.71e-01	-9.00e-03	5.98e-01	4.22e-02	5.09e-02	6.88e-01	1.66e-03	1.78e-01	7.24e-01	-1.09e-01	4.33e-02	4.79e-01	2.17e-03	-3.33e-02	-5.14e-01	1.45e-03	2.26e-01	1.98e-01
**N-EtFOSAA**	4.56e-03	4.94e-01	-1.06e-02	5.34e-01	4.36e-02	4.41e-02	6.77e-01	1.95e-03	1.73e-01	7.31e-01	-1.10e-01	4.37e-02	4.77e-01	2.27e-03	-3.07e-02	-5.19e-01	1.33e-03	2.26e-01	2.00e-01
**PFNA**	3.38e-03	6.18e-01	-9.06e-03	5.95e-01	4.26e-02	4.83e-02	6.83e-01	1.77e-03	1.32e-01	7.93e-01	-1.02e-01	6.23e-02	4.81e-01	2.09e-03	-4.63e-02	-5.13e-01	1.65e-03	2.34e-01	1.84e-01
**PFDA**	4.77e-03	4.80e-01	-9.98e-03	5.60e-01	4.25e-02	4.93e-02	6.81e-01	1.81e-03	1.51e-01	7.65e-01	-1.11e-01	4.16e-02	4.73e-01	2.48e-03	-2.55e-02	-4.90e-01	2.20e-03	2.30e-01	1.92e-01
**PFDS**	4.23e-03	5.25e-01	-8.30e-03	6.27e-01	4.26e-02	4.89e-02	6.87e-01	1.67e-03	1.74e-01	7.30e-01	-1.11e-01	4.02e-02	4.79e-01	2.16e-03	-4.25e-02	-5.12e-01	1.59e-03	2.21e-01	2.10e-01
**PFUnA**	3.66e-03	5.91e-01	-8.30e-03	6.29e-01	4.20e-02	5.15e-02	6.87e-01	1.67e-03	1.35e-01	7.89e-01	-1.08e-01	4.79e-02	4.72e-01	2.51e-03	-3.55e-02	-4.96e-01	2.16e-03	2.24e-01	2.03e-01
**PFDoA**	4.15e-03	5.44e-01	-8.56e-03	6.23e-01	4.20e-02	5.19e-02	6.86e-01	1.70e-03	1.46e-01	7.73e-01	-1.09e-01	4.47e-02	4.73e-01	2.44e-03	-3.47e-02	-5.01e-01	1.99e-03	2.27e-01	1.96e-01
**PFTrDA**	4.00e-03	5.61e-01	-8.65e-03	6.17e-01	4.22e-02	5.08e-02	6.85e-01	1.72e-03	1.45e-01	7.74e-01	-1.11e-01	4.16e-02	4.71e-01	2.56e-03	-3.67e-02	-5.02e-01	1.96e-03	2.23e-01	2.06e-01
**8:2 FTS**	4.90e-03	4.61e-01	-8.40e-03	6.23e-01	4.06e-02	6.12e-02	6.80e-01	1.84e-03	1.53e-01	7.62e-01	-1.09e-01	4.62e-02	4.86e-01	1.90e-03	-4.05e-02	-5.26e-01	1.27e-03	2.15e-01	2.23e-01
**4-OH-CB187**	1.05e-03	8.79e-01	-7.05e-03	6.80e-01	4.41e-02	4.15e-02	6.68e-01	2.27e-03	9.10e-02	8.57e-01	-1.10e-01	4.30e-02	4.87e-01	1.88e-03	-6.16e-02	-5.23e-01	1.52e-03	2.25e-01	2.02e-01
**3-OH-CB153**	-2.90e-03	6.97e-01	-5.32e-03	7.57e-01	3.83e-02	7.81e-02	6.93e-01	1.54e-03	1.35e-01	7.89e-01	-1.01e-01	6.51e-02	4.97e-01	1.53e-03	-5.52e-02	-5.33e-01	1.13e-03	2.28e-01	1.96e-01
**4-OH-CB107**	7.22e-03	3.16e-01	-1.21e-02	4.82e-01	4.45e-02	4.07e-02	6.92e-01	1.57e-03	1.59e-01	7.52e-01	-1.15e-01	3.58e-02	4.68e-01	2.74e-03	-1.38e-02	-4.75e-01	2.79e-03	2.28e-01	1.95e-01

ß. beta (regression coefficient)

Supplementary Table S33. Associations between single serum per- and polyfluorinated substances (PFAS) and hydroxyl polychlorinated biphenyls (OH-PCBs), and the odds ratio of multiple sclerosis (MS) (n = 1,814). Odds ratios are calculated per doubling of substance serum concentrations, using logistic regression. Calculations based on the fully adjusted model where residential area is not taken into account. No adjustments were made for multiple comparisons.

Substance (ng/ml)	Males and Females		Males		Females	
	OR (95% CI)	*P*	OR (95% CI)	*P*	OR (95% CI)	*P*
**PFHxS**	0.979 (0.910 - 1.053)	0.573	1.016 (0.930 - 1.109)	0.730	1.000 (0.915 - 1.094)	0.998
**PFHpS**	1.062 (0.988 - 1.143)	0.106	1.110 (1.014 - 1.219)	0.025	1.094 (0.998 - 1.203)	0.057
**PFOA**	1.067 (0.970 - 1.174)	0.183	1.133 (1.014 - 1.269)	0.029	1.110 (0.991 - 1.247)	0.074
**PFOS**	1.054 (0.979 - 1.136)	0.164	1.088 (0.994 - 1.195)	0.070	1.077 (0.983 - 1.183)	0.115
**FOSA**	1.070 (0.978 - 1.171)	0.141	1.090 (0.983 - 1.210)	0.104	1.088 (0.981 - 1.208)	0.112
**N-MeFOSAA**	1.048 (0.973 - 1.129)	0.220	1.052 (0.966 - 1.147)	0.244	1.050 (0.964 - 1.144)	0.266
**N-EtFOSAA**	1.039 (0.966 - 1.118)	0.306	1.051 (0.967 - 1.143)	0.241	1.049 (0.964 - 1.141)	0.267
**PFNA**	1.031 (0.948 - 1.121)	0.476	1.076 (0.975 - 1.190)	0.150	1.061 (0.961 - 1.175)	0.245
**PFDA**	0.991 (0.915 - 1.073)	0.820	1.013 (0.925 - 1.109)	0.786	1.006 (0.918 - 1.102)	0.900
**PFDS**	1.007 (0.944 - 1.075)	0.825	1.058 (0.983 - 1.139)	0.135	1.058 (0.983 - 1.140)	0.135
**PFUnA**	1.015 (0.946 - 1.090)	0.674	1.043 (0.959 - 1.135)	0.332	1.035 (0.952 - 1.128)	0.420
**PFDoA**	1.024 (0.933 - 1.125)	0.614	1.035 (0.929 - 1.153)	0.537	1.025 (0.920 - 1.143)	0.651
**PFTrDA**	1.008 (0.961 - 1.057)	0.746	1.016 (0.961 - 1.075)	0.570	1.015 (0.960 - 1.073)	0.606
**8:2 FTS**	1.026 (0.976 - 1.079)	0.310	1.038 (0.981 - 1.099)	0.198	1.035 (0.978 - 1.097)	0.233
**4-OH-CB187**	1.069 (1.010 - 1.132)	0.021	1.081 (1.012 - 1.155)	0.022	1.080 (1.011 - 1.155)	0.023
**3-OH-CB153**	1.061 (1.018 - 1.107)	0.005	1.064 (1.014 - 1.117)	0.012	1.060 (1.009 - 1.113)	0.020
**4-OH-CB107**	0.990 (0.940 - 1.042)	0.698	0.979 (0.922 - 1.039)	0.487	0.975 (0.918 - 1.035)	0.409

Model was adjusted for age, sex, BM at sample collection, BMI at the age of 20, number of childbirths, regular smoking, irregular smoking, history of infectious mononucleosis, sun exposure, and Swedish born. Sex was excluded as a covariate in the sex-specific models.
OR, odds ratio; *CI*, confidence interval

Supplementary Table S34. General and clinical characteristics for the 551 individuals included in the Cox proportional hazard analysis regards confirmed disability worsening (CDW).

Characteristic	RRMS	PMS
N (F/M)	375 (75.9%) / 119 (24.1%)	39 (68.4%) / 18 (31.6%)
Age: Mean ±SD, F / M	38.9 ± 10.3 / 37.3 ± 10.1	51.9 ± 8.7 / 48.9 ± 8.2
BMI at sample collection: Mean ±SD, F / M	25.1 ± 5.2 / 25.2 ± 4.3	25.4 ± 4.7 / 25.1 ± 2.8
BMI at age of 20: Mean ±SD, F / M	22.5 ± 4.1 / 23.4 ± 3.5	21.2 ± 4.1 / 23.0 ± 4.5
Disease duration		
Months: Mean ±SD, F / M	9.5 ± 20.5 / 9.3 ± 15.1	9.6 ± 11.5 / 10.0 ± 10.3
Number of hospital visits		
Mean ±SD, F / M	11.5 ± 5.6 / 12.3 ± 6.7	8.9 ± 4.7 / 8.9 ± 4.6
Median ±SD, F / M	11.0 ± 5.6 / 11.5 ± 6.7	8.0 ± 4.7 / 8.0 ± 4.6
Follow-up time (years)		
Mean ±SD, F / M	10.7 ± 3.5 / 10.2 ± 3.7	9.0 ± 4.1 / 9.0 ± 4.1
Median ±SD, F / M	10.9 ± 3.5 / 10.5 ± 3.7	9.7 ± 4.1 / 8.7 ± 4.1
EDSS		
Mean ±SD, F / M	1.5 ± 1.2 / 2.1 ± 1.6	3.8 ± 1.5 / 3.7 ± 1.6
Median ±SD, F / M	1.5 ±1.2 / 2.0 ±1.6	3.5 ±1.5 / 3.5 ±1.6
Treatment		
% undergoing treatment	8.3 %	15.8 %
Regular smoking		
% Yes / No / NA	23.8% / 68.5% / 7.7%	16.1% / 76.8% / 7.1%
Years: Mean ±SD, F / M	20.7 ± 9.4 / 20.3 ± 13.4	30 ± 7.8 / 32.5 ± 0.7
Irregular smoking		
% Yes / No / NA	4.1% / 95.9% / 0.0%	3.6% / 96.4% / 0.0%
Years: Mean ±SD, F / M	12.5 ± 11.6 / 9.7 ± 6.4	NA* / NA*
Passive smoking (indoors)		
% Yes / No / NA	3.1% / 89.0% / 7.9%	5.4% / 87.5% / 7.1%
Years: Mean ±SD, F / M	30.3 ± 12.8 / 26.3 ± 16.5	NA** / 24.3 ± 18.7
Swedish snuff		
% Yes / No / NA	8.1% / 78.5% / 13.4%	8.9% / 82.2% / 8.9%
Years: Mean ±SD, F / M	8.4 ± 6.9 / 11.7 ± 8.9	NA / 19.5 ± 19.8
Alcohol consumption***		
% Yes / No / NA	61.3% / 31.1% / 7.6%	64.3% / 28.6% / 7.1%
Volume (cl): Mean ±SD, F / M	0.9 ± 2.9 / 4.2 ± 9.6	0.6 ± 1.6 / 3.6 ± 5.7
Infectious mononucleosis		
% Yes / No / NA	22.2% / 69.6% / 8.3%	19.6% / 73.2% / 7.2%
Childbirths		
% Yes / Mean ±SD (number of childbirths)	59.9 % / 1.3 ± 1.2	71.8 % / 1.5 ± 1.3

* One female with PMS described that she smokes irregularly at the time of inclusion, however the duration was not specified. No males with PMS have smoked irregularly.
** Two females with PMS described that they had been exposed to passive smoking, however, the duration was not specified.
*** Alcohol consumption the week prior to sample collection has been recalculated into volume (cl) consumed 40% alcohol.

Supplementary Table S35. Cox proportional hazard analysis output from confirmed disability worsening analysis in males and females (n = 551). *P* values, coefficients, hazard ratios (HR), and the 95% confidence interval (CI) for each compound. No adjustments were made for multiple comparisons.

	***P* value**	**HR**	**β**	**HR CI (95%) lower**	**HR CI (95%) upper**	**Sex (*P* value)**
**PFBS**	0.102	0.978	-0.022	0.953	1.000	0.321
**PFPeS**	0.435	1.010	0.012	0.982	1.040	0.392
**PFHpA**	0.659	0.988	-0.012	0.938	1.040	0.367
**PFHxS**	0.433	0.960	-0.040	0.868	1.060	0.470
**PFHpS**	0.122	0.923	-0.080	0.834	1.020	0.586
**PFOA**	0.039	0.872	-0.137	0.766	0.993	0.389
**PFOS**	0.043	0.903	-0.102	0.818	0.997	0.555
**FOSA**	0.381	1.060	0.059	0.930	1.210	0.419
**N-MeFOSAA**	0.947	0.996	-0.004	0.898	1.110	0.398
**N-EtFOSAA**	0.858	0.991	-0.009	0.894	1.100	0.398
**PFNA**	0.137	0.909	-0.096	0.801	1.030	0.371
**PFDA**	0.044	0.896	-0.110	0.805	0.997	0.415
**PFDS**	0.927	1.000	0.005	0.912	1.110	0.395
**PFUnA**	0.120	0.922	-0.081	0.832	1.020	0.333
**PFDoA**	0.109	0.891	-0.115	0.774	1.030	0.301
**PFTrDA**	0.909	1.000	0.004	0.934	1.080	0.404
**8:2 FTS**	0.883	0.995	-0.005	0.925	1.070	0.395
**4-OH-CB187**	0.405	0.966	-0.034	0.892	1.050	0.374
**3-OH-CB153**	0.477	0.980	-0.020	0.927	1.040	0.400
**4-OH-CB107**	0.187	0.951	-0.050	0.883	1.020	0.370

The model was adjusted for age, sex, time difference between diagnosis and sample time point, EDSS at time of sample collection, current BMI, undergoing treatment at sample collection, years of regular smoking, years of irregular smoking, years of passive smoking (indoors), number of childbirths, and disease phenotype.

β, beta; *HR*, hazard ratio; *CI*, confidence interval

Supplementary Table S36. Cox proportional hazard analysis output from confirmed disability worsening analysis in males (n = 137). *P* values, coefficients, hazard ratios (HR), and the 95% confidence interval (CI) for each compound. No adjustments were made for multiple comparisons.

	***P* value**	**HR**	**β**	**HR CI (95%) lower**	**HR CI (95%) upper**
**PFBS**	0.194	0.961	-0.040	0.904	1.020
**PFPeS**	0.866	0.995	-0.005	0.935	1.060
**PFHpA**	0.761	1.020	0.016	0.919	1.120
**PFHxS**	0.200	0.901	-0.104	0.768	1.060
**PFHpS**	2.23E-3	0.806	-0.215	0.702	0.926
**PFOA**	0.030	0.795	-0.229	0.647	0.977
**PFOS**	5.08E-4	0.772	-0.259	0.667	0.893
**FOSA**	0.872	0.975	-0.025	0.721	1.320
**N-MeFOSAA**	0.833	1.020	0.022	0.832	1.260
**N-EtFOSAA**	0.547	0.928	-0.074	0.729	1.180
**PFNA**	4.33E-4	0.747	-0.292	0.635	0.879
**PFDA**	0.178	0.880	-0.128	0.731	1.060
**PFDS**	0.317	0.889	-0.118	0.705	1.120
**PFUnA**	0.140	0.889	-0.118	0.760	1.040
**PFDoA**	0.336	0.883	-0.125	0.685	1.140
**PFTrDA**	0.413	0.941	-0.061	0.813	1.090
**8:2 FTS**	0.428	1.070	0.063	0.911	1.250
**4-OH-CB187**	0.144	0.904	-0.101	0.790	1.030
**3-OH-CB153**	0.055	1.110	0.108	0.998	1.240
**4-OH-CB107**	0.914	1.010	0.008	0.868	1.170

The model was adjusted for age, time difference between diagnosis and sample time point, EDSS at time of sample collection, current BMI, undergoing treatment at sample collection, years of regular smoking, years of irregular smoking, years of passive smoking (indoors), and disease phenotype.

β, beta; *HR*, hazard ratio; *CI*, confidence interval

Supplementary Table S37. Cox proportional hazard analysis output from confirmed disability worsening analysis in females (n = 414). *P* values, coefficients, hazard ratios (HR), and the 95% confidence interval (CI) for each compound. No adjustments were made for multiple comparisons.

	***P* value**	**HR**	**β**	**HR CI (95%) lower**	**HR CI (95%) upper**
**PFBS**	0.096	0.976	-0.025	0.948	1.000
**PFPeS**	0.247	1.020	0.020	0.986	1.060
**PFHpA**	0.443	0.977	-0.024	0.920	1.040
**PFHxS**	0.979	1.000	0.002	0.881	1.140
**PFHpS**	0.584	0.962	-0.039	0.837	1.110
**PFOA**	0.166	0.892	-0.115	0.758	1.050
**PFOS**	0.324	0.940	-0.062	0.832	1.060
**FOSA**	0.275	1.090	0.082	0.937	1.260
**N-MeFOSAA**	0.725	1.020	0.022	0.905	1.150
**N-EtFOSAA**	0.762	1.020	0.018	0.908	1.140
**PFNA**	0.812	0.980	-0.020	0.829	1.160
**PFDA**	0.118	0.905	-0.100	0.798	1.030
**PFDS**	0.393	1.050	0.047	0.941	1.170
**PFUnA**	0.173	0.916	-0.088	0.807	1.040
**PFDoA**	0.088	0.864	-0.146	0.730	1.020
**PFTrDA**	0.567	1.020	0.024	0.943	1.110
**8:2 FTS**	0.625	0.980	-0.021	0.902	1.060
**4-OH-CB187**	0.346	0.955	-0.046	0.868	1.050
**3-OH-CB153**	0.246	0.963	-0.038	0.904	1.030
**4-OH-CB107**	0.040	0.914	-0.090	0.838	0.996

The model was adjusted for age, time difference between diagnosis and sample time point, EDSS at time of sample collection, current BMI, undergoing treatment at sample collection, years of regular smoking, years of irregular smoking, years of passive smoking (indoors), number of childbirths, and disease phenotype.

β, beta; *HR*, hazard ratio; *CI*, confidence interval

Supplementary Table S38. Cox proportional hazard analysis output from confirmed disability worsening analysis in males and females (n = 551), adjusting for treatment during the follow-up time. *P* values, coefficients, hazard ratios (HR), and the 95% confidence interval (CI) for each compound. No adjustments were made for multiple comparisons.

	***P* value**	**HR**	**β**	**HR CI (95%) lower**	**HR CI (95%) upper**	**Sex (*P* value)**
**PFBS**	0.205	0.983	-0.017	0.958	1.010	0.547
**PFPeS**	0.095	1.030	0.026	0.996	1.060	0.676
**PFHpA**	0.842	0.995	-0.005	0.943	1.050	0.626
**PFHxS**	0.160	0.929	-0.074	0.837	1.030	0.796
**PFHpS**	0.071	0.910	-0.094	0.821	1.010	0.873
**PFOA**	0.035	0.867	-0.142	0.760	0.990	0.555
**PFOS**	0.053	0.906	-0.099	0.820	1.000	0.812
**FOSA**	0.691	1.030	0.027	0.898	1.180	0.669
**N-MeFOSAA**	0.592	0.971	-0.029	0.873	1.080	0.638
**N-EtFOSAA**	0.488	0.963	-0.037	0.867	1.070	0.638
**PFNA**	0.098	0.893	-0.113	0.782	1.020	0.581
**PFDA**	0.015	0.872	-0.137	0.781	0.974	0.632
**PFDS**	0.610	1.030	0.025	0.931	1.130	0.635
**PFUnA**	0.140	0.923	-0.080	0.830	1.030	0.549
**PFDoA**	0.225	0.913	-0.091	0.789	1.060	0.529
**PFTrDA**	0.949	1.000	0.002	0.931	1.080	0.655
**8:2 FTS**	0.708	0.986	-0.014	0.917	1.060	0.633
**4-OH-CB187**	0.216	0.950	-0.051	0.876	1.030	0.599
**3-OH-CB153**	0.241	0.967	-0.034	0.913	1.020	0.634
**4-OH-CB107**	0.081	0.935	-0.067	0.867	1.010	0.556

The model was adjusted for age, sex, time difference between diagnosis and sample time point, EDSS at time of sample collection, current BMI, undergoing treatment at sample collection, years of regular smoking, years of irregular smoking, years of passive smoking (indoors), number of childbirths, disease phenotype, and treatment during follow-up time.
β, beta; *HR*, hazard ratio; *CI*, confidence interval

Supplementary Table S39. Cox proportional hazard analysis output from confirmed disability worsening analysis in males (n = 137), adjusting for treatment during the follow-up time. *P* values, coefficients, hazard ratios (HR), and the 95% confidence interval (CI) for each compound. No adjustments were made for multiple comparisons.

	***P* value**	**HR**	**β**	**HR CI (95%) lower**	**HR CI (95%) upper**
**PFBS**	0.156	0.957	-0.044	0.900	1.020
**PFPeS**	0.647	1.010	0.014	0.955	1.080
**PFHpA**	0.281	1.060	0.054	0.957	1.160
**PFHxS**	0.205	0.903	-0.102	0.772	1.060
**PFHpS**	5.92e-03	0.824	-0.194	0.718	0.946
**PFOA**	0.092	0.834	-0.181	0.676	1.030
**PFOS**	2.23e-03	0.794	-0.231	0.685	0.921
**FOSA**	0.889	1.020	0.022	0.755	1.380
**N-MeFOSAA**	0.647	1.050	0.047	0.859	1.280
**N-EtFOSAA**	0.563	0.932	-0.071	0.733	1.180
**PFNA**	9.45e-04	0.760	-0.274	0.646	0.894
**PFDA**	0.240	0.893	-0.113	0.739	1.080
**PFDS**	0.471	0.919	-0.085	0.729	1.160
**PFUnA**	0.247	0.910	-0.094	0.776	1.070
**PFDoA**	0.779	0.963	-0.038	0.741	1.250
**PFTrDA**	0.778	0.979	-0.022	0.843	1.140
**8:2 FTS**	0.319	1.080	0.081	0.925	1.270
**4-OH-CB187**	0.091	0.889	-0.118	0.775	1.020
**3-OH-CB153**	0.027	1.130	0.124	1.010	1.260
**4-OH-CB107**	0.672	1.030	0.032	0.891	1.200

The model was adjusted for age, time difference between diagnosis and sample time point, EDSS at time of sample collection, current BMI, undergoing treatment at sample collection, years of regular smoking, years of irregular smoking, years of passive smoking (indoors), disease phenotype, and treatment during follow-up time.
β, beta; *HR*, hazard ratio; *CI*, confidence interval

Supplementary Table S40. Cox proportional hazard analysis output from confirmed disability worsening analysis in females (n = 414), adjusting for treatment during the follow-up time. *P* values, coefficients, hazard ratios (HR), and the 95% confidence interval (CI) for each compound. No adjustments were made for multiple comparisons.

	***P* value**	**HR**	**β**	**HR CI (95%) lower**	**HR CI (95%) upper**
**PFBS**	0.181	0.980	-0.020	0.952	1.010
**PFPeS**	0.080	1.030	0.031	0.996	1.070
**PFHpA**	0.261	0.965	-0.035	0.908	1.030
**PFHxS**	0.293	0.930	-0.073	0.811	1.060
**PFHpS**	0.147	0.901	-0.104	0.783	1.040
**PFOA**	0.019	0.820	-0.199	0.695	0.967
**PFOS**	0.258	0.931	-0.071	0.824	1.050
**FOSA**	0.761	1.020	0.024	0.880	1.190
**N-MeFOSAA**	0.491	0.955	-0.046	0.838	1.090
**N-EtFOSAA**	0.590	0.968	-0.033	0.859	1.090
**PFNA**	0.309	0.911	-0.093	0.762	1.090
**PFDA**	0.019	0.855	-0.157	0.750	0.975
**PFDS**	0.287	1.060	0.059	0.951	1.180
**PFUnA**	0.111	0.901	-0.105	0.792	1.020
**PFDoA**	0.074	0.855	-0.157	0.720	1.020
**PFTrDA**	0.868	1.010	0.0072	0.925	1.100
**8:2 FTS**	0.301	0.956	-0.045	0.879	1.040
**4-OH-CB187**	0.092	0.921	-0.082	0.837	1.010
**3-OH-CB153**	0.050	0.936	-0.066	0.876	1.000
**4-OH-CB107**	0.003	0.871	-0.139	0.796	0.953

The model was adjusted for age, time difference between diagnosis and sample time point, EDSS at time of sample collection, current BMI, undergoing treatment at sample collection, years of regular smoking, years of irregular smoking, years of passive smoking (indoors), number of childbirths, disease phenotype, and treatment during follow-up time.

β, beta; *HR*, hazard ratio; *CI*, confidence interval

Supplementary Table S41. Cox proportional hazard analysis for serum albumin concentrations association with confirmed disability worsening. Analysis were performed in males and females together (n = 551), as well as separately (males n = 137, females n = 414). *P* values, coefficients, hazard ratios (HR), and the 95% confidence interval (CI).

	***P* value**	**HR**	**β**	**HR CI (95%) lower**	**HR CI (95%) upper**
**Males and Females**	0.371	1.350	0.297	0.702	2.580
**Males**	0.413	1.920	0.651	0.404	9.120
**Females**	0.621	1.200	0.181	0.584	2.460

The model was adjusted for age, sex, time difference between diagnosis and sample time point, EDSS at time of sample collection, current BMI, undergoing treatment at sample collection, years of regular smoking, years of irregular smoking, years of passive smoking (indoors), number of childbirths, and disease phenotype. Sex was excluded for the sex-specific models. In the male model, the number of childbirths was also excluded as a covariate.

β, beta; *HR*, hazard ratio; *CI*, confidence interval

Supplementary Table S42. Cox proportional hazard analysis output from confirmed disability worsening analysis in males and females (n = 551), adjusting for serum albumin concentrations. *P* values, coefficients, hazard ratios (HR), and the 95% confidence interval (CI) for each compound. No adjustments were made for multiple comparisons.

	***P* value**	**HR**	**β**	**HR CI (95%) lower**	**HR CI (95%) upper**	**Sex (*P* value)**
**PFBS**	0.073	0.976	-0.024	0.951	1.000	0.248
**PFPeS**	0.429	1.010	0.012	0.983	1.040	0.335
**PFHpA**	0.640	0.988	-0.012	0.938	1.040	0.310
**PFHxS**	0.418	0.959	-0.042	0.868	1.060	0.403
**PFHpS**	0.118	0.923	-0.080	0.835	1.020	0.508
**PFOA**	0.041	0.874	-0.135	0.768	0.994	0.333
**PFOS**	0.043	0.903	-0.101	0.819	0.997	0.482
**FOSA**	0.384	1.060	0.058	0.930	1.210	0.358
**N-MeFOSAA**	0.957	0.997	-0.003	0.900	1.110	0.338
**N-EtFOSAA**	0.851	0.990	-0.010	0.894	1.100	0.339
**PFNA**	0.143	0.911	-0.094	0.803	1.030	0.317
**PFDA**	0.047	0.897	-0.108	0.807	0.999	0.356
**PFDS**	0.867	1.010	0.008	0.916	1.110	0.333
**PFUnA**	0.125	0.923	-0.080	0.833	1.020	0.280
**PFDoA**	0.125	0.896	-0.110	0.778	1.030	0.262
**PFTrDA**	0.906	1.000	0.004	0.934	1.080	0.345
**8:2 FTS**	0.999	1.000	3.03e-05	0.93	1.080	0.338
**4-OH-CB187**	0.405	0.967	-0.034	0.892	1.050	0.317
**3-OH-CB153**	0.452	0.979	-0.021	0.926	1.030	0.337
**4-OH-CB107**	0.144	0.946	-0.056	0.878	1.020	0.298

The model was adjusted for age, sex, time difference between diagnosis and sample time point, EDSS at time of sample collection, current BMI, undergoing treatment at sample collection, years of regular smoking, years of irregular smoking, years of passive smoking (indoors), number of childbirths, disease phenotype, and serum albumin concentrations.

β, beta; *HR*, hazard ratio; *CI*, confidence interval

Supplementary Table S43. Cox proportional hazard analysis output from confirmed disability worsening analysis in males (n = 137), adjusting for serum albumin concentrations. *P* values, coefficients, hazard ratios (HR), and the 95% confidence interval (CI) for each compound. No adjustments were made for multiple comparisons.

	***P* value**	**HR**	**β**	**HR CI (95%) lower**	**HR CI (95%) upper**
**PFBS**	0.151	0.957	-0.044	0.901	1.020
**PFPeS**	0.825	0.993	-0.007	0.934	1.060
**PFHpA**	0.834	1.010	0.011	0.915	1.120
**PFHxS**	0.219	0.905	-0.100	0.771	1.060
**PFHpS**	3.01e-03	0.812	-0.209	0.707	0.932
**PFOA**	0.0362	0.803	-0.220	0.654	0.986
**PFOS**	6.67e-04	0.776	-0.254	0.670	0.898
**FOSA**	0.830	0.968	-0.033	0.717	1.310
**N-MeFOSAA**	0.785	1.030	0.029	0.836	1.270
**N-EtFOSAA**	0.598	0.937	-0.065	0.737	1.190
**PFNA**	6.04e-04	0.752	-0.285	0.639	0.885
**PFDA**	0.191	0.883	-0.124	0.733	1.060
**PFDS**	0.368	0.899	-0.107	0.713	1.130
**PFUnA**	0.182	0.899	-0.106	0.770	1.050
**PFDoA**	0.377	0.893	-0.113	0.694	1.150
**PFTrDA**	0.486	0.950	-0.052	0.821	1.100
**8:2 FTS**	0.294	1.090	0.083	0.930	1.270
**4-OH-CB187**	0.198	0.915	-0.089	0.800	1.050
**3-OH-CB153**	0.035	1.130	0.120	1.010	1.260
**4-OH-CB107**	0.910	1.010	0.009	0.868	1.170

The model was adjusted for age, time difference between diagnosis and sample time point, EDSS at time of sample collection, current BMI, undergoing treatment at sample collection, years of regular smoking, years of irregular smoking, years of passive smoking (indoors), disease phenotype, and serum albumin concentrations.
β, beta; *HR*, hazard ratio; *CI*, confidence interval

Supplementary Table S44. Cox proportional hazard analysis output from confirmed disability worsening analysis in females (n = 414), adjusting for serum albumin concentrations. *P* values, coefficients, hazard ratios (HR), and the 95% confidence interval (CI) for each compound. No adjustments were made for multiple comparisons.

	***P* value**	**HR**	**β**	**HR CI (95%) lower**	**HR CI (95%) upper**
**PFBS**	0.084	0.975	-0.026	0.947	1.000
**PFPeS**	0.249	1.020	0.020	0.986	1.060
**PFHpA**	0.448	0.977	-0.023	0.920	1.040
**PFHxS**	0.999	1.000	8.82e-05	0.879	1.140
**PFHpS**	0.560	0.960	-0.041	0.836	1.100
**PFOA**	0.164	0.892	-0.115	0.759	1.050
**PFOS**	0.315	0.939	-0.063	0.831	1.060
**FOSA**	0.275	1.090	0.082	0.937	1.260
**N-MeFOSAA**	0.738	1.020	0.021	0.904	1.150
**N-EtFOSAA**	0.777	1.020	0.017	0.907	1.140
**PFNA**	0.816	0.980	-0.020	0.829	1.160
**PFDA**	0.121	0.905	-0.100	0.798	1.030
**PFDS**	0.384	1.050	0.048	0.942	1.170
**PFUnA**	0.170	0.915	-0.089	0.806	1.040
**PFDoA**	0.095	0.866	-0.143	0.732	1.030
**PFTrDA**	0.576	1.020	0.024	0.942	1.110
**8:2 FTS**	0.672	0.982	-0.018	0.903	1.070
**4-OH-CB187**	0.354	0.956	-0.045	0.868	1.050
**3-OH-CB153**	0.219	0.961	-0.040	0.901	1.020
**4-OH-CB107**	0.032	0.910	-0.095	0.834	0.992

The model was adjusted for age, time difference between diagnosis and sample time point, EDSS at time of sample collection, current BMI, undergoing treatment at sample collection, years of regular smoking, years of irregular smoking, years of passive smoking (indoors), number of childbirths, disease phenotype, and serum albumin concentrations.
β, beta; *HR*, hazard ratio; *CI*, confidence interval

Supplementary Table S45. Cox proportional hazard analysis output from relapsing-remitting multiple sclerosis (RRMS) to secondary-progressive multiple sclerosis (SPMS) transition analysis (n = 801). *P* values, coefficients, hazard ratios (HR), and the 95% confidence interval (CI) for each compound. No adjustments were made for multiple comparisons.

	***P* value**	**HR**	**β**	**HR CI (95%) lower**	**HR CI (95%) upper**
**PFBS**	0.808	0.995	-0.005	0.959	1.030
**PFPeS**	0.753	1.010	0.008	0.961	1.060
**PFHpA**	0.234	0.958	-0.043	0.893	1.030
**PFHxS**	0.683	1.030	0.034	0.879	1.220
**PFHpS**	0.222	1.120	0.115	0.933	1.350
**PFOA**	0.938	0.992	-0.009	0.800	1.230
**PFOS**	0.331	1.090	0.090	0.913	1.310
**FOSA**	0.300	0.905	-0.100	0.749	1.090
**N-MeFOSAA**	0.789	1.020	0.021	0.874	1.190
**N-EtFOSAA**	0.697	1.030	0.030	0.886	1.200
**PFNA**	0.610	0.951	-0.050	0.785	1.150
**PFDA**	0.712	0.967	-0.033	0.810	1.150
**PFDS**	0.515	0.951	-0.051	0.817	1.110
**PFUnA**	0.364	0.930	-0.073	0.795	1.090
**PFDoA**	0.051	0.813	-0.207	0.660	1.000
**PFTrDA**	0.972	0.998	-0.002	0.899	1.110
**8:2 FTS**	0.622	1.030	0.027	0.923	1.140
**4-OH-CB187**	0.369	1.070	0.069	0.922	1.240
**3-OH-CB153**	0.523	0.975	-0.026	0.900	1.050
**4-OH-CB107**	0.850	0.990	-0.010	0.893	1.100

The model was adjusted for age, sex, time difference between diagnosis and sample time point, current BMI, treatment at sample collection, years of regular smoking, years of irregular smoking, years of indoor passive smoking, and number of childbirths.
β, beta; *HR*, hazard ratio; *CI*, confidence interval

Supplementary Table S46. Cox proportional hazard analysis output from relapsing-remitting multiple sclerosis (RRMS) to secondary-progressive multiple sclerosis (SPMS) transition analysis (n = 801), adjusting for treatment during the follow-up time. *P* values, coefficients, hazard ratios (HR), and the 95% confidence interval (CI) for each compound. No adjustments were made for multiple comparisons.

	***P* value**	**HR**	**β**	**HR CI (95%) lower**	**HR CI (95%) upper**
**PFBS**	0.625	1.010	0.009	0.972	1.050
**PFPeS**	0.588	1.010	0.013	0.967	1.060
**PFHpA**	0.319	0.963	-0.038	0.893	1.040
**PFHxS**	0.651	0.964	-0.037	0.820	1.130
**PFHpS**	0.384	1.090	0.082	0.903	1.300
**PFOA**	0.775	0.969	-0.032	0.780	1.200
**PFOS**	0.426	1.080	0.076	0.895	1.300
**FOSA**	0.475	0.929	-0.073	0.760	1.140
**N-MeFOSAA**	0.381	1.070	0.072	0.915	1.260
**N-EtFOSAA**	0.525	1.050	0.052	0.898	1.230
**PFNA**	0.536	0.937	-0.065	0.762	1.150
**PFDA**	0.704	0.965	-0.036	0.803	1.160
**PFDS**	0.435	0.939	-0.063	0.801	1.100
**PFUnA**	0.440	0.938	-0.065	0.796	1.100
**PFDoA**	0.092	0.818	-0.201	0.647	1.030
**PFTrDA**	0.944	1.000	0.004	0.898	1.120
**8:2 FTS**	0.432	1.040	0.043	0.937	1.160
**4-OH-CB187**	0.270	1.090	0.089	0.933	1.280
**3-OH-CB153**	0.792	0.990	-0.010	0.916	1.070
**4-OH-CB107**	0.989	0.999	-7.49e-04	0.901	1.110

The model was adjusted for age, sex, time difference between diagnosis and sample time point, current BMI, treatment at sample collection, years of regular smoking, years of irregular smoking, years of indoor passive smoking, number of childbirths, and treatment during the follow-up time.

β, beta; *HR*, hazard ratio; *CI*, confidence interval

Supplementary Table S47. Cox proportional hazard analysis output from relapsing-remitting multiple sclerosis (RRMS) to secondary-progressive multiple sclerosis (SPMS) transition analysis (n = 801), adjusting for serum albumin concentrations. *P* values, coefficients, hazard ratios (HR), and the 95% confidence interval (CI) for each compound. No adjustments were made for multiple comparisons.

	***P* value**	**HR**	**β**	**HR CI (95%) lower**	**HR CI (95%) upper**
**PFBS**	0.956	0.999	-0.001	0.962	1.040
**PFPeS**	0.722	1.010	0.009	0.962	1.060
**PFHpA**	0.217	0.956	-0.045	0.890	1.030
**PFHxS**	0.661	1.040	0.037	0.881	1.220
**PFHpS**	0.188	1.130	0.125	0.941	1.370
**PFOA**	0.987	0.998	-0.002	0.803	1.240
**PFOS**	0.285	1.100	0.099	0.921	1.320
**FOSA**	0.292	0.903	-0.102	0.746	1.090
**N-MeFOSAA**	0.768	1.020	0.024	0.875	1.200
**N-EtFOSAA**	0.630	1.040	0.037	0.892	1.210
**PFNA**	0.635	0.954	-0.047	0.786	1.160
**PFDA**	0.723	0.968	-0.032	0.811	1.160
**PFDS**	0.484	0.947	-0.054	0.814	1.100
**PFUnA**	0.365	0.930	-0.072	0.796	1.090
**PFDoA**	0.048	0.809	-0.212	0.655	0.999
**PFTrDA**	0.970	0.998	-0.002	0.900	1.110
**8:2 FTS**	0.723	1.020	0.020	0.916	1.140
**4-OH-CB187**	0.381	1.070	0.068	0.919	1.250
**3-OH-CB153**	0.607	0.979	-0.021	0.905	1.060
**4-OH-CB107**	0.904	0.994	-0.006	0.895	1.100

The model was adjusted for age, sex, time difference between diagnosis and sample time point, current BMI, treatment at sample collection, years of regular smoking, years of irregular smoking, years of indoor passive smoking, number of childbirths, and serum albumin concentrations.

β, beta; HR, hazard ratio; CI, confidence interval

Supplementary Table S48. Per- and polyfluorinated substances (PFAS) and hydroxyl polychlorinated biphenyls (OH-PCBs) two-tailed Spearman rank correlation test with 25(OH)D3. *P* values and correlation coefficients (r) for each correlation.

	Males and females		Males and females				Females				Males			
	HC and MS		HC		MS		HC		MS		HC		MS	
	***P* value**	**r**	***P* value**	**r**	***P* value**	**r**	***P* value**	**r**	***P* value**	**r**	***P* value**	**r**	***P* value**	**r**
**PFBS**	0.005	-0.070	0.079	-0.062	0.029	-0.078	0.244	-0.047	0.016	-0.099	0.074	-0.127	0.073	-0.126
**PFPeS**	1.36e-21	-0.235	1.60e-14	-0.265	3.20e-09	-0.209	5.40e-12	-0.273	9.25e-11	-0.263	8.98e-05	-0.274	0.144	-0.103
**PFHpA**	3.85e-05	0.103	5.41e-04	0.121	0.026	0.079	2.10e-04	0.149	0.030	0.090	0.450	0.054	0.749	0.023
**PFHxS**	0.231	-0.030	0.578	-0.020	0.281	-0.038	0.799	0.010	0.970	0.002	0.499	0.048	0.624	0.035
**PFHpS**	0.537	-0.015	0.748	-0.011	0.683	-0.015	0.398	0.0341	0.300	0.043	0.554	0.042	0.226	0.085
**PFOA**	0.029	0.055	0.046	0.070	0.211	0.045	0.010	0.104	0.120	0.064	0.336	0.069	0.385	0.061
**PFOS**	0.021	-0.058	0.076	-0.062	0.183	-0.047	0.262	-0.045	0.574	-0.023	0.534	-0.044	0.952	-0.004
**FOSA**	0.771	0.007	0.999	-3.22e-05	0.654	0.016	0.801	-0.010	0.364	0.038	0.491	0.049	0.910	-0.008
**N-MeFOSAA**	0.370	0.022	0.665	0.015	0.357	0.033	0.959	-0.002	0.575	0.023	0.271	0.079	0.425	0.056
**N-EtFOSAA**	0.008	-0.066	0.016	-0.085	0.180	-0.048	0.007	-0.109	0.224	-0.050	0.634	-0.034	1.88e-01	-0.093
**PFNA**	1.09e-08	0.142	2.51e-05	0.147	8.28e-05	0.139	4.09e-05	0.165	1.85e-04	0.154	0.044	0.143	0.231	0.085
**PFDA**	4.35e-04	0.088	0.071	0.063	0.001	0.116	0.158	0.057	0.004	0.119	0.098	0.118	0.088	0.120
**PFDS**	0.775	-0.007	0.808	-0.009	0.877	-0.006	0.581	-0.022	0.029	-0.090	0.823	-0.016	0.018	-0.095
**PFUnA**	0.011	0.063	0.582	0.019	0.002	0.110	0.483	0.028	0.008	0.110	0.616	0.036	0.448	0.054
**PFDoA**	4.86e-04	0.087	0.237	0.042	1.50e-04	0.134	0.323	0.040	0.007	0.112	0.511	0.047	0.004	0.199
**PFTrDA**	0.261	-0.028	0.106	-0.057	0.995	-2.11e-04	0.083	-0.070	0.980	-0.001	0.745	-0.023	0.592	-0.038
**8:2 FTS**	8.55e-11	-0.161	1.76e-06	-0.167	1.71e-05	-0.152	3.75e-05	-0.165	4.86e-04	-0.143	0.029	-0.155	1.87e-04	-0.259
**4-OH-CB187**	2.85e-08	-0.138	0.001	-0.112	8.40e-06	-0.158	0.005	-0.113	5.34e-06	-0.186	0.709	-0.027	0.026	-0.157
**3-OH-CB153**	0.382	-0.022	0.683	0.014	0.015	-0.051	0.348	0.038	0.484	-0.029	0.077	0.126	0.348	0.066
**4-OH-CB107**	8.43e-07	-0.123	5.46e-04	-0.121	4.09e-04	-0.125	0.002	-0.124	0.003	-0.120	0.922	0.007	0.335	-0.068

Supplementary Table S49. Cox proportional hazard analysis for 25(OH)D3 serum levels association with confirmed disability worsening. *P* values, coefficients, hazard ratios (HR), and the 95% confidence interval (CI). Analysis were performed in males and females together (n = 551), as well as separately (males n = 137, females n = 414).

	***P* value**	**HR**	**β**	**HR CI (95%) lower**	**HR CI (95%) upper**
**Males and Females**	0.040	0.880	-0.128	0.779	0.994
**Males**	0.878	0.979	-0.021	0.747	1.280
**Females**	0.045	0.873	-0.135	0.765	0.997

The model was adjusted for age, sex, the time difference between diagnosis and sample time point, EDSS at the time of sample collection, current BMI, treatment at sample collection, years of regular smoking, years of irregular smoking, years of passive smoking (indoor), number of childbirths, and the month blood samples were collected. Sex was excluded for the sex-specific models. In the male model, the number of childbirths was also excluded as a covariate.
β, beta; *HR*, hazard ratio; *CI*, confidence interval

Supplementary Table S50. Cox proportional hazard analysis for per- and polyfluorinated substances (PFAS) and hydroxyl polychlorinated biphenyls (OH-PCBs) association with confirmed disability worsening adjusting for 25(OH)D3 levels (n = 551). Analysis based on the entire group. *P* values, coefficients, hazard ratios (HR), and the 95% confidence interval (CI). No adjustments were made for multiple comparisons.

	***P* value**	**HR**	**β**	**HR CI (95%) lower**	**HR CI (95%) upper**
**PFBS**	0.039	0.971	-0.030	0.944	0.999
**PFPeS**	0.859	1.003	0.003	0.971	1.036
**PFHpA**	0.679	0.988	-0.012	0.935	1.045
**PFHxS**	0.350	0.947	-0.055	0.845	1.062
**PFHpS**	0.128	0.918	-0.086	0.822	1.025
**PFOA**	0.022	0.848	-0.165	0.736	0.976
**PFOS**	0.024	0.881	-0.127	0.789	0.983
**FOSA**	0.794	1.019	0.019	0.883	1.177
**N-MeFOSAA**	0.851	0.989	-0.011	0.883	1.108
**N-EtFOSAA**	0.829	0.988	-0.012	0.883	1.105
**PFNA**	0.047	0.864	-0.146	0.748	0.998
**PFDA**	0.041	0.886	-0.121	0.789	0.995
**PFDS**	0.625	0.973	-0.027	0.873	1.085
**PFUnA**	0.077	0.908	-0.096	0.817	1.010
**PFDoA**	0.023	0.847	-0.166	0.734	0.977
**PFTrDA**	0.419	0.968	-0.032	0.895	1.047
**8:2 FTS**	0.579	0.979	-0.022	0.906	1.056
**4-OH-CB187**	0.605	0.977	-0.023	0.894	1.068
**3-OH-CB153**	0.330	0.970	-0.030	0.913	1.031
**4-OH-CB107**	0.093	0.934	-0.068	0.863	1.011

The model was adjusted for age, sex, the time difference between diagnosis and sample time point, EDSS at the time of sample collection, current BMI, treatment at sample collection, years of regular smoking, years of irregular smoking, years of passive smoking (indoor), number of childbirths, the month blood samples were collected, and 25(OH)D3 levels.
β, beta; *HR*, hazard ratio; *CI*, confidence interval

Supplementary Table S51. Cox proportional hazard analysis for per- and polyfluorinated substances (PFAS) and hydroxyl polychlorinated biphenyls (OH-PCBs) association with confirmed disability worsening in males (n = 137), adjusting for 25(OH)D3 levels. *P* values, coefficients, hazard ratios (HR), and the 95% confidence interval (CI). No adjustments were made for multiple comparisons.

	***P* value**	**HR**	**β**	**HR CI (95%) lower**	**HR CI (95%) upper**
**PFBS**	0.130	0.951	-0.050	0.892	1.015
**PFPeS**	0.612	0.983	-0.018	0.918	1.052
**PFHpA**	0.324	0.949	-0.053	0.855	1.053
**PFHxS**	0.160	0.879	-0.129	0.734	1.052
**PFHpS**	0.012	0.832	-0.183	0.721	0.961
**PFOA**	0.014	0.777	-0.252	0.635	0.951
**PFOS**	0.024	0.832	-0.184	0.709	0.976
**FOSA**	0.918	0.983	-0.017	0.710	1.360
**N-MeFOSAA**	0.848	1.021	0.021	0.823	1.267
**N-EtFOSAA**	0.934	1.011	0.011	0.777	1.317
**PFNA**	0.019	0.811	-0.210	0.680	0.967
**PFDA**	0.215	0.879	-0.128	0.718	1.077
**PFDS**	0.091	0.817	-0.202	0.646	1.033
**PFUnA**	0.089	0.876	-0.132	0.752	1.021
**PFDoA**	0.257	0.869	-0.140	0.682	1.107
**PFTrDA**	0.158	0.896	-0.110	0.769	1.043
**8:2 FTS**	0.711	1.031	0.031	0.875	1.215
**4-OH-CB187**	0.724	0.973	-0.027	0.838	1.131
**3-OH-CB153**	0.036	1.142	0.132	1.009	1.292
**4-OH-CB107**	0.774	0.975	-0.025	0.823	1.156

The model was adjusted for age, the time difference between diagnosis and sample time point, EDSS at the time of sample collection, current BMI, treatment at sample collection, years of regular smoking, years of irregular smoking, years of passive smoking (indoor), the month blood samples were collected, and 25(OH)D3 levels.

β, beta; *HR*, hazard ratio; *CI*, confidence interval

Supplementary Table S52. Cox proportional hazard analysis for per- and polyfluorinated substances (PFAS) and hydroxyl polychlorinated biphenyls (OH-PCBs) association with confirmed disability worsening in females (n = 414), adjusting for 25(OH)D3 levels. *P* values, coefficients, hazard ratios (HR), and the 95% confidence interval (CI). No adjustments were made for multiple comparisons.

	***P* value**	**HR**	**β**	**HR CI (95%) lower**	**HR CI (95%) upper**
**PFBS**	0.050	0.969	-0.031	0.939	1.000
**PFPeS**	0.702	1.007	0.007	0.970	1.046
**PFHpA**	0.759	0.990	-0.010	0.929	1.056
**PFHxS**	0.867	0.988	-0.012	0.859	1.136
**PFHpS**	0.635	0.964	-0.037	0.827	1.123
**PFOA**	0.113	0.867	-0.143	0.726	1.035
**PFOS**	0.101	0.892	-0.114	0.779	1.022
**FOSA**	0.715	1.030	0.030	0.878	1.208
**N-MeFOSAA**	0.839	1.014	0.014	0.885	1.162
**N-EtFOSAA**	0.977	1.002	0.002	0.884	1.135
**PFNA**	0.225	0.884	-0.124	0.724	1.079
**PFDA**	0.127	0.901	-0.104	0.789	1.030
**PFDS**	0.893	1.009	0.009	0.891	1.142
**PFUnA**	0.162	0.911	-0.093	0.799	1.038
**PFDoA**	0.014	0.811	-0.210	0.685	0.959
**PFTrDA**	0.728	0.984	-0.016	0.900	1.076
**8:2 FTS**	0.467	0.968	-0.032	0.887	1.056
**4-OH-CB187**	0.466	0.961	-0.040	0.864	1.070
**3-OH-CB153**	0.137	0.949	-0.052	0.887	1.017
**4-OH-CB107**	0.010	0.887	-0.120	0.810	0.972

The model was adjusted for age, the time difference between diagnosis and sample time point, EDSS at the time of sample collection, current BMI, treatment at sample collection, years of regular smoking, years of irregular smoking, years of passive smoking (indoor), number of childbirths, the month blood samples were collected, and 25(OH)D3 levels.

β, beta; *HR*, hazard ratio; *CI*, confidence interval

Supplementary Table S53. Cox proportional hazard analysis for 25(OH)D3 serum levels association with the transition from relapsing-remitting multiple sclerosis (RRMS) to secondary-progressive multiple sclerosis (SPMS) (n = 801). *P* values, coefficients, hazard ratios (HR), and the 95% confidence interval (CI).

	***P* value**	**HR**	**β**	**HR CI (95%) lower**	**HR CI (95%) upper**
**Males and Females**	0.221	0.898	-0.107	0.757	1.070

The model was adjusted for age, sex, the time difference between diagnosis and sample time point, EDSS at the time of sample collection, current BMI, treatment at sample collection, years of regular smoking, years of irregular smoking, years of passive smoking (indoor), number of childbirths, and the month blood samples were collected.
β, beta; HR, hazard ratio; CI, confidence interval

Supplementary Table S54. Cox proportional hazard analysis for per- and polyfluorinated substances (PFAS) and hydroxyl polychlorinated biphenyls (OH-PCBs) association with the transition from relapsing-remitting multiple sclerosis (RRMS) to secondary-progressive multiple sclerosis (SPMS) (n = 801), adjusting for 25(OH)D3 levels. *P* values, coefficients, hazard ratios (HR), and the 95% confidence interval (CI). No adjustments were made for multiple comparisons.

	***P* value**	**HR**	**β**	**HR CI (95%) lower**	**HR CI (95%) upper**
**PFBS**	0.327	0.979	-0.021	0.939	1.021
**PFPeS**	0.761	1.008	0.008	0.957	1.061
**PFHpA**	0.212	0.951	-0.050	0.879	1.029
**PFHxS**	0.547	1.057	0.055	0.883	1.266
**PFHpS**	0.143	1.175	0.161	0.947	1.458
**PFOA**	0.762	1.038	0.037	0.816	1.320
**PFOS**	0.366	1.097	0.093	0.897	1.341
**FOSA**	0.882	0.984	-0.016	0.800	1.211
**N-MeFOSAA**	0.733	1.030	0.029	0.870	1.219
**N-EtFOSAA**	0.559	1.050	0.049	0.891	1.237
**PFNA**	0.88	1.018	0.018	0.810	1.279
**PFDA**	0.533	1.068	0.066	0.868	1.314
**PFDS**	0.409	0.933	-0.069	0.791	1.100
**PFUnA**	0.464	0.939	-0.063	0.793	1.111
**PFDoA**	0.076	0.817	-0.202	0.654	1.022
**PFTrDA**	0.784	1.017	0.017	0.903	1.145
**8:2 FTS**	0.724	0.980	-0.020	0.874	1.098
**4-OH-CB187**	0.356	1.086	0.083	0.912	1.294
**3-OH-CB153**	0.826	1.010	0.010	0.926	1.101
**4-OH-CB107**	0.822	1.013	0.013	0.906	1.133

The model was adjusted for age, sex, the time difference between diagnosis and sample time point, EDSS at the time of sample collection, current BMI, treatment at sample collection, years of regular smoking, years of irregular smoking, years of passive smoking (indoor), number of childbirths, the month blood samples were collected, and 25(OH)D3 levels.
β, beta; *HR*, hazard ratio; *CI*, confidence interval

Supplementary Table S55. *P* values for per- and polyfluorinated substances (PFAS) and hydroxyl polychlorinated biphenyls (OH-PCBs) correlations. Two-tailed Spearman's rank correlation test analysis on controls and persons with MS.

	**PFBS**	**PFPeS**	**PFHpA**	**PFHxS**	**PFHpS**	**PFOA**	**PFOS**	**FOSA**	**N-MeFOSAA**	**N-EtFOSAA**	**PFNA**	**PFDA**	**PFDS**	**PFUnA**	**PFDoA**	**PFTrDA**	**8:2 FTS**	**4-OH-CB187**	**3-OH-CB153**	**4-OH-CB107**
**PFBS**	-	1.21e-10	6.94e-10	4.81e-02	5.21e-01	1.09e-01	9.79e-01	2.00e-02	9.48e-01	5.24e-03	2.74e-01	2.33e-01	7.11e-02	2.47e-01	2.76e-01	3.83e-03	5.91e-01	3.63e-01	7.40e-06	5.67e-03
**PFPeS**	1.21e-10	-	4.40e-82	5.61e-91	6.63e-26	4.35e-56	1.77e-26	1.38e-03	2.68e-07	1.47e-04	1.15e-29	3.94e-22	2.76e-06	7.42e-18	5.38e-17	4.44e-16	1.59e-05	4.65e-10	4.15e-03	6.99e-09
**PFHpA**	6.94e-10	4.40e-82	-	1.36e-35	2.67e-23	3.82e-110	1.08e-33	3.42e-21	2.22e-16	9.42e-12	1.98e-58	9.71e-53	5.05e-04	1.54e-34	5.72e-42	2.24e-26	4.58e-18	2.00e-15	2.03e-06	2.01e-11
**PFHxS**	4.81e-02	5.61e-91	1.36e-35	-	0.00	3.69e-201	1.11e-235	4.54e-36	3.90e-30	7.59e-14	8.36e-122	7.86e-127	8.93e-07	6.71e-66	4.67e-42	6.06e-32	8.88e-15	2.01e-64	6.21e-18	3.82e-27
**PFHpS**	5.21e-01	6.63e-26	2.67e-23	0.00	-	6.66e-272	0.00	3.19e-46	1.75e-57	1.87e-21	3.30e-159	5.67e-129	1.08e-06	3.13e-59	7.04e-46	4.12e-26	9.08e-18	8.41e-92	2.98e-23	1.48e-29
**PFOA**	1.09e-01	4.35e-56	3.82e-110	3.69e-201	6.66e-272	-	3.09e-249	1.45e-42	3.65e-57	9.24e-32	1.04e-242	3.97e-180	3.23e-04	1.10e-81	2.91e-77	3.72e-49	1.81e-36	5.58e-84	2.28e-27	1.62e-38
**PFOS**	9.79e-01	1.77e-26	1.08e-33	1.11e-235	0.00	3.09e-249	-	2.65e-78	4.31e-91	1.84e-34	2.09e-281	4.51e-275	2.22e-16	7.43e-192	2.82e-154	4.60e-120	1.20e-35	9.55e-131	9.21e-44	6.76e-60
**FOSA**	2.00e-02	1.38e-03	3.42e-21	4.54e-36	3.19e-46	1.45e-42	2.65e-78	-	8.38e-101	1.43e-55	6.60e-42	3.37e-72	2.89e-09	2.80e-67	1.78e-64	7.14e-54	3.82e-31	6.26e-41	1.28e-32	1.67e-31
**N-MeFOSAA**	9.48e-01	2.68e-07	2.22e-16	3.90e-30	1.75e-57	3.65e-57	4.31e-91	8.38e-101	-	2.06e-75	1.18e-42	2.36e-37	6.11e-11	1.01e-22	3.36e-29	8.55e-24	6.32e-40	3.76e-56	2.91e-19	1.59e-30
**N-EtFOSAA**	5.24e-03	1.47e-04	9.42e-12	7.59e-14	1.87e-21	9.24e-32	1.84e-34	1.43e-55	2.06e-75	-	1.45e-13	1.23e-17	3.73e-07	3.38e-10	3.40e-10	3.26e-09	6.92e-27	2.22e-16	1.36e-08	1.03e-16
**PFNA**	2.74e-01	1.15e-29	1.98e-58	8.36e-122	3.30e-159	1.04e-242	2.09e-281	6.60e-42	1.18e-42	1.45e-13	-	0.00	5.14e-09	1.62e-235	1.86e-220	6.26e-151	7.64e-20	4.49e-99	2.94e-31	4.18e-52
**PFDA**	2.33e-01	3.94e-22	9.71e-53	7.86e-127	5.67e-129	3.97e-180	4.51e-275	3.37e-72	2.36e-37	1.23e-17	0.00	-	3.02e-19	0.00	0.00	2.84e-213	2.12e-49	3.08e-72	2.40e-31	1.76e-49
**PFDS**	7.11e-02	2.76e-06	5.05e-04	8.93e-07	1.08e-06	3.23e-04	2.22e-16	2.89e-09	6.11e-11	3.73e-07	5.14e-09	3.02e-19	-	1.91e-25	2.61e-36	1.32e-22	1.45e-06	2.05e-02	2.63e-01	5.10e-06
**PFUnA**	2.47e-01	7.42e-18	1.54e-34	6.71e-66	3.13e-59	1.10e-81	7.43e-192	2.80e-67	1.01e-22	3.38e-10	1.62e-235	0.00	1.91e-25	-	0.00	0.00	8.48e-25	5.73e-68	2.88e-45	2.34e-59
**PFDoA**	2.76e-01	5.38e-17	5.72e-42	4.67e-42	7.04e-46	2.91e-77	2.82e-154	1.78e-64	3.36e-29	3.40e-10	1.86e-220	0.00	2.61e-36	0.00	-	0.00	1.46e-29	5.85e-53	1.86e-29	8.74e-45
**PFTrDA**	3.83e-03	4.44e-16	2.24e-26	6.06e-32	4.12e-26	3.72e-49	4.60e-120	7.14e-54	8.55e-24	3.26e-09	6.26e-151	2.84e-213	1.32e-22	0.00	0.00	-	1.29e-19	1.18e-58	5.34e-41	6.30e-52
**8:2 FTS**	5.91e-01	1.59e-05	4.58e-18	8.88e-15	9.08e-18	1.81e-36	1.20e-35	3.82e-31	6.32e-40	6.92e-27	7.64e-20	2.12e-49	1.45e-06	8.48e-25	1.46e-29	1.29e-19	-	4.31e-14	1.29e-06	6.33e-08
**4-OH-CB187**	3.63e-01	4.65e-10	2.00e-15	2.01e-64	8.41e-92	5.58e-84	9.55e-131	6.26e-41	3.76e-56	2.22e-16	4.49e-99	3.08e-72	2.05e-02	5.73e-68	5.85e-53	1.18e-58	4.31e-14	-	1.85e-128	2.93e-97
**3-OH-CB153**	7.40e-06	4.15e-03	2.03e-06	6.21e-18	2.98e-23	2.28e-27	9.21e-44	1.28e-32	2.91e-19	1.36e-08	2.94e-31	2.40e-31	2.63e-01	2.88e-45	1.86e-29	5.34e-41	1.29e-06	1.85e-128	-	1.12e-79
**4-OH-CB107**	5.67e-03	6.99e-09	2.01e-11	3.82e-27	1.48e-29	1.62e-38	6.76e-60	1.67e-31	1.59e-30	1.03e-16	4.18e-52	1.76e-49	5.10e-06	2.34e-59	8.74e-45	6.30e-52	6.33e-08	2.93e-97	1.12e-79	-

Supplementary Table S56. Correlation coefficient for per- and polyfluorinated substances (PFAS) and hydroxyl polychlorinated biphenyls (OH-PCBs) correlations. Two-tailed Spearman's rank correlation test analysis on both controls and persons with MS.

	PFBS	PFPeS	PFHpA	PFHxS	PFHpS	PFOA	PFOS	FOSA	N-MeFOSAA	N-EtFOSAA	PFNA	PFDA	PFDS	PFUnA	PFDoA	PFTrDA	8:2 FTS	4-OH-CB187	3-OH-CB153	4-OH-CB107
**PFBS**	1	0.150	0.144	0.046	0.015	-0.038	-0.001	-0.055	-0.002	-0.066	0.026	0.028	0.042	-0.027	0.026	-0.068	-0.013	-0.021	-0.105	-0.065
**PFPeS**	0.150	1	0.429	0.450	0.244	0.358	0.246	0.075	0.120	0.089	0.261	0.224	0.110	0.200	0.195	0.189	0.101	0.146	0.067	0.135
**PFHpA**	0.144	0.429	1	0.286	0.230	0.490	0.279	0.219	0.191	0.159	0.365	0.348	0.082	0.282	0.311	0.246	0.201	0.185	0.111	0.157
**PFHxS**	0.046	0.45	0.286	1	0.809	0.630	0.669	0.288	0.263	0.174	0.512	0.521	0.115	0.387	0.311	0.271	0.181	0.383	0.201	0.250
**PFHpS**	0.015	0.244	0.230	0.809	1	0.704	0.829	0.326	0.363	0.221	0.574	0.525	0.114	0.368	0.325	0.245	0.200	0.451	0.230	0.261
**PFOA**	-0.038	0.358	0.490	0.630	0.704	1	0.683	0.313	0.362	0.270	0.676	0.603	0.084	0.428	0.417	0.336	0.29	0.433	0.251	0.298
**PFOS**	-0.001	0.246	0.279	0.669	0.829	0.683	1	0.420	0.450	0.282	0.713	0.707	0.192	0.618	0.566	0.509	0.287	0.528	0.317	0.370
**FOSA**	-0.055	0.075	0.219	0.288	0.326	0.313	0.420	1	0.471	0.357	0.311	0.404	0.139	0.391	0.383	0.351	0.268	0.307	0.274	0.269
**N-MeFOSAA**	-0.002	0.120	0.191	0.263	0.363	0.362	0.45	0.471	1	0.412	0.313	0.293	0.153	0.227	0.259	0.233	0.303	0.359	0.208	0.265
**N-EtFOSAA**	-0.066	0.089	0.159	0.174	0.221	0.270	0.282	0.357	0.412	1	0.172	0.199	0.119	0.147	0.147	0.138	0.248	0.191	0.133	0.193
**PFNA**	0.026	0.261	0.365	0.512	0.574	0.676	0.713	0.311	0.313	0.172	1	0.772	0.137	0.669	0.652	0.561	0.212	0.467	0.268	0.346
**PFDA**	0.028	0.224	0.348	0.521	0.525	0.603	0.707	0.404	0.293	0.199	0.772	1	0.208	0.812	0.787	0.644	0.337	0.404	0.269	0.337
**PFDS**	0.042	0.110	0.082	0.115	0.114	0.084	0.192	0.139	0.153	0.119	0.137	0.208	1	0.241	0.289	0.227	0.113	0.054	-0.026	0.107
**PFUnA**	-0.027	0.200	0.282	0.387	0.368	0.428	0.618	0.391	0.227	0.147	0.669	0.812	0.241	1	0.885	0.839	0.238	0.393	0.323	0.368
**PFDoA**	0.026	0.195	0.311	0.311	0.325	0.417	0.566	0.383	0.259	0.147	0.652	0.787	0.289	0.885	1	0.833	0.261	0.349	0.26	0.321
**PFTrDA**	-0.068	0.189	0.246	0.271	0.245	0.336	0.509	0.351	0.233	0.138	0.561	0.644	0.227	0.839	0.833	1	0.211	0.366	0.307	0.345
**8:2 FTS**	-0.013	0.101	0.201	0.181	0.200	0.290	0.287	0.268	0.303	0.248	0.212	0.337	0.113	0.238	0.261	0.211	1	0.176	0.113	0.127
**4-OH-CB187**	-0.021	0.146	0.185	0.383	0.451	0.433	0.528	0.307	0.359	0.191	0.467	0.404	0.054	0.393	0.349	0.366	0.176	1	0.524	0.463
**3-OH-CB153**	-0.105	0.067	0.111	0.201	0.230	0.251	0.317	0.274	0.208	0.133	0.268	0.269	-0.026	0.323	0.260	0.307	0.113	0.524	1	0.423
**4-OH-CB107**	-0.065	0.135	0.157	0.250	0.261	0.298	0.370	0.269	0.265	0.193	0.346	0.337	0.107	0.368	0.321	0.345	0.127	0.463	0.423	1

Supplementary Table S57. Description of treatments included in each treatment category.

Treatment category	Treatment
First line DMT	Aubagio, Avonex, Betaferon, Copaxone, Dimetylfumarat, Extavia, IVIG, Plegridy, Rebif, Skilarence
Second line DMT	Fingolimod, HSCT/BEAM, HSCT/CYK, Kesimpta, Lemtrada, Mavenclad, Mitoxantrone, Ocrevus, rituximab, Tysabri, Zinbryta
Other treatments	Aimovig, Arzerra IV, Dimetylfumarat, Deltison (Medrol), Solu-Medrol

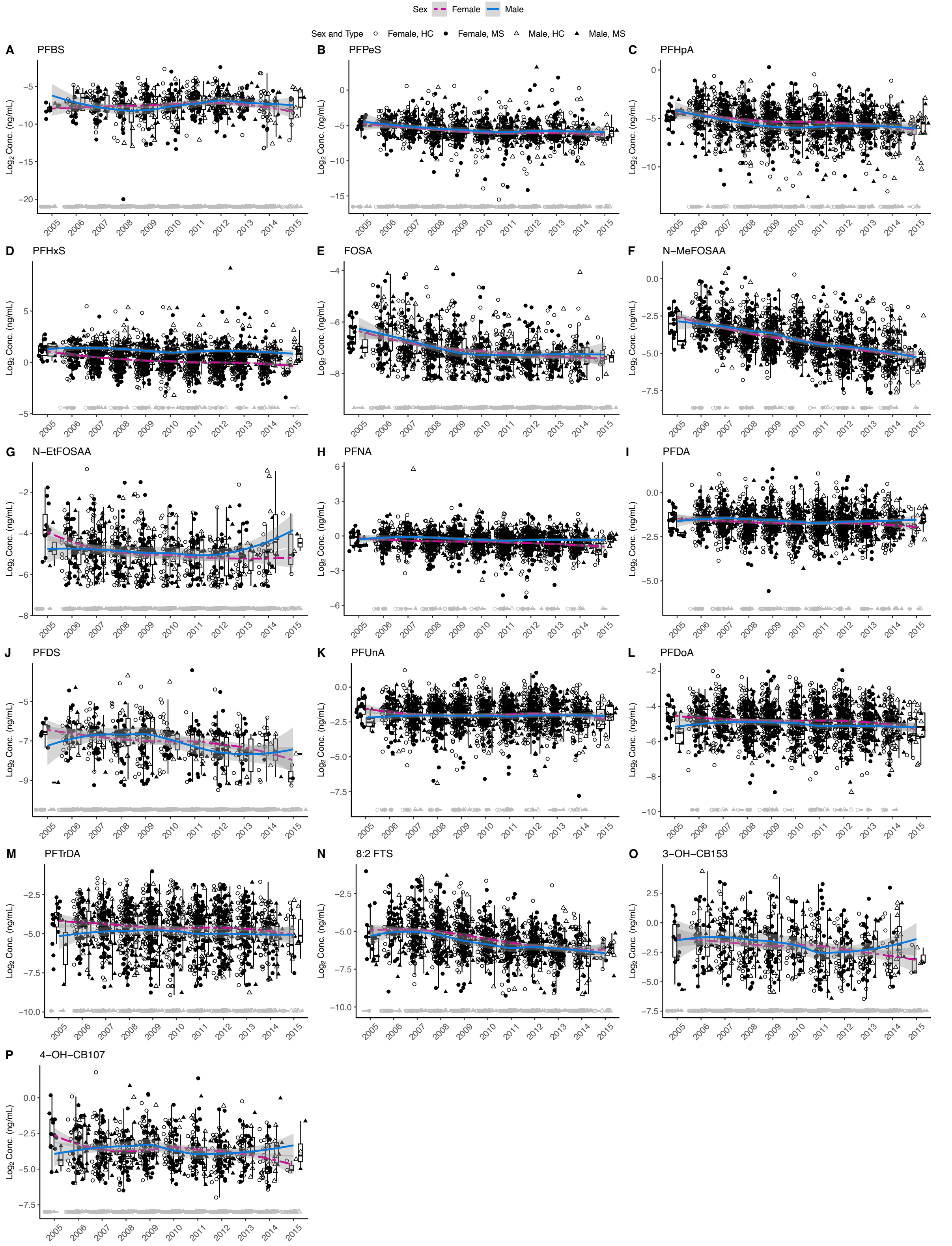

Supplementary Figure S1. Per- and polyfluorinated substance (PFAS) and hydroxyl polychlorinated biphenyls (OH-PCB) concentration (log$_2$ ng/mL) versus sample collection year of persons with multiple sclerosis (MS) and matched controls (HC), separated by sex. (A) PFBS, (B) PFPeS, (C) PFHpA, (D) PFHxS, (E) FOSA, (F) N-MeFOSAA, (G) N-EtFOSAA, (H) PFNA, (I) PFDA, (J) PFDS, (K) PFUnA, (L) PFDoA, (M) PFTrDA, (N) 8:2 FTS, (O) 3-OH-CB153, and (P) 4-OH-CB107. Plots contain concentration distribution for individuals with MS (solid shape) ($n = 907$) and HC subjects (empty shape) ($n = 907$), where men are represented by triangles and females by triangles. Imputed concentrations are shown in grey, and measured concentrations in black. The loess regression curve, including 95% confidence bands, has been fitted separately for males (blue solid line) and females (pink dashed line), excluding the imputed concentrations. The upper and lower whiskers indicate 1.5 times the interquartile range from above the upper quartile and below the lower quartile, respectively. Source data are provided as a Source Data file.

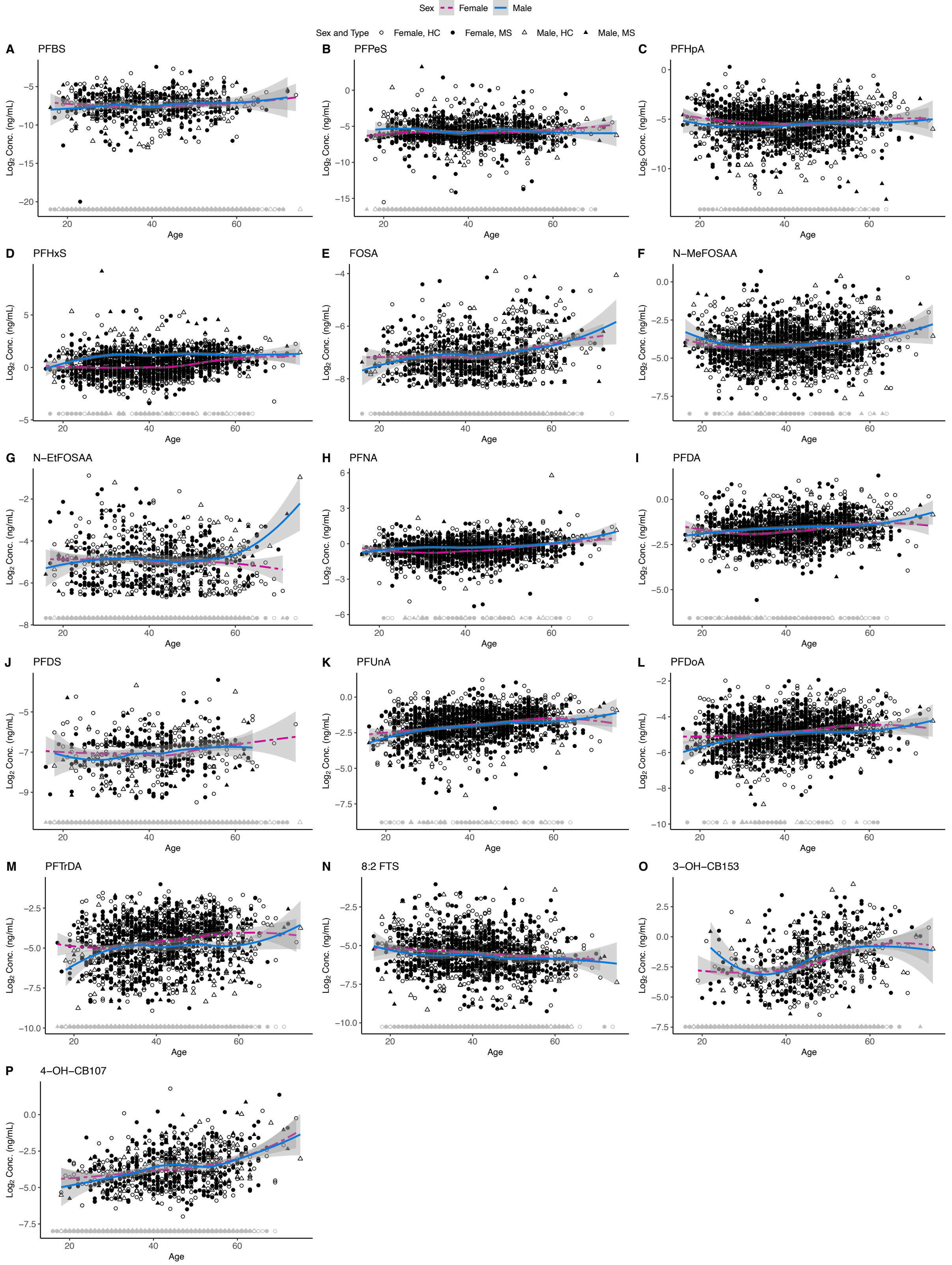

Supplementary Figure S2. Per- and polyfluorinated substance (PFAS) and hydroxyl polychlorinated biphenyls (OH-PCB) concentration ($\log_2$ ng/mL) versus age of persons with multiple sclerosis (MS) and matched controls (HC). (A) PFBS, (B) PFPeS, (C) PFHpA, (D) PFHxS, (E) FOSA, (F) N-MeFOSAA, (G) N-EtFOSAA, (H) PFNA, (I) PFDA, (J) PFDS, (K) PFUnA, (L) PFDoA, (M) PFTrDA, (N) 8:2 FTS, (O) 3-OH-CB153, and (P) 4-OH-CB107. Plots contain concentration distribution for individuals with MS (solid shapes) (n = 907) and HC subjects (empty shapes) (n = 907), where males are represented by triangles and females by circles. Imputed concentrations are shown in gray and measured concentrations in black. The loess regression curve, including 95% confidence band, has been fitted separately for males (blue solid line) and females (pink dashed line), excluding the imputed concentrations. Source data are provided as a Source Data file.

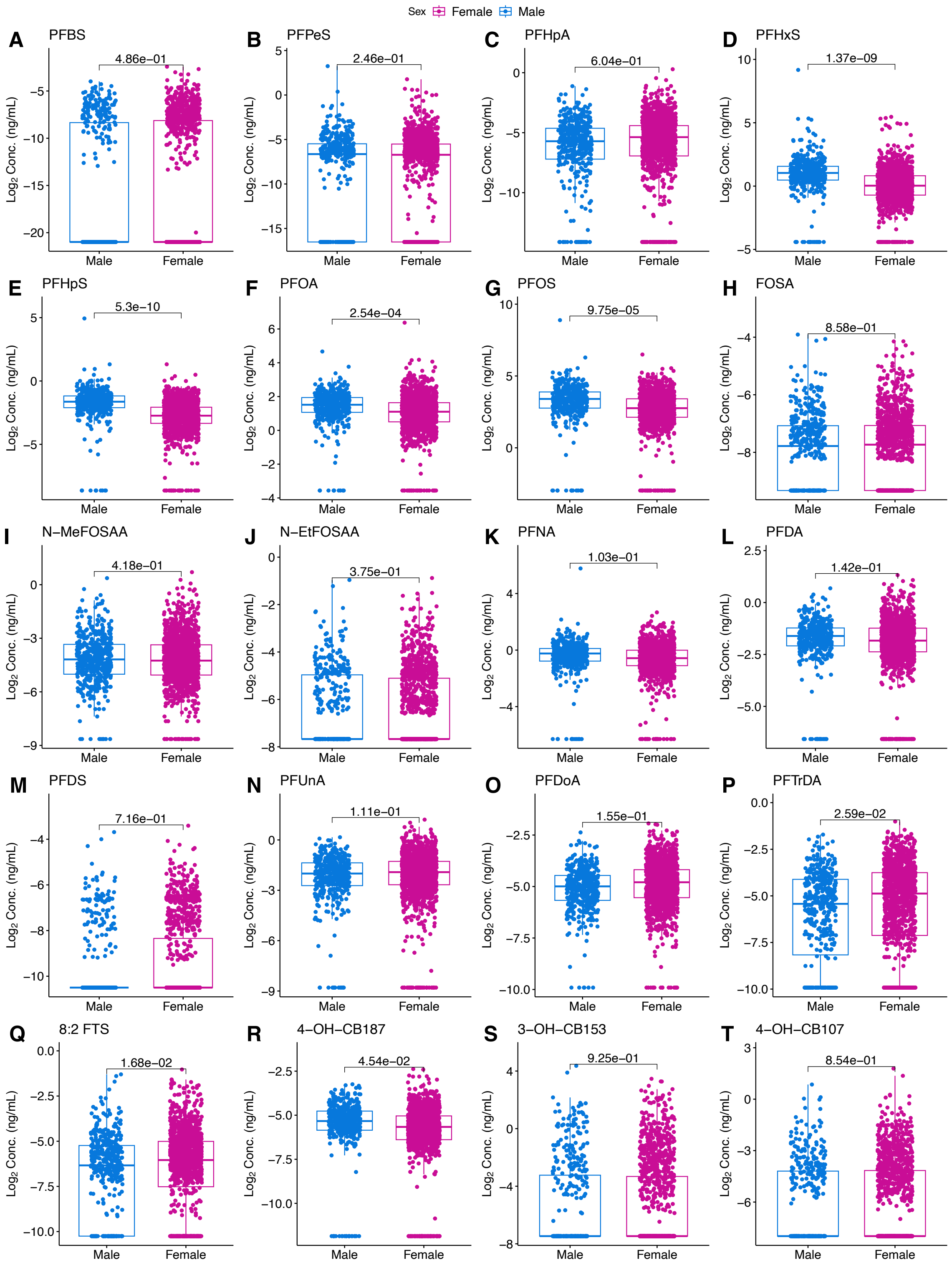

Supplementary Figure S3. Per- and polyfluorinated substance (PFAS) and hydroxyl polychlorinated biphenyls (OH-PCB) concentration (log_2 ng/mL) in males and females. (A) PFBS, (B) PFPeS, (C) PFHpA, (D) PFHxS, (E) PFHpS, (F) PFOA, (G) PFOS, (H) FOSA, (I) N-MeFOSAA, (J) N-EtFOSAA, (K) PFNA, (L) PFDA, (M) PFDS, (N) PFUnA, (O) PFDoA, (P) PFTrDA, (Q) 8:2 FTS, (R) 4-OH-CB187, (S) 3-OH-CB153, and (T) 4-OH-CB107. Individuals with multiple sclerosis (MS) ($n = 907$) and matched controls ($n = 907$) are included in the plot. The paired comparisons was calculated using estimated marginal means based on a linear mixed-effect regression model and *P* values, shown above each bar, for the two-sided tests were computed using Satterthwaite's degrees of freedom method. No adjustments were made for multiple comparisons. The model was adjusted for age, BMI at the age of 20, BMI at sample time point, year of sample collection, years of regular smoking, years of irregular smoking, years of indoor passive smoking, number of childbirths, and type of treatment. The upper and lower whiskers indicate 1.5 times the interquartile range from above the upper quartile and below the lower quartile, respectively. Source data are provided as a Source Data file.

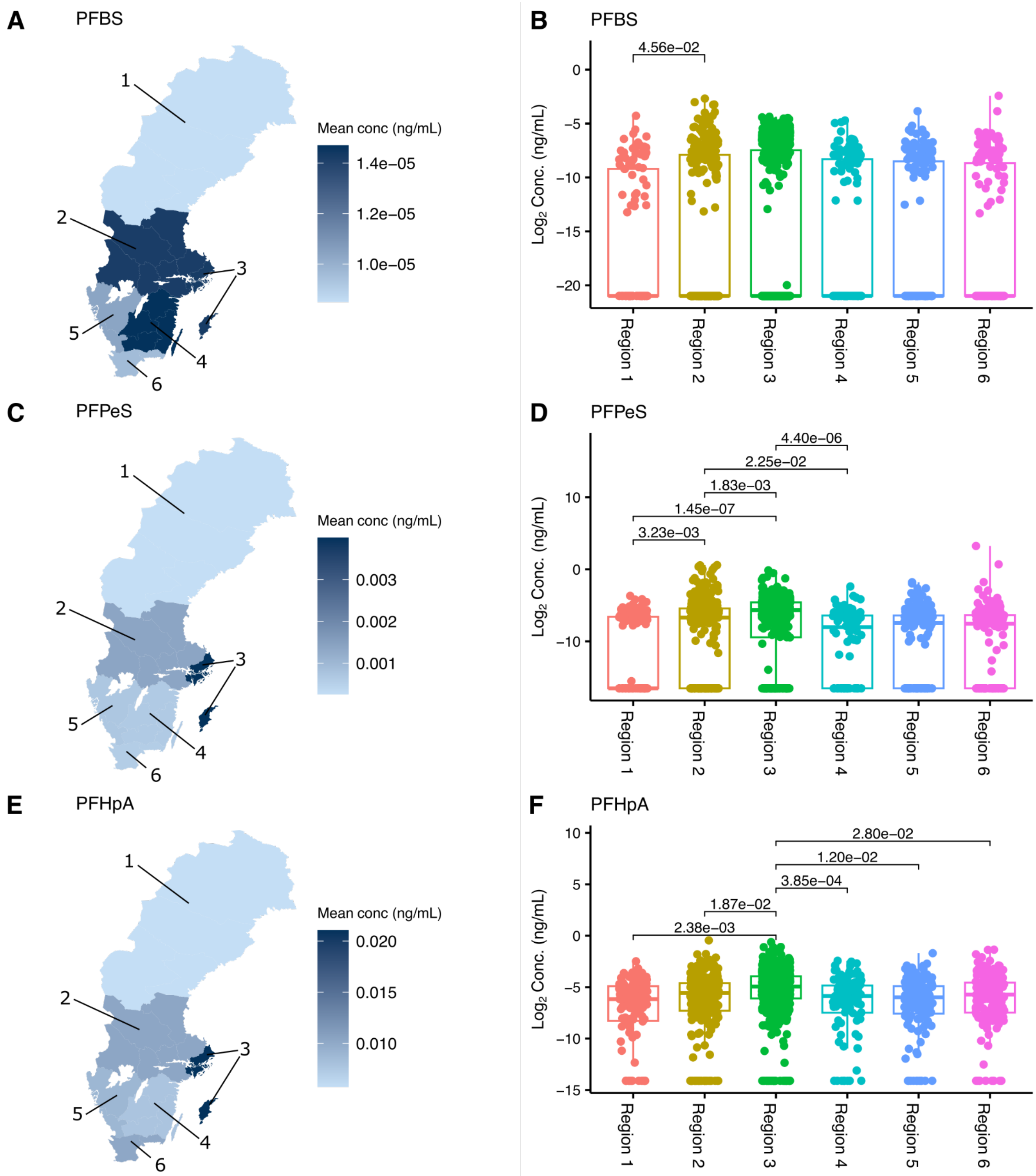

Supplementary Figure S4. Perfluorobutanesulfonic acid (PFBS), perfluoropentanesulfonic acid (PFPeS), and perfluoroheptanoic acid (PFHpA) concentration distribution across Sweden. (A, C, and E) Illustrates the mean concentration (ng/mL) for the six larger regions. (B, D, and F) Concentration distribution ($\log_2$ ng/mL) and the *P* values region comparisons. Analysis was made on individuals with MS (n = 907) and matched controls (n = 907), and comparisons were made between all six regions using estimated marginal means based on a linear mixed-effect regression model and *P* values for the two-sided tests were computed using Satterthwaite's degrees of freedom method. Only significant *P* values were included in the boxplot. The model was adjusted for age, sex, sample collection year, BMI at sample time point, BMI at the age of 20, undergoing treatment, years of regular smoking, years of irregular smoking, years of passive smoking (indoor environment), disease phenotype, and number of childbirths. Moreover, the upper and lower whiskers indicate 1.5 times the interquartile range from above the upper quartile and below the lower quartile, respectively. Source data are provided as a Source Data file.

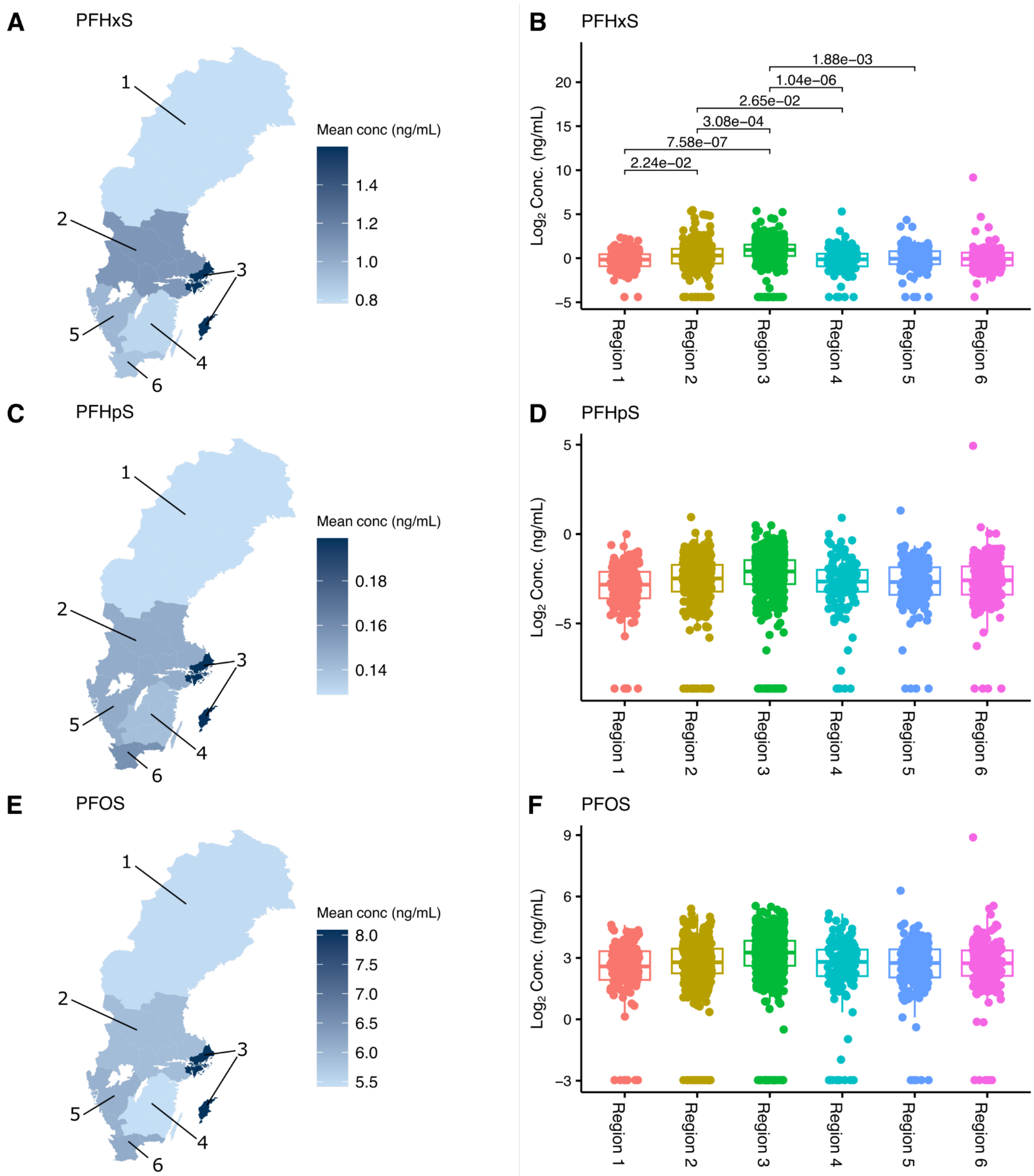

Supplementary Figure S5. **Perfluorohexanesulfonic acid (PFHxS), perfluoroheptanesulfonic acid (PFHpS), and perfluorooctanesulfonic acid (PFOS) concentration distribution across Sweden.** (A, C, an E) Illustrates the mean concentration (ng/mL) for the six larger regions. (B, D, and F) Concentration distribution (log_2 ng/mL) and the *P* values region comparisons. Analysis was made on individuals with MS (n = 907) and matched controls (n = 907), and comparisons were made between all six regions using estimated marginal means based on a linear mixed-effect regression model and *P* values for the two-sided tests were computed using Satterthwaite's degrees of freedom method. Only significant *P* values were included in the boxplot. The model was adjusted for age, sex, sample collection year, BMI at sample time point, BMI at the age of 20, undergoing treatment, years of regular smoking, years of irregular smoking, years of passive smoking (indoor environment), disease phenotype, and number of childbirths. Moreover, the upper and lower whiskers indicate 1.5 times the interquartile range from above the upper quartile and below the lower quartile, respectively. Source data are provided as a Source Data file.

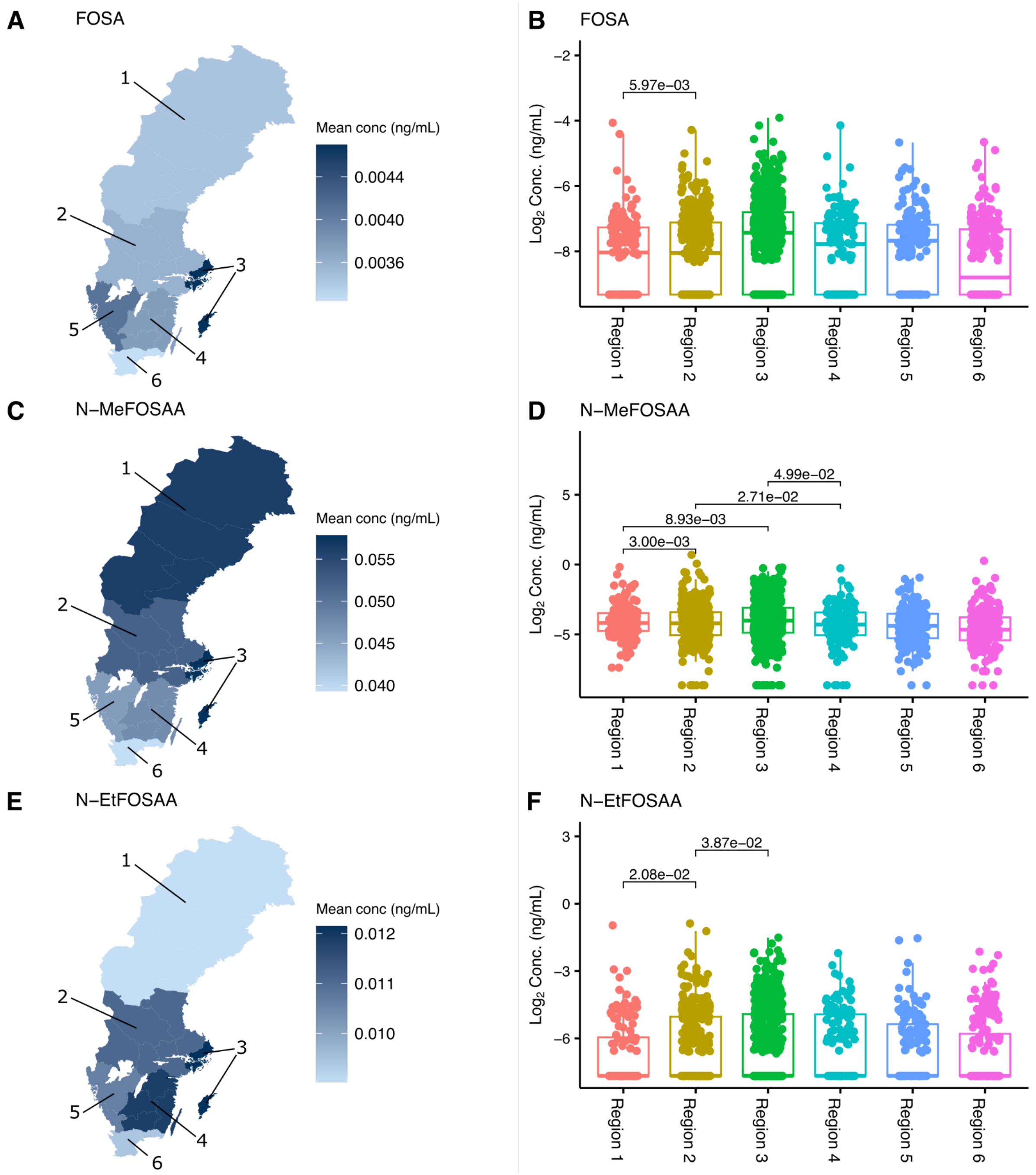

Supplementary Figure S6. **Perfluoro-1-octanesulfonamide (FOSA), N-methylperfluoro-1-octanesulfonamidoacetic acid (N-MeFOSAA), and N-ethylperfluoro-1-octanesulfonamidoacetic acid (N-EtFOSAA) concentration distribution across Sweden.** (A, C, and E) Illustrates the mean concentration (ng/mL) for the six larger regions. (B, D, and F) Concentration distribution ($\log_2$ ng/mL) and the *P* values region comparisons. Analysis was made on individuals with MS (n = 907) and matched controls (n = 907), and comparisons were made between all six regions using estimated marginal means based on a linear mixed-effect regression model and *P* values for the two-sided tests were computed using Satterthwaite's degrees of freedom method. Only significant *P* values were included in the boxplot. The model was adjusted for age, sex, sample collection year, BMI at sample time point, BMI at the age of 20, undergoing treatment, years of regular smoking, years of irregular smoking, years of passive smoking (indoor environment), disease phenotype, and number of childbirths. Moreover, the upper and lower whiskers indicate 1.5 times the interquartile range from above the upper quartile and below the lower quartile, respectively. Source data are provided as a Source Data file.

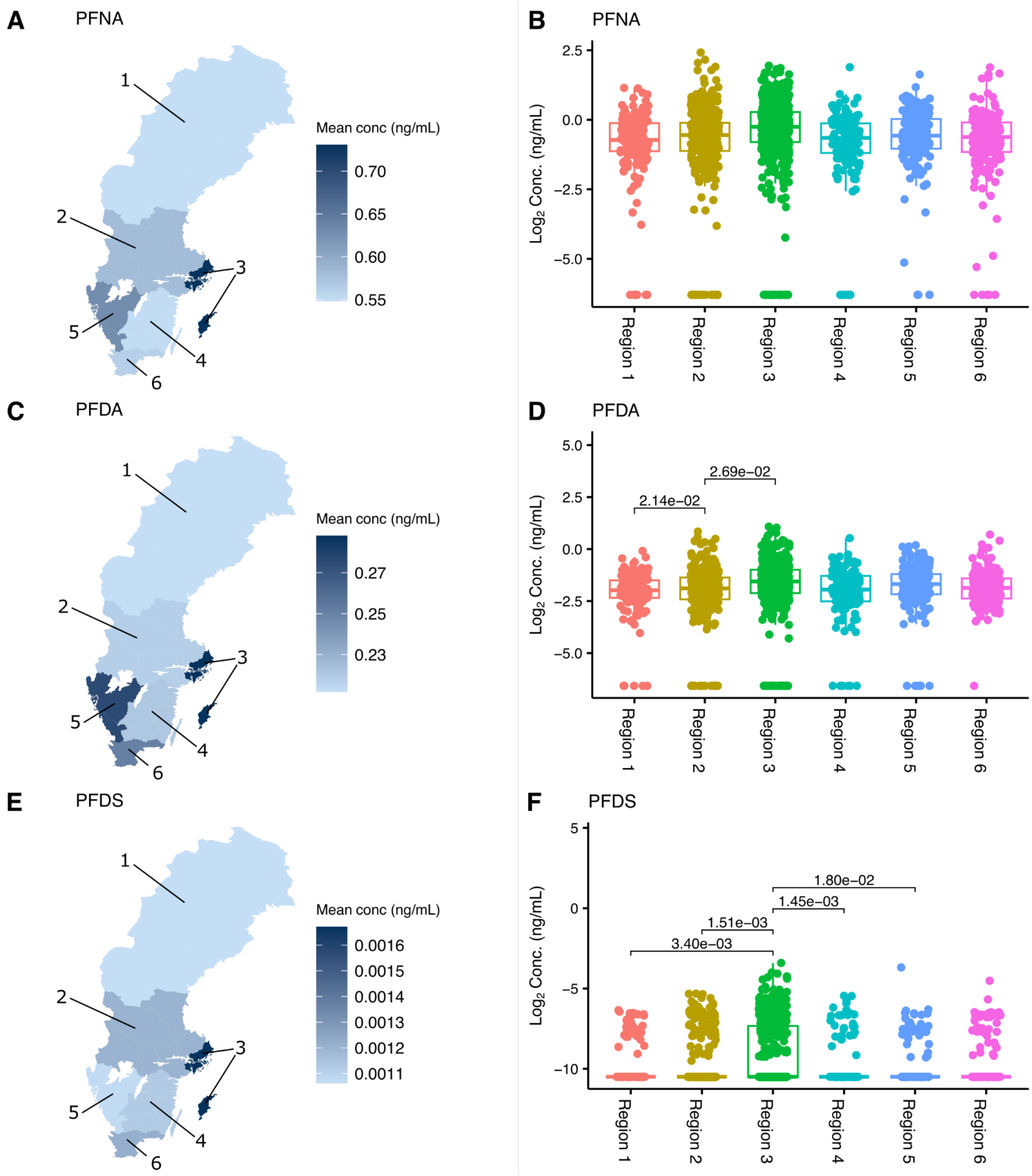

Supplementary Figure S7. **Perfluorononanoic acid (PFNA), perfluorodecanoic acid (PFDA), and perfluorodecane sulfonic acid (PFDS) concentration distribution across Sweden.** (A, C, and E) Illustrates the mean concentration (ng/mL) for the six larger regions. (B, D, and F) Concentration distribution ($\log_2$ ng/mL) and the *P* values region comparisons. Analysis was made on individuals with MS (n = 907) and matched controls (n = 907), and comparisons were made between all six regions using estimated marginal means based on a linear mixed-effect regression model and *P* values for the two-sided tests were computed using Satterthwaite's degrees of freedom method. Only significant *P* values were included in the boxplot. The model was adjusted for age, sex, sample collection year, BMI at sample time point, BMI at the age of 20, undergoing treatment, years of regular smoking, years of irregular smoking, years of passive smoking (indoor environment), disease phenotype, and number of childbirths. Moreover, the upper and lower whiskers indicate 1.5 times the interquartile range from above the upper quartile and below the lower quartile, respectively. Source data are provided as a Source Data file.

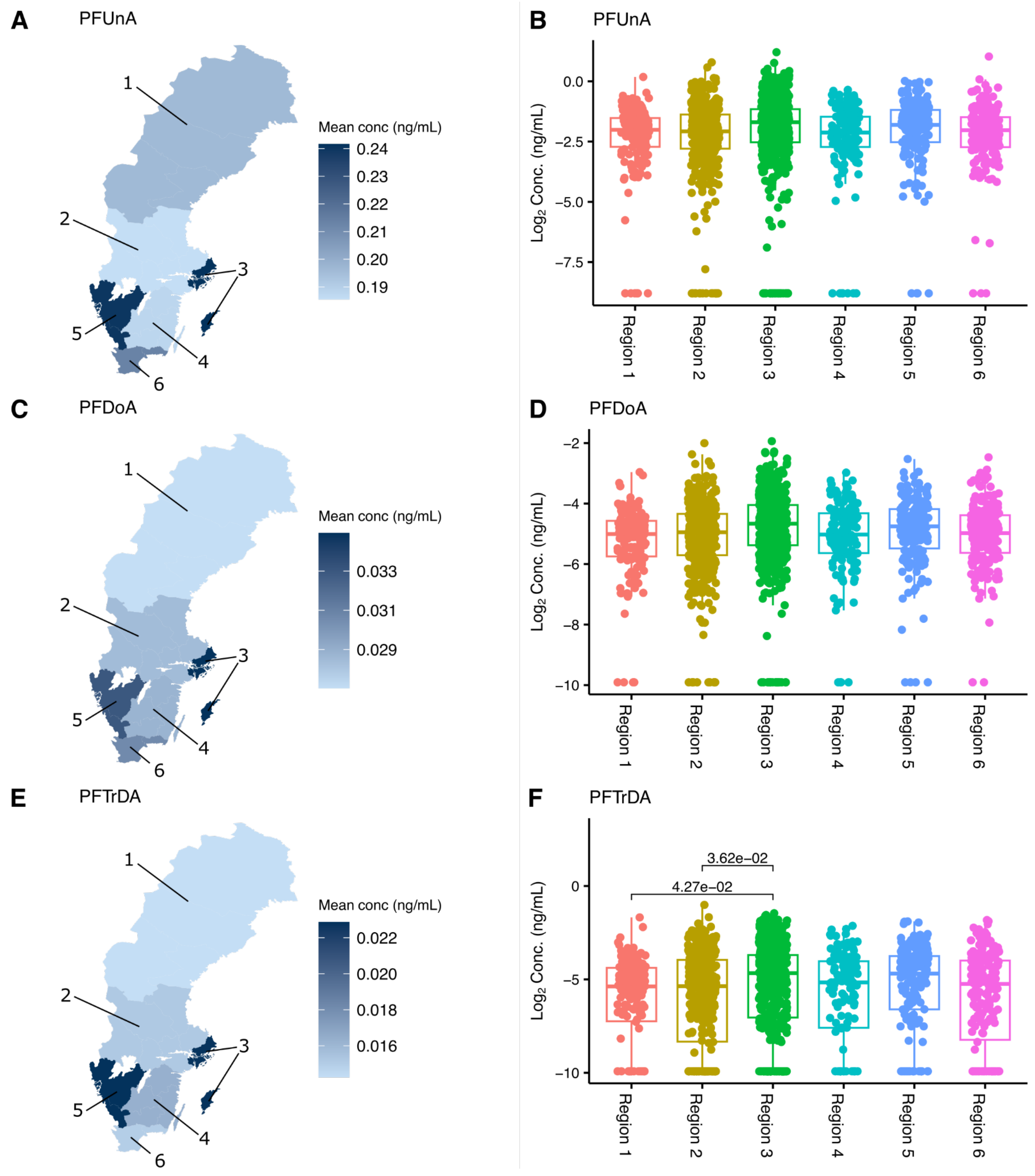

Supplementary Figure S8. **Perfluoroundecanoic acid (PFUnA), perfluorododecanoic acid (PFDoA), and perfluorotridecanoic acid (PFTrDA) concentration distribution across Sweden.** (A, C, and E) Illustrates the mean concentration (ng/mL) for the six larger regions. (B, D, and F) Concentration distribution (log_2 ng/mL) and the *P* values region comparisons. Analysis was made on individuals with MS (n = 907) and matched controls (n = 907), and comparisons were made between all six regions using estimated marginal means based on a linear mixed-effect regression model and *P* values for the two-sided tests were computed using Satterthwaite's degrees of freedom method. Only significant *P* values were included in the boxplot. The model was adjusted for age, sex, sample collection year, BMI at sample time point, BMI at the age of 20, undergoing treatment, years of regular smoking, years of irregular smoking, years of passive smoking (indoor environment), disease phenotype, and number of childbirths. Moreover, the upper and lower whiskers indicate 1.5 times the interquartile range from above the upper quartile and below the lower quartile, respectively. Source data are provided as a Source Data file.

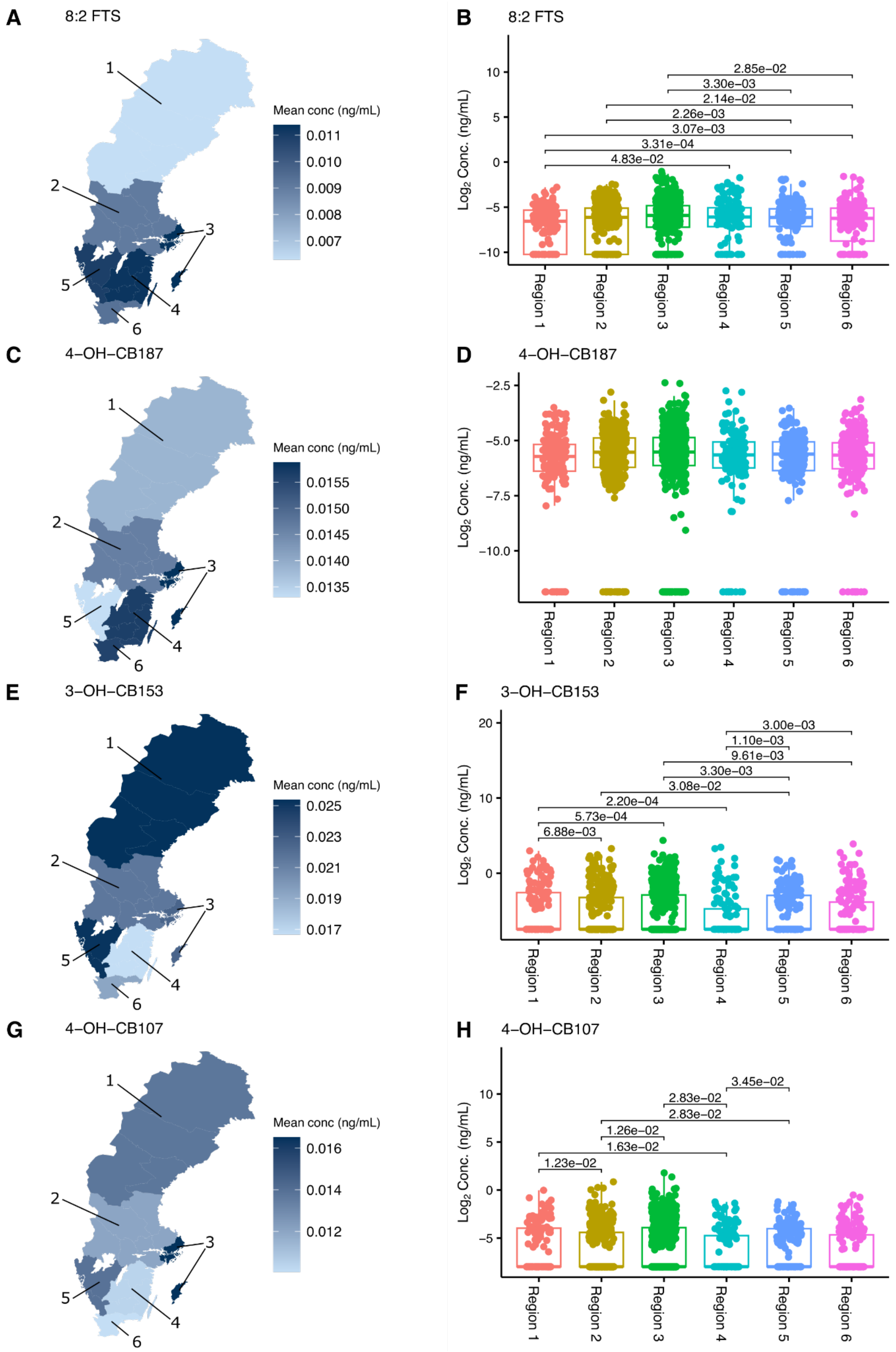

A
8:2 FTS
Mean conc (ng/mL)
0.011
0.010
0.009
0.008
0.007
B
8:2 FTS
Log2 Conc. (ng/mL)
2.85e−02
3.30e−03
2.14e−02
2.26e−03
3.07e−03
3.31e−04
4.83e−02
Region 1
Region 2
Region 3
Region 4
Region 5
Region 6
C
4−OH−CB187
Mean conc (ng/mL)
0.0155
0.0150
0.0145
0.0140
0.0135
D
4−OH−CB187
Log2 Conc. (ng/mL)
E
3−OH−CB153
Mean conc (ng/mL)
0.025
0.023
0.021
0.019
0.017
F
3−OH−CB153
Log2 Conc. (ng/mL)
3.00e−03
1.10e−03
9.61e−03
3.30e−03
3.08e−02
2.20e−04
5.73e−04
6.88e−03
G
4−OH−CB107
Mean conc (ng/mL)
0.016
0.014
0.012
H
4−OH−CB107
Log2 Conc. (ng/mL)
3.45e−02
2.83e−02
2.83e−02
1.26e−02
1.63e−02
1.23e−02

Supplementary Figure S9. **1h,1h,2h,2h-perfluoro-1-decanesulfonic acid (8:2) (8:2 FTS), 2,2',3,4',5,5',6-heptachloro-4-biphenylol (4-OH-CB187), 2,2',4,4',5,5'-,Hexachloro-3-biphenylol (3-OH-CB153), and 2,3,3',4',5-Pentachloro-4-biphenylol (4-OH-CB107) concentration distribution across Sweden.** (A, C, E, and G) Illustrates the mean concentration (ng/mL) for the six larger regions. (B, D, F, and H) Concentration distribution ($\log_2$ ng/mL) and the *P* values region comparisons. Analysis was made on individuals with MS (n = 907) and matched controls (n = 907), and comparisons were made between all six regions using estimated marginal means based on a linear mixed-effect regression model and *P* values for the two-sided tests were computed using Satterthwaite's degrees of freedom method. Only significant *P* values were included in the boxplot. The model was adjusted for age, sex, sample collection year, BMI at sample time point, BMI at the age of 20, undergoing treatment, years of regular smoking, years of irregular smoking, years of passive smoking (indoor environment), disease phenotype, and number of childbirths. Moreover, the upper and lower whiskers indicate 1.5 times the interquartile range from above the upper quartile and below the lower quartile, respectively. Source data are provided as a Source Data file.

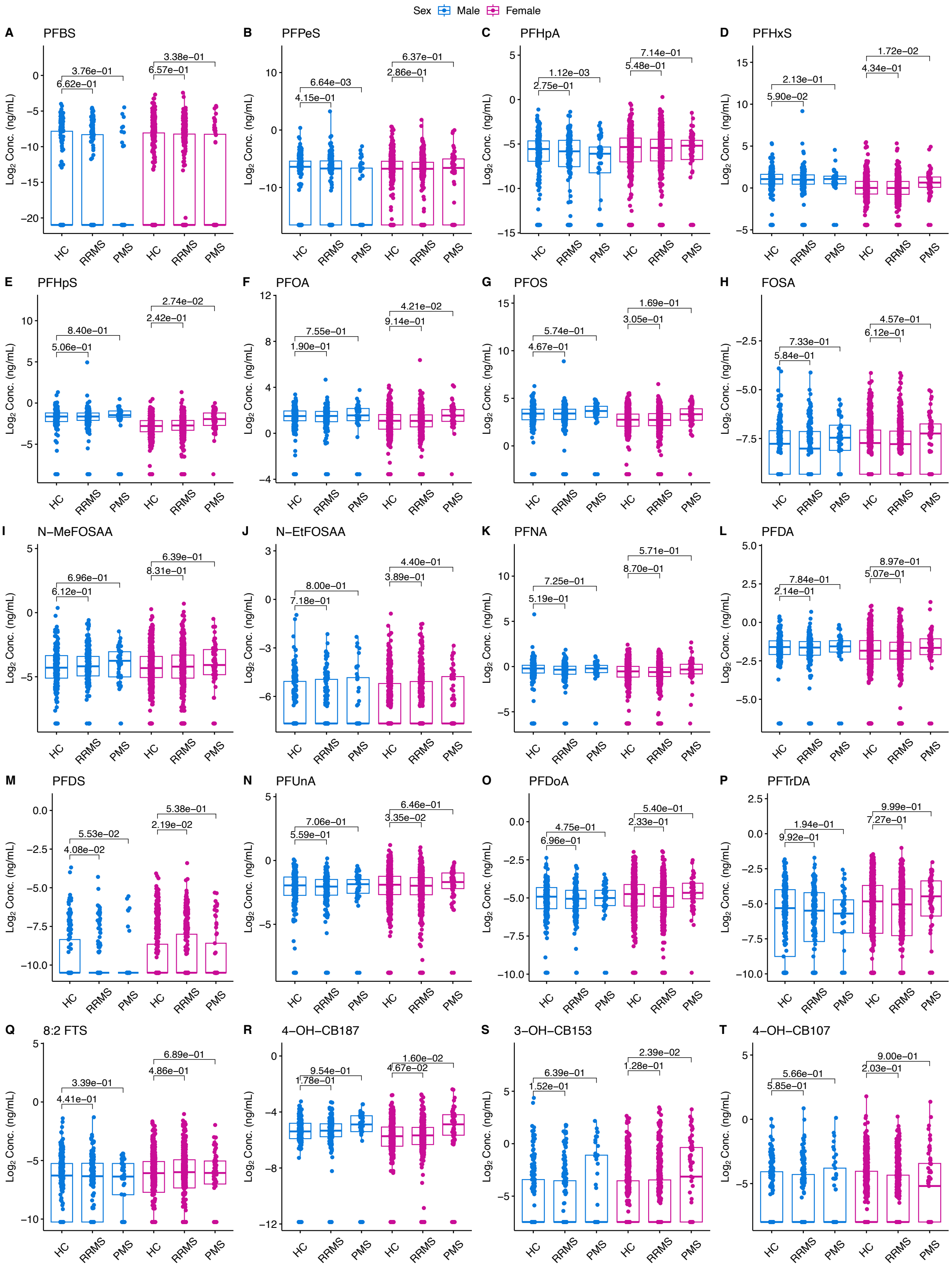

Supplementary Figure S10. Concentration distribution of per- and polyfluorinated substances (PFAS) and hydroxyl polychlorinated biphenyls (OH-PCBs) in multiple sclerosis (MS) subjects and matched control (HC) divided by sex. (A) PFBS, (B) PFPeS, (C) PFHpA, (D) PFHxS, (E) PFHpS, (F) PFOA, (G) PFOS, (H) FOSA, (I) N-MeFOSAA, (J) N-EtFOSAA, (K) PFNA, (L) PFDA, (M) PFDS, (N) PFUnA, (O) PFDoA, (P) PFTrDA, (Q) 8:2 FTS, (R) 4-OH-CB187, (S) 3-OH-CB153, and (T) 4-OH-CB107. Relapsing-remitting MS (RRMS) (n = 801) and progressive MS (PMS) (n = 106) are compared with HC subjects (n = 907). Blue represents male subjects and pink female subjects. The paired comparisons was calculated using estimated marginal means based on a linear mixed-effect regression model and P values, shown above each bar, for the two-sided tests were computed using Satterthwaite's degrees of freedom method. The model was adjusted for age, sex, BMI at the age of 20, BMI at sample time point, year of sample collection, years of regular smoking, years of irregular smoking, years of indoor passive smoking, number of childbirths, and type of treatment. No adjustments were made for multiple comparisons. The upper and lower whiskers indicate 1.5 times the interquartile range from above the upper quartile and below the lower quartile, respectively. Source data are provided as a Source Data file.

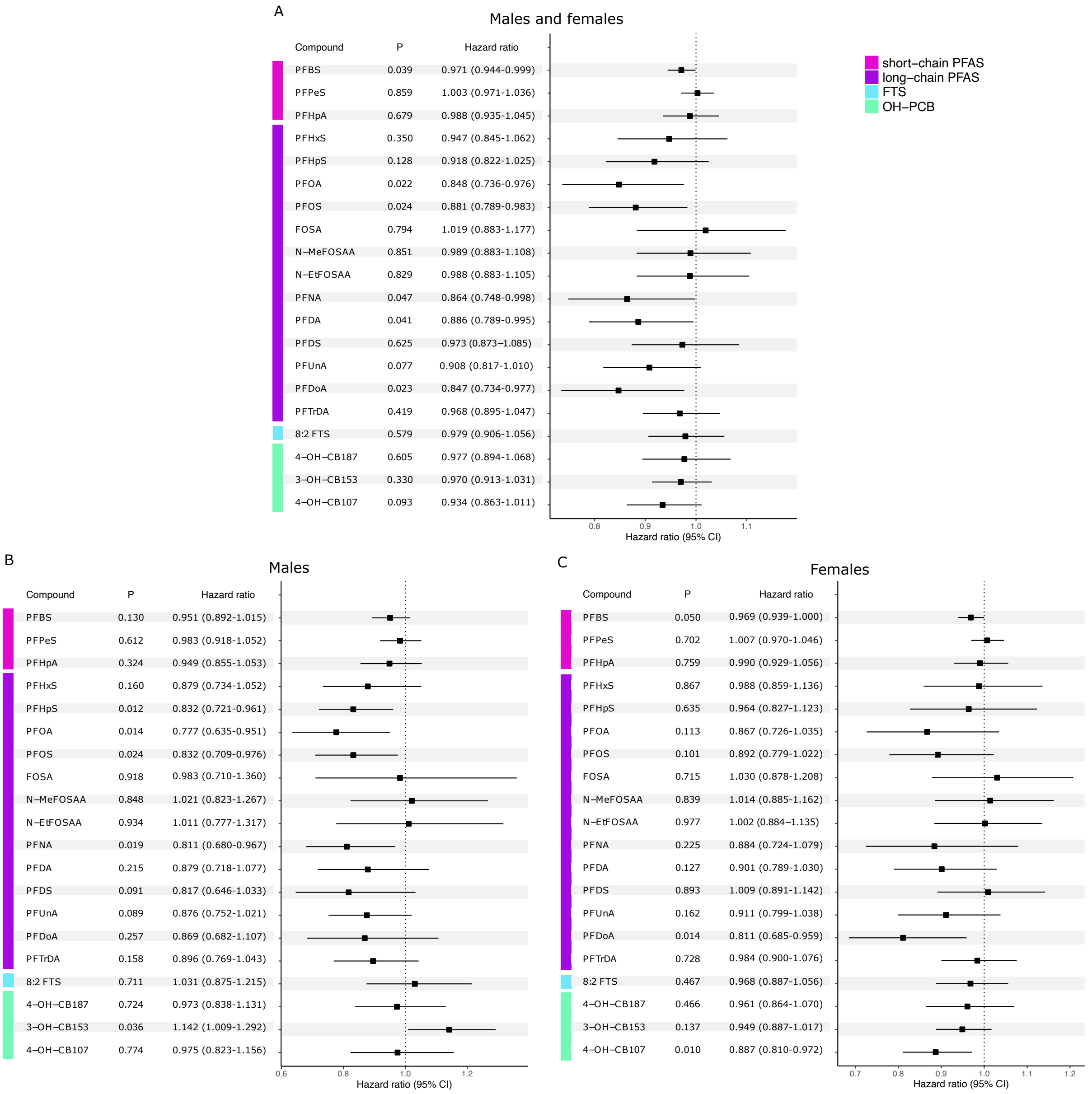

Supplementary Figure S11. Per- and polyfluorinated substances (PFAS) and hydroxyl polychlorinated biphenyls (OH-PCBs) association with confirmed disability worsening adjusting for 25(OH)D3 levels. *P* values, coefficients, hazard ratios (HR), and the 95% confidence interval (CI) for each compound were estimated using Cox proportional hazard analysis. No adjustments were made for multiple comparisons. The hazard ratio value is represented by the square and error bars represent its 95% CI. Analysis based on (A) the entire group (n = 551), (B) males only (n = 137), and (C) females only (n = 414). PFAS compounds have been grouped into three groups: short-chain PFAS (pink), long-chain PFAS (purple), and FTS (blue), while OH-PCBs (green) are listed separately. Source data are provided as a Source Data file.

Compound	P	Hazard ratio
PFBS	0.327	0.979 (0.939-1.021)
PFPeS	0.761	1.008 (0.957-1.061)
PFHpA	0.212	0.951 (0.879-1.029)
PFHxS	0.547	1.057 (0.883-1.266)
PFHpS	0.143	1.175 (0.947-1.458)
PFOA	0.762	1.038 (0.816-1.320)
PFOS	0.366	1.097 (0.897-1.341)
FOSA	0.882	0.984 (0.800-1.211)
N–MeFOSAA	0.733	1.030 (0.870-1.219)
N–EtFOSAA	0.559	1.050 (0.891-1.237)
PFNA	0.88	1.018 (0.810-1.279)
PFDA	0.533	1.068 (0.868-1.314)
PFDS	0.409	0.933 (0.791-1.100)
PFUnA	0.464	0.939 (0.793-1.111)
PFDoA	0.076	0.817 (0.654-1.022)
PFTrDA	0.784	1.017 (0.903-1.145)
8:2 FTS	0.724	0.980 (0.874-1.098)
4–OH–CB187	0.356	1.086 (0.912-1.294)
3–OH–CB153	0.826	1.010 (0.926-1.101)
4–OH–CB107	0.822	1.013 (0.906-1.133)

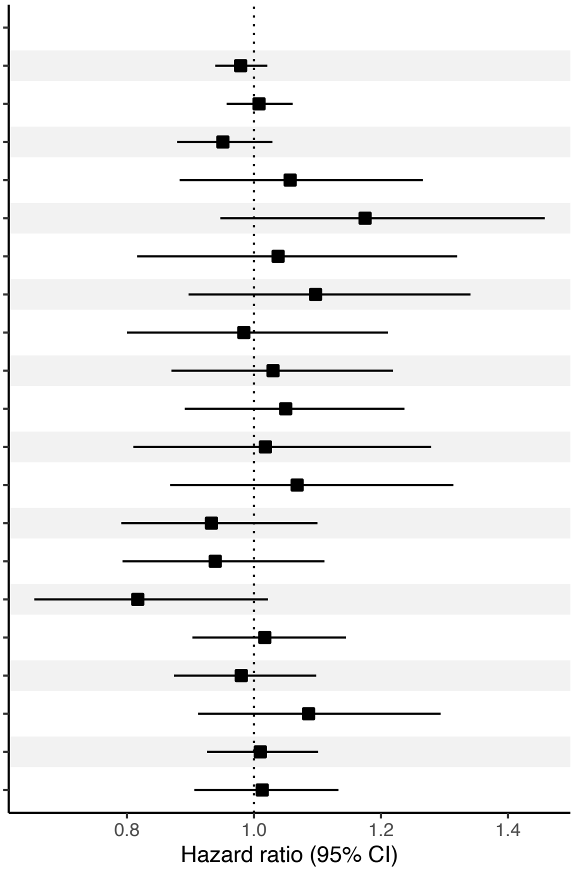

short–chain PFAS long–chain PFAS FTS OH–PCB

Supplementary Figure S12. **Per- and polyfluorinated substances (PFAS) and hydroxyl polychlorinated biphenyls (OH-PCBs) association with the transition from relapsing-remitting multiple sclerosis (RRMS) to secondary progressive multiple sclerosis (SPMS) (n = 801) adjusted for 25(OH)D3 levels.** *P* values, coefficients, hazard ratios (HR), and the 95% confidence interval (CI) for each compound were estimated using Cox proportional hazard analysis. No adjustments were made for multiple comparisons. The hazard ratio value is represented by the square and error bars represent its 95% CI. PFAS compounds have been grouped into three groups: short-chain PFAS (pink), long-chain PFAS (purple), and FTS (blue), while OH-PCBs (green) are listed separately. Source data are provided as a Source Data file.

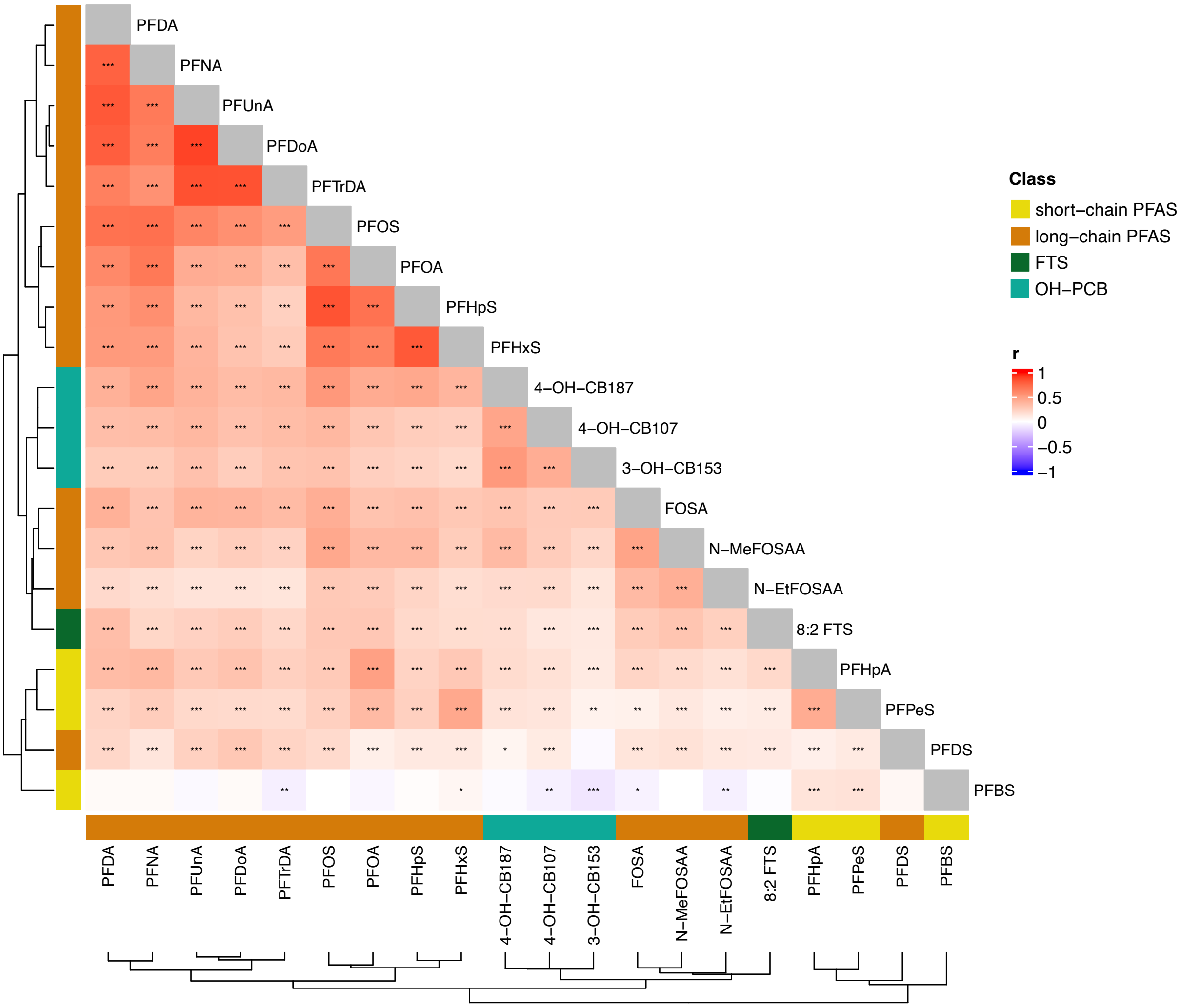

Supplementary Figure S13. Spearman's rank correlation test between per- and polyfluorinated substances (PFAS) and hydroxyl polychlorinated biphenyls (OH-PCBs). Correlation analysis was made both on controls (n = 907) and persons with multiple sclerosis (MS) (n = 907). The intensity of colours represents the regression coefficient. PFAS compounds have been grouped into three groups: short-chain PFAS (yellow), long-chain PFAS (orange), and FTS (green), while OH-PCBs (turquoise) are listed separately. Source data are provided as a Source Data file.
* P value < 0.05; ** P value < 0.01; *** P value < 0.001

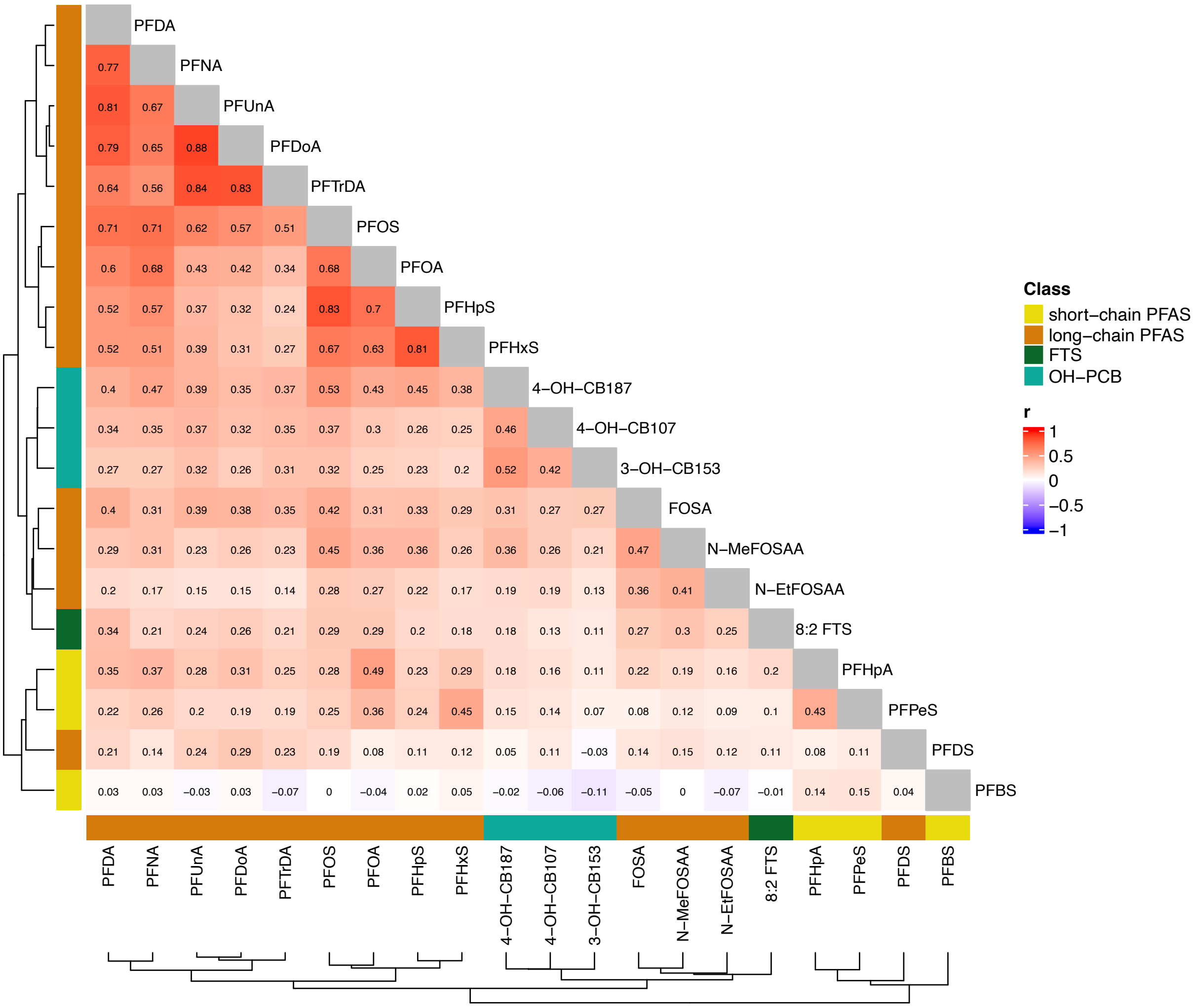

Supplementary Figure S14. Spearman's rank correlation test between per- and polyfluorinated substances (PFAS) and hydroxyl polychlorinated biphenyls (OH-PCBs). Correlation analysis was made both on controls (n = 907) and persons with multiple sclerosis (MS) (n = 907). Values in each cell represent the correlation coefficient r. The intensity of colours represents the regression coefficient. PFAS compounds have been grouped into three groups: short-chain PFAS (yellow), long-chain PFAS (orange), and FTS (green), while OH-PCBs (turquoise) are listed separately. Source data are provided as a Source Data file.
